# A Chemoenzymatic Cascade for the Formal Enantioselective Hydroxylation and Amination of Benzylic C–H Bonds

**DOI:** 10.1021/acscatal.4c03161

**Published:** 2024-11-12

**Authors:** Yuqing Zhang, Chen Huang, Weixi Kong, Liya Zhou, Jing Gao, Frank Hollmann, Yunting Liu, Yanjun Jiang

**Affiliations:** † School of Chemical Engineering and Technology, Hebei University of Technology, Tianjin 300401, China; ‡ Department of Biotechnology, Delft University of Technology, 2629 HZ Delft, The Netherlands

**Keywords:** single-atom catalysts, artificial peroxygenase, continuous flow, enantioselective C−H functionalization

## Abstract

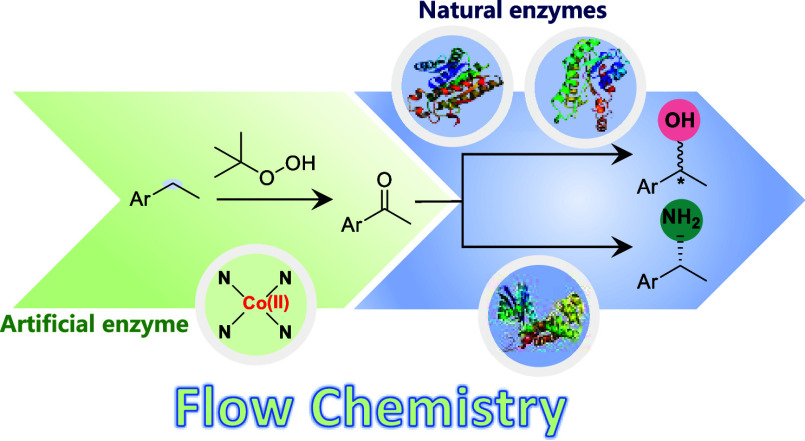

We report the synthesis and characterization of an artificial peroxygenase (CoN_4_SA-POase) with CoN_4_ active sites by supporting single-atom cobalt on polymeric carbon nitrogen, which exhibits high activity, selectivity, stability, and reusability in the oxidation of aromatic alkanes to ketones. Density functional theory calculations reveal a different catalytic mechanism for the artificial peroxygenase from that of natural peroxygenases. In addition, continuous-flow systems are employed to combine CoN_4_SA-POase with enantiocomplementary ketoreductases as well as an amine dehydrogenase, enabling the enantioselective synthesis of chiral alcohols and amines from hydrocarbons with significantly improved productivity. This work, emulating nature and beyond nature, provides a promising design concept for heme enzyme-based transformations.

## Introduction

Selective C–H oxidation of hydrocarbons into value-added oxyfunctionalized compounds, such as alcohols, ketones, aldehydes, and acids, is of great significance in the chemical industry but has historically been problematic due to the high dissociation energy of the inert C(sp^3^)–H bonds.^1^ Nature has evolved oxygenases to address this challenge by recruiting transition metals and heme (iron protoporphyrin IX) into enzyme active sites,^2^ particularly including the well-known cytochrome P450 monooxygenases (P450s)^3^ and the emerging unspecific peroxygenases (UPOs).^4^ Both enzyme classes rely on an oxoferryl-heme cation radical complex (the so-called Compound I, CpdI) to oxygenate C–H bonds. Despite their undoubted potential for organic synthesis, these enzymes, however, are not widely used in organic synthesis. Issues such as low substrate loadings and poor catalyst performance (especially in terms of robustness) remain to be solved.^5^

Chemocatalysts, on the other hand, are less plagued by these issues but suffer from a lack of enantioselectivity, which is highly demanded in the synthesis of active pharmaceutical ingredients or agrochemicals. Metal-based heterogeneous catalysts mediating C–H oxidation represent excellent substitutes for enzymatic alternatives due to their high stability and ease of handling and recycling, especially if the overoxidation to the achiral ketone is desired.^6^ Especially, single-atom catalysts (SACs) have attracted attention due to their extraordinary catalytic performance compared to metal nanoparticles.^7^ However, due to their higher surface energy, SACs suffer from aggregation. To prevent the undesired aggregation, nitrogen-containing materials such as N-doped graphene, carbon nanotubes, or carbon nitrides have been demonstrated to stabilize mononuclear metal complexes (MN_*x*_, M = Fe, Co, Ni, etc.).^8^ The FeN_4_ structure in the active centers of heme-containing enzymes provides a template for the design of heterogeneous SACs with heme enzyme-like catalytic activity. Particularly, advances have been made in developing artificial peroxidase activity, i.e., utilizing H_2_O_2_ to oxidize substrates.^9^ In contrast, reports on artificial peroxygenase activity (i.e., insertion of an oxygen atom) are yet rare.^10^

**Scheme 1:**
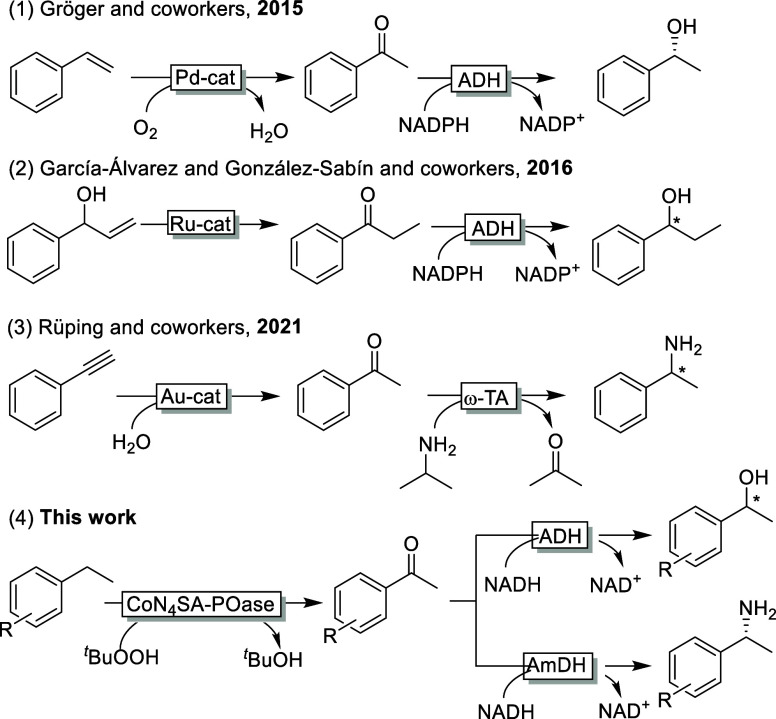
chemoenzymatic Approaches to Synthesize Benzyl Alcohols and Amines

In this contribution, we envisioned combining heterogeneous catalysis for the selective oxyfunctionalization of benzylic CH_2_ groups to the corresponding acetophenone derivates followed by their enantioselective reduction or reductive amination. Hence, we aimed at combining the best of two worlds, the high activity and robustness of heterogeneous catalysis with the high stereoselectivity of enzymatic reactions.^11^ In contrast to previously reported chemoenzymatic reactions such as Pd/Cu-catalyzed Wacker oxidations of styrenes,^12^ the Ru-catalyzed isomerization of allylic alcohols,^13^ or the Au-catalyzed hydration of alkynes,^14^ our approach offers access to a range of chiral alcohols and amines starting from nonactivated starting materials (Scheme 1).

## Results and Discussion

### Synthesis and Characterization of CoN_4_SA-POase

**Figure 1:**
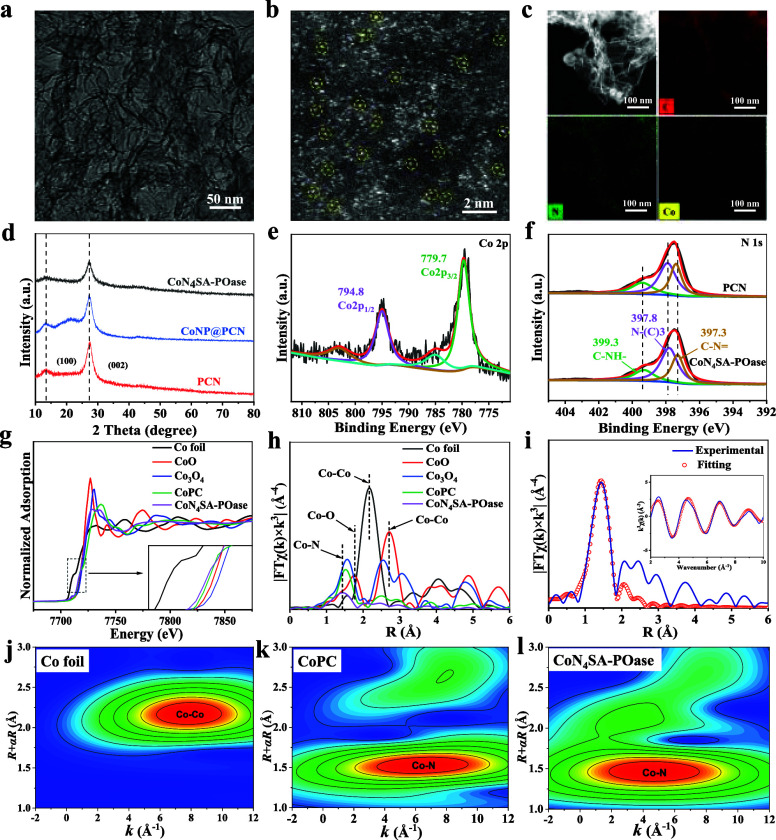
(a) TEM image of CoN_4_SA-POase. (b) AC-STEM-ADF image of CoN_4_SA-POase. (c) HAADF image and the corresponding EDS mapping of CoN_4_SA-POase. (d) XRD patterns of PCN, CoNP@PCN, and CoN_4_-POase. (e) Co 2p XPS spectra of CoN_4_SA-POase. (f) N 1s XPS spectra of PCN and CoN_4_SA-POase. (g) XANES spectra and (h) FT-EXAFS at the Co K-edge of Co foil, CoO, Co_3_O_4_, CoPc and CoN_4_SA-POase each. (i) EXAFS curve fitting of Co SACs at R space. The inset of 2d is a *k* space. Wavelet transforms (WTs) of (j) Co foil, (k) CoPC, and (l) CoN_4_SA-POase.

Thermal copolymerization of polymeric carbon nitrogen (PCN) with cobalt precursors was applied for the fabrication of CoN_4_SA-POases,^15^ in which molecular CoPc was selected as the Co source due to its (1) high chemical and thermal stability and (2) stable CoN_4_ coordination structure preventing cobalt agglomeration in the thermal annealing process. As shown in Figure S1, the Pc ligand was entirely decomposed during the calcination process at 655 °C; therefore, its structure was not present in the final catalyst. The maximum Co loading of 2.3 wt % was achieved at a CoPc/PCN mass ratio of 3%. Co nanoparticles (CoNPs) supported on PCN were also synthesized (CoNP@PCN). Compared to PCN, the morphology of CoN_4_SA-POase is transformed into a twisted and curled structure due to the Co–N chelation interaction that inhibited the expansion of tri-s-triazine units (Figure S1a,b).^16^ Type IV N_2_ adsorption–desorption isotherms were determined for CoN_4_SA-POases (Figure S2), suggesting a microporous structure. As shown in Figure 1a (and Figure S1c), CoNPs were observed in the case of CoNP@PCN but not in the case of CoN_4_SA-POase. Aberration-corrected scanning transmission electron microscopy (AC-STEM)-annular dark-field (ADF) provides solid evidence that individual Co atoms (highlighted by yellow circles) are randomly dispersed on PCN (Figure 1b). EDS elemental mapping also demonstrates the uniform distribution of C, N, and Co throughout the CoN_4_SA-POase (Figure 1c). In the XRD patterns (Figure 1d), only the diffraction peaks (2θ = 13.2 and 27.5°) belonging to PCN are observed, with no detection of signals for cobalt species, also suggesting the high dispersion of Co atoms. The Co 2p spectrum in X-ray photoelectron spectroscopy (XPS) displays that the Co species in CoN_4_SA-POase are composed of Co^2+^ around 780 eV (Figure 1e). In the N 1s spectrum, the peak at 400.3 eV can be assigned to surface amino (N1, C–N–H) groups, while the peaks at 397.5 and 398.6 eV are assigned to the two-coordinated N2 (C–N=C) and tri-coordinated N3 (N–(C)_3_), respectively (Figure 1f).^17^ It is worth noting that the ratios of N1/N3 change from 0.44 in pure PCN to 0.37 in CoN_4_SA-POase and those of N2/N3 change from 0.67 in pure PCN to 0.59 in CoN_4_SA-POase, suggesting that Co should bind with both the N1 and N2 atoms.

The Co K-edge X-ray absorption near-edge structure (XANES) spectra exhibit that the absorption-edge position of CoN_4_SA-POase is close to that of CoPC and lies between the CoO and Co foil (Figure 1g), suggesting that the valence state of Co is between 0 and 2+, and around 2+, which is in line with the XPS results. A Co–N peak at ∼1.5 Å appeared in the Fourier transformed *k*^3^-weighted χ^(*k*)^ function of CoN_4_SA-POase (Figure 1h), which is close to the Co–N covalent bond peak at 1.6 Å of CoPC, demonstrating the formation of the Co–N bond in CoN_4_SA-POase. No obvious Co–Co bond peak at 2.2 Å was found in CoN_4_ SA-POase, further verifying the atomically dispersed Co species. The WT intensity maximum of CoN_4_SA-POase located at 1.5 Å for R space and ∼4.5 Å^–1^ for k space (Figure 1j–l), which can be attributed to the first coordination shell of the Co–N bond.^18^ The quantitative structural parameters of Co sites in CoN_4_SA-POase were obtained from EXAFS curve fitting (Figure S3 and Table S1), which revealed that the coordination number of center Co atoms is about 3.9 and the average bond length of Co–N bonds is 1.9 Å.

Based on these results, an atomic structure model of CoN_4_SA-POase was constructed and subsequently optimized by DFT calculations (Figure S3). The optimized geometry exhibits two distinct features, including the Co–N1 coordination mode and the shorter Co–N bond length. For single-atom Co catalysts, Co–N2 coordination is common, while the Co–N1 coordination mode is very rare (Table S2). The Co center of the Co–N1 coordination is more electron-rich due to the lower electronegativity of N1 than that of N2, making it easier to coordinate with the electron-deficient oxidant and thus activate the oxidant. On the other hand, the distances between the cobalt center and four-coordinated nitrogen atoms are 1.86, 1.90, 1.86, and 1.90 Å, which are among the shortest values for the Co–N bonds in SACs reported thus far (Table S2), an indication of the robust CoN_4_ structure, thus stabilizing the Co atoms.

### Catalytic Activity Investigation of CoN_4_SA-POase

To evaluate the oxyfunctionalization activity of CoN_4_SA-POase, we used ethylbenzene as the model substrate (Table 1). Pleasingly, using *tert*-butyl hydroperoxide (TBHP, 70% aqueous solution, 5 equiv), ethylbenzene was converted at 73% conversion with high (94%) selectivity for acetophenone (**2a**). The only side products observed were the initial hydroxylation product (phenyl ethanol, **4a**, 5.4%) and some traces of benzaldehyde (**3a**). Interestingly, H_2_O_2_ did not enable significant conversion of ethylbenzene. As expected, CoN_4_SA-POase demonstrates a much higher catalytic activity than CoNP@ (Table 1, entries 3 and 4). Increasing the reaction temperature from RT to 40 °C resulted in almost complete conversion into the desired acetophenone (96% conversion and 99% selectivity). Increasing the TBHP dosage increased both the conversion and selectivity (Table 1, entry 9). 85% conversion and 95% selectivity were also obtained under a nitrogen atmosphere, confirming the key role of TBHP rather than O_2_ in the oxidation process (Table 1, entry 10). Gratefully, also in the presence of elevated (400 mM) substrate concentrations, high conversion (85%) and selectivity (96%) were retained. At this juncture, the turnover number (TON) and turnover frequency values were up to 872 and 58 h^–1^, respectively, being among the highest values for the SAC-catalyzed C–H oxidation reported thus far (Table S3).

**Table 1:**
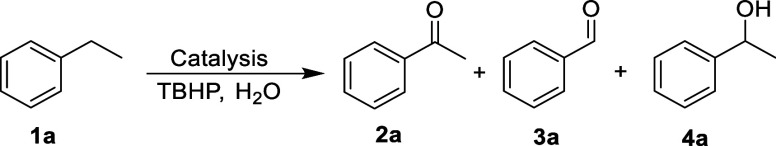
Catalytic Performance of CoN_4_SA-POase in **1a** Oxidation^a^

entry	catalysts	TBHP/1a (n/n)	temp (°C)	conv. (%)^b^	selectivity (%)^b^		
					2a	3a	4a
1	None	5	RT	/	/	/	/
2	PCN	5	RT	15	65	9.7	25
3	CoNP@PCN	5	RT	28	63	7.2	30
4	CoN_4_SA-POase	5	RT	73	94	0.9	5.4
5	CoN_4_SA-POase	5	40	96	99	0.6	0.5
6	CoN_4_SA-POase	5	50	96	99	0.6	0.3
7	CoN_4_SA-POase	3	40	84	92	1.0	7.5
8	CoN_4_SA-POase	4	40	89	94	0.8	5.8
9	CoN_4_SA-POase	6	40	97	99	0.6	0.4
10	CoN_4_SA-POase^c^	5	RT	85	95	0.9	4.2
11	CoN_4_SA-POase^d^	5	40	85	96	1.2	2.9

a Reaction conditions: CoN_4_SA-POase (10.0 mg), **1a** (0.5 mmol, 50 mM), RT-50 °C, 15 h, TBHP (3–6 equiv), 10 mL of H_2_O.

b The conversion and selectivity were determined by GC analysis with dodecane as an internal standard.

c Reaction under a N_2_ atmosphere.

d **1a** (4 mmol, 400 mM).

The stability and reusability of CoN_4_SA-POase in the oxidation of **1a** were investigated under optimized reaction conditions. As shown in Figure S5, CoN4SA-POase was recyclable at least eight times prior to showing indications for decreasing activity and selectivity. ICP-AES results showed that the Co loadings of the collected catalyst after 10 cycles did not display an obvious decrease compared to that of the fresh one (2.05% vs 2.14%). In addition, HRTEM and XRD results of the recycled CoN_4_SA-POase revealed that no Co or CoO nanoparticles were formed (Figures S5 and S6). The excellent stability of CoN_4_SA-POase might be attributed to its stronger CoN_4_ coordination structure, which could trap the migrating Co atoms and further avoid their agglomeration during the catalytic process. Considering the retention of the morphology and structure of the recycled catalyst, the reduction of the catalytic performance after eight reuses may be linked to the loss of catalyst mass following centrifugation after each cycle.

### Catalytic Mechanism Investigation of CoN_4_SA-POase

As shown in the time course of the CoN_4_SA-POase-catalyzed oxyfunctionalization of ethylbenzene (Figure S7), the double oxidation product acetophenone (**2a**) was always the dominating product. The intermediate phenyl ethanol (**4a**) never accumulated to >5 mM and was depleted at prolonged reaction times. One obvious explanation for this kinetic behavior is that phenyl ethanol, as a significantly more activated starting material, is converted at much higher rates than ethylbenzene. This assumption, however, does not explain the observation of trace amounts of benzaldehyde. DMPO (5,5-dimethyl-1-pyrroline N-oxide) spin-trapping electron paramagnetic resonance experiments (Figure S8) revealed the presence ·OH, *t*-BuOO· and some other alkyl radicals,^7a^ suggesting a free radical mechanism for the *t*-BuOOH-driven and CoN_4_SA-POase-catalyzed oxyfunctionalization of ethylbenzene.

**Figure 2:**
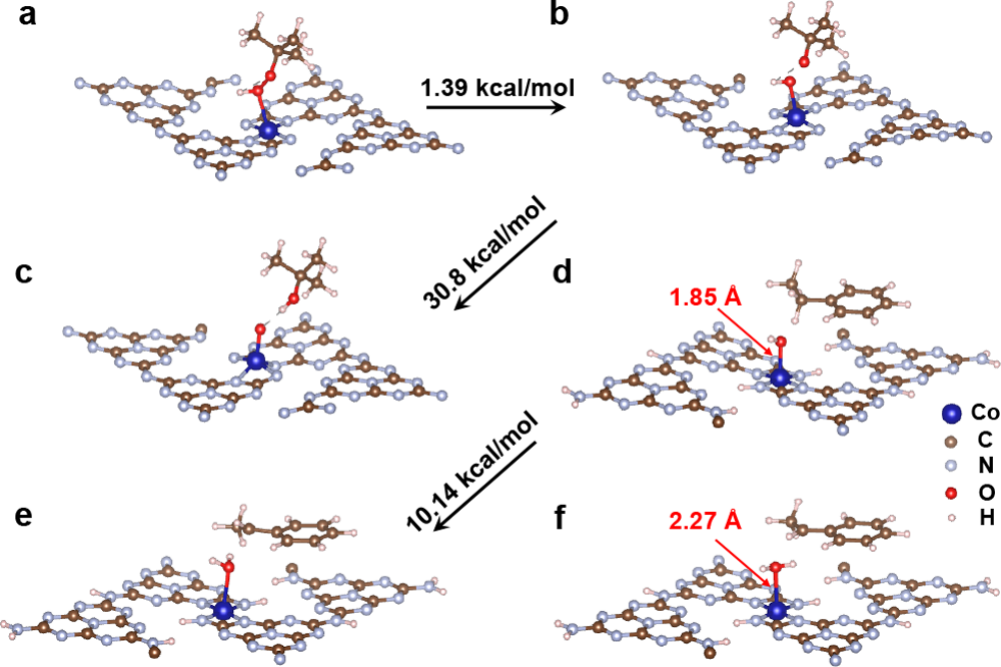
DFT calculations of **1a** oxidation on CoN_4_SA-POase. (a, b) Initial and final states of the O–O bond homolytic cleavage process of TBHP. (c) State of hydrogen capture of the *t*-BuO· radical and formation of Co=O. (d–f) Initial, transition, and final states in the step of **1a** activation.

These observations were also supported by DFT calculations (Figure 2). Accordingly, TBHP coordinates to the Co(II) center via its distal O-atom, thereby stretching the O–O bond from 1.42 to 1.50 Å, facilitating homolytic cleavage of the O–O bond. As a result, the Co(II)-hydroxy active species as well as a *tert*-butyloxy radical (*t*-BuO·) are formed. This represents the energetically most favorable reaction, with only 1.39 kcal/mol of bond dissociation energy being required. An alternative high-valent metal Co=O compound (analogous to CpdI in heme-dependent oxygenases) originating from H-atom abstraction from the Co(I)-hydroxy species by *t*-BuO· (Figure 2, b–c) was predicted but with a very high bond dissociation energy (30.8 kcal/mol). It is interesting to note that in the case of CoNPs, both *t*-BuO· and ·OH radicals adsorbed to the Co (0001) surface,^7b^ explaining why CoN_4_SA-POase shows a higher catalytic activity than CoNP@PCN.

As shown in Figure 2d, ethylbenzene binds to the catalyst, presenting a benzylic hydrogen atom to the Co(II)-hydroxy species in CoN_4_SA-POase. The transition-state energy barrier for a H-abstraction step (Figure 2e) is as low as 10.14 kcal mol^–1^. In contrast, the generation of an α-ethylbenzene radical from a Co=O species requires a higher energy barrier of 26.2 kcal mol^–1^. As shown in Figure 2d,f, the distance between cobalt and oxygen in H_2_O@CoN_4_SA-POase is 2.17 Å, which is longer than that in ·OH@CoN_4_SA-POase (1.85 Å). The calculated desorption energy for H_2_O@CoN_4_SA-POase is only 5.10 kcal mol^–1^, indicating that the water molecule can be easily desorbed; thus, Co poisoning seems unlikely.

**Figure 3:**
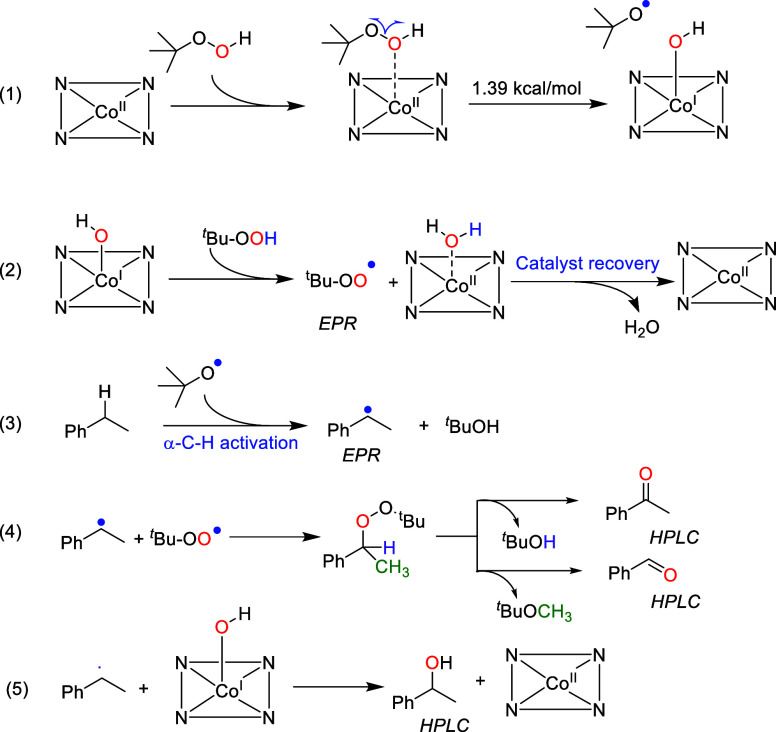
Proposed reaction mechanism of ethylbenzene oxidation with CoN_4_SA-POase.

Based on the above results and the results reported by Zhang and co-workers,^19^ we proposed a possible pathway for ethylbenzene oxidation over CoN_4_SA-POase with TBHP (Figure 3). In a first step, the catalytically active Co(I)-hydroxy active species and t-BuO· are formed via homolytic O–O bond cleavage (eq 1). The Co(I)-hydroxy species then abstracts a H-atom of the TBHP to produce t-BuOO·, with the formation of a water molecule, which was easily desorbed to achieve catalyst recovery (eq 2). The as-formed t-BuO· (eq 1) performs a H-atom abstraction of the benzylic position of the starting material, yielding the benzylic radical (eq 3). For the actual oxyfunctionalization step, we propose a radical combination between the benzylic radical and a t-BuOO· radical, forming an intermediate peroxyether (eq 4). The latter degrades via migration of either the hydrogen or methyl substituent of the benzylic carbon atom (eq 4). Thus, compared to H, the very low migrational tendency of the CH_3_ group also explains the observation of trace amounts of benzaldehyde. Finally, for the formation of 1-phenyl ethanol, a direct interaction of the benzyl radical with the Co(I)-hydroxy species may be hypothesized (eq 5).

### Construction of Continuous-Flow Systems

Compared to batch reactions, flow systems are able to increase productivity by running continuously over extended durations, simplify downstream processing by utilizing packed-bed reactors, and improve process compatibility by performing incompatible reaction modules separately in different reactors. In this context, we transitioned the artificial catalyst (CoN_4_SA-POase) from a batch to a flow system and subsequently investigated its combination with enzymes for continuous-flow chemoenzymatic cascades. The flow system was constructed as shown in Table 2, in which the involved solid catalyst was filled in a PBR (4.6 × 200 mm). The molar ratio of TBHP/**1a**, substrate concentration, and flow rate were optimized to achieve maximum productivity. A molar excess of TBHP over **1a** of 10 was needed to attain full conversion of the ethylbenzene starting material. Possibly, the CoN_4_SA-POase-catalyzed dismutation of TBHP to O_2_ and ^tert^Butanol may account for this. Flow rate is a key parameter affecting conversion and space-time yield (STY) in flow systems. As shown in Table 2, entries 11–13, the faster the flow rate, the lower the conversion due to the shorter residence time, whereas the case of STY is more complicated as it is determined by both flow rate and residence time. A flow rate of 0.08 mL/min led to the highest STY of 40.1 g L^–1^ h^–1^ at a substrate concentration of 300 mM, which was 17-fold higher than that of the batch system (2.4 g L^–1^ h^–1^ at a substrate concentration of 400 mM). Finally, the operational stability of the flow system was investigated by continuous ethylbenzene oxidation. As shown in Figure S9, after 72 h of continuous operation, the flow system maintained about 90% of the initial activity, from which a half-life time of approximately 400 h was estimated, demonstrating excellent potential for practical applications.

**Table 2:**
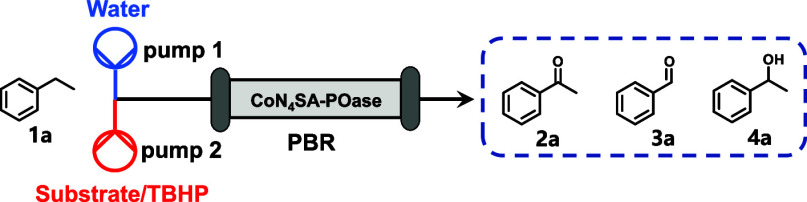
Optimization of the Continuous-Flow System for C–H Oxidation^a^

entry	flow rate (mL/min)^b^	TBHP/1a	1a (mM)^c^	conv. (%)^d^	sel. (%)^d^	STY (g L^–1^ h^–1^)
1	0.04	5	100	26	85	1.92
2	0.04	8	100	60	88	4.58
3	0.04	10	100	99	99	8.50
4	0.04	12	100	99	99	8.50
5	0.04	10	200	99	99	17.0
6	0.06	10	200	98	99	25.2
7	0.08	10	200	94	97	31.6
8	0.04	10	300	97	99	25.0
9	0.06	10	300	90	96	33.7
10	0.08	10	300	81	95	40.1
11	0.04	10	400	76	92	24.3
12	0.06	10	400	53	91	25.1
13	0.08	10	400	40	87	24.2

a Aqueous phase: deionized water, oil phase: **1a** in TBHP.

b The flow rate is the total flow rate of P1 and P2.

c The concentration here is the final concentration of **1a** after mixing the two phases of water and oil to correspond to the batch reaction.

d The conversion and selectivity of **2a** were determined by GC analysis with dodecane as an internal standard.

**Figure 4:**
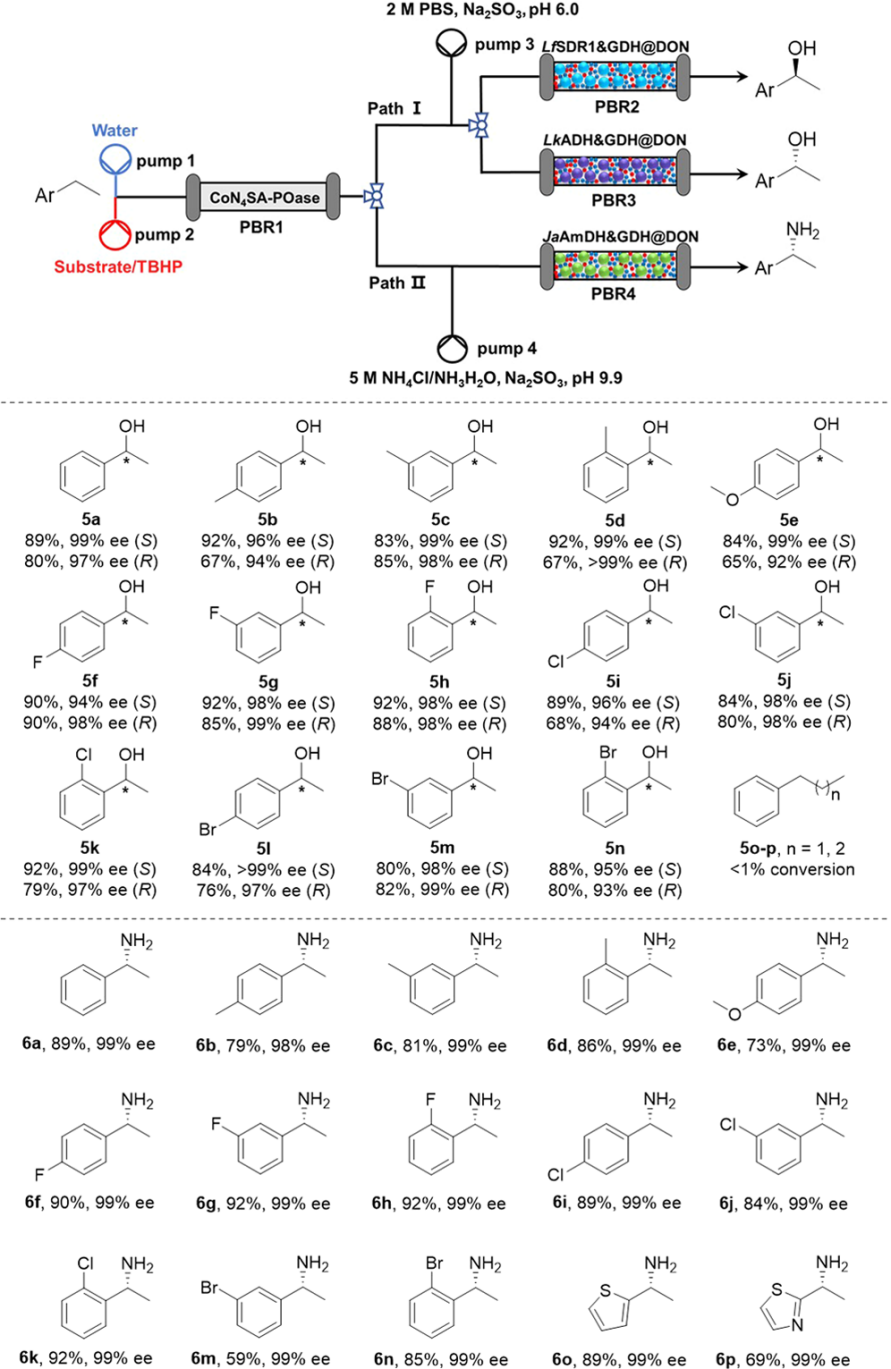
Continuous-flow artificial peroxygenase-natural enzyme cascades for enantioselective C–H functionalization. P1 (0.042 mL/min): deionized water; P2 (0.018 mL/min): a mixed solution of substrate (300 mM) and TBHP (10 equiv); P3 (0.009 mL/min): PBS (pH 6.0, 2 M) containing Na_2_SO_3_ (1 M), glucose (1.5 M), and NADP^+^ (20 mM); P4 (0.009 mL/min): NH_4_CI/NH_4_OH (pH 9.9, 5 M) containing Na_2_SO_3_ (1 M), glucose (1.5 M), and NAD^+^ (20 mM); PBR1 (4.6 × 200 mm): CoN_4_SA-POase (100 mg) and silica gel powder (1 g); PBR2 (4.6 × 100 mm): *Lf*SDR1&GDH@DON (500 mg); PBR3 (4.6 × 150 mm): *Lk*ADH&GDH@DON (750 mg); PBR 4 (4.6 × 150 mm): *Ja*AmDH&GDH@DON (750 mg); reaction temperature: 40 °C.

Next, we aimed to establish enantioselective reduction of the acetophenone product. For this, we chose the (*S*)-selective ketoreductase from *Lactobacillus fermentum* (*Lf*SDR1)^20^ and the (*R*)-selective *LK*ADH from *Lactobacillus kefir*.^21^ The individual enzymes were coimmobilized with glucose dehydrogenase (GDH, for in situ cofactor regeneration) on dendritic organosilica nanoparticles (DONs) by a continuous-flow immobilization method.^22^ Specifically, a solution containing *Lf*SDR1 (or *LK*ADH) and GDH is pumped into a PBR filled with activated DONs, resulting in the in situ covalent attachment of the enzymes to DONs (for details, see the Supporting Information), yielding *Lf*SDR1&GDH@DON and *LK*ADH&GDH@DON (125 and 132 mg_protein_/g_support_, respectively). The resultant bio-PBRs were added in sequence after the chemocatalytic step. In order to avoid possible issues of enzyme inactivation by the remaining TBHP, it was quenched by the addition of Na_2_SO_3_ (Figure 4).

Notably, due to the lower activity of *LK*ADH than that of *Lf*SDR1, a longer PBR was used for *LK*ADH to achieve comparable productivity. This way, the overall stereoselective hydroxylation of a broad range of ethylbenzene derivates was realized. The enantiocomplementary enantioselective C–H hydroxylation was achieved in high yields and enantioselectivity (Figure 4, **5a**–**n**), with the maximum STYs for the synthesis of (*S*)- and (*R*)-1-phenyl ethanol of 5.9 and 4.5 g L^–1^ h^–1^, respectively. However, CoN_4_SA-POase showed a very low catalytic activity toward the bulky propylbenzene (**5o**) and butylbenzene (**5p**), with <1% conversion after 15 h reaction.

Finally, we constructed an artificial peroxygenase-natural enzyme cascade for continuous-flow enantioselective C–H amination. For this, the amine dehydrogenase from *Jeotgalicoccus aerolatus* (*Ja*AmDH)^23^ and GDH were coimmobilized, obtaining *Ja*AmDH&GDH@DON (98 mg_protein_/g_support_). Then, it was combined with CoN_4_SA-POase in the continuous-flow system, producing the corresponding chiral (*R*)-amines in 59–92% yields and 99% ee (Figure 4, **6a**–**o**), with an STY for the synthesis of, e.g., (*R*)-1-phenyl ethylamine (**6a**) up to 10.0 g L^–1^ h^–1^. As shown in Figure 4, the system could be quickly switched between the different flow paths without intermittent washing steps, thus allowing the selective synthesis of a specific class of products or the simultaneous generation of several classes of products. Under optimized conditions, a 72 h continuous production was performed, furnishing 4.54 g of **5a** and 2.42 g of **6a**, which demonstrated the synthetic usefulness of the chemoenzymatic flow system.

## Conclusions

In conclusion, we fabricated an artificial peroxygenase (CoN_4_SA-POase) with CoN_4_ active sites by supporting single-atom cobalt on polymeric carbonitride. The CoN_4_SA-POase was demonstrated as a promising heterogeneous catalyst for the oxyfunctionalization of a range of benzylic CH_2_ groups to the corresponding acetophenone derivates. Based on experimental results and DFT calculations, we have proposed a catalytic mechanism. Finally, the combination of enantioselective alcohol dehydrogenases and amine dehydrogenase enabled the synthesis of optically pure benzyl alcohols and amines in a simple, modular flow system. We are convinced that this chemoenzymatic approach bears significant potential for the preparative synthesis of chiral alcohols and amines from simple starting materials.

## Methods

### Preparation of CoN_4_SA-POase

First, citric acid (1 g) was added to a mixed solution of 2-propanol and acetone (2:1, v/v, 30 mL). After the mixture was stirred for 10 min, a transparent solution was obtained, followed by the addition of CoPc (30 mg). The violet solution was stirred for 2 h, and the as-formed PCN powder (1 g) was added. The mixture was stirred and naturally evaporated to a 10 mL volume. Then, the whole mixture was transferred into an agate mortar and ground to a dry powder. The yellow powder was heated to 655 °C at a ramp rate of 7 °C min^-1^ and kept for 2 h under an argon atmosphere at a flow rate of 50 mL min^–1^. Finally, the resulting solid (CoN_4_SA-POase) was ground to powder for later use.

### General Procedure for the Chemoenzymatic Cascades

Chemical step: The reaction was conducted in a two-necked flask equipped with a 20 mL pressure-equalizing dropping funnel, filled with 10 mg of catalyst and substrate (0.5 mmol). Then, TBHP (2.5 mmol, 70 wt % in water) and water (10 mL) were filled in the funnel and added dropwise into the flask over 30 min at room temperature. After that, the reaction mixture was stirred at 40 °C for 15 h. After the reaction, the solid catalyst was recovered by filtration and the supernatant was transferred without purification to a round-bottom flask for enzyme catalysis.

Enzymatic step: Initially, Na_2_SO_3_ was added to the above-obtained solution to quench the oxidant (TBHP). Then, the corresponding buffer solution containing enzymes (*LK*ADH, *Lf*SDR1, or *Ja*AmDH), cofactor (NAD^+^ or NADP^+^), GDH, and glucose was added (for more details, see the Supporting Information). The mixture was stirred at 30 °C until the reaction was completed. The reaction solution was extracted with Et_2_O (5 mL × 3), and the organic phase was dried using anhydrous Na_2_SO_4_. The solvent was concentrated in vacuo to obtain the crude products, which were purified by column chromatography.

## Supporting Information Available

Materials and methods, optimization details, general catalytic procedure, figures, tables, and analytical data (DOCX)
