# Supplementary material for: A Chemoenzymatic Cascade for the Formal Enantioselective Hydroxylation and Amination of Benzylic C–H Bonds
Source: ACS Catal. 2024 Nov 12;14(23):17405–12. doi: 10.1021/acscatal.4c03161 (PMC11629291; doi:10.1021/acscatal.4c03161)
Supplement: Supplementary file 1 — cs4c03161_si_001.docx [file cs4c03161_si_001.docx]

**Supporting Information**

**A chemoenzymatic cascade for the formal enantioselective hydroxylation and amination of benzylic C-H bonds**

Yuqing Zhang,^[a]^ Chen Huang,^[a]^ Weixi Kong,^[a]^ Liya Zhou,^[a]^ Jing Gao,^[a]^ Frank Hollmann,*^[b]^ Yunting Liu*^[a]^ and Yanjun Jiang*^[a]^

[a] School of Chemical Engineering and Technology, Hebei University of Technology, Tianjin 300401, China.

[b] Department of Biotechnology, Delft University of Technology

2629 HZ Delft, The Netherlands.

*E-mail: f.hollmann@tudelft.nl;

*E-mail: ytliu@hebut.edu.cn;

*E-mail: yanjunjiang@hebut.edu.cn

**Contents**

[Chemicals and materials 4](#_Toc27243)

[Materials and analytical methods 5](#_Toc23000)

[Protein expression and purification^[1]^ 6](#_Toc4357)

[Preparation of PCN 7](#_Toc23059)

[Preparation of CoN_4_SA-POase 7](#_Toc30293)

[Preparation of CoNP@PCN 7](#_Toc12735)

[Preparation of the solid support materials and enzyme immobilization^[3]^ 8](#_Toc11169)

[General procedures for synthesizing racemic alcohols^[4]^ 10](#_Toc25479)

[General procedures for synthesizing racemic amines^[5]^ 10](#_Toc3427)

[General procedure for C-H bond oxidation catalyzed by CoN_4_SA-POase in batch 11](#_Toc5264)

[General procedure for bienzymatic DARA in batch 11](#_Toc17526)

[Continuous flow equipment 13](#_Toc10609)

[Continuous oxidation of ethylbenzene to acetophenone in PBRs 14](#_Toc3504)

[Continuous operational stability of the CoN_4_SA-POase 14](#_Toc5073)

[Continuous-flow artificial peroxygenase-natural enzyme cascades for enantioselective C-H functionalization in PBRs 15](#_Toc25474)

[NMR and GC data 16](#_Toc7716)

[Characterization 26](#_Toc16716)

[XAFS measurements and analysis details 26](#_Toc11805)

[Computational details 27](#_Toc13951)

[Calculation formulas 28](#_Toc31688)

[Figure S1. FTIR spectra of CoPc and CoN_4_SA-POase. 29](#_Toc28627)

[Figures 30](#_Toc27246)

[Figure S2. SEM images of (a) PCN and (b) CoN_4_SA-POase. (c) TEM image of CoNP@PCN 30](#_Toc20243)

[Figure S3. N_2_ absorption and desorption curves of PCN, CoNP@PCN and CoN_4_SA-POase. 31](#_Toc22967)

[Figure S4. The structure model of CoN_4_SA-POase. 32](#_Toc11845)

[Figure S5. Reusability of the CoN_4_SA-POase catalyst for catalyzing 1a oxidation. 33](#_Toc14492)

[Figure S6. TEM of CoN_4_SA-POase after 10 reuse times. 34](#_Toc4423)

[Figure S6. XRD patterns of CoN_4_SA-POase before use and after 10th reuse. 35](#_Toc12583)

[Figure S8. Reaction time curves of CoN_4_SA-POase-catalyzed oxidation of 1a. 36](#_Toc24849)

[Figure S9. DMPO (5,5-dimethyl-1-pyrroline N-oxide) spin-trapping EPR experiment. The spectrum was recorded after 1 min upon introducing DMPO to the reaction system. Ethylbenzene was used as the substrate. 37](#_Toc15136)

[Figure S10. Operational stability of the flow system in the continuous synthesis of 2a. 38](#_Toc26170)

[Tables 39](#_Toc5386)

[Table S1. EXAFS fitting parameters at the Co K–edge for various samples. 39](#_Toc9331)

[Table S2. Comparison of Co-N coordination modes. 40](#_Toc24094)

[Table S3. Comparison of ethylbenzene oxidation by different catalytic systems 41](#_Toc1815)

[NMR spectra 42](#_Toc8498)

[GC spectraGC traces 71](#_Toc3840)

[Reference 101](#_Toc24483)

# Chemicals and materials

All chemicals and reagents were purchased from J & K, Acros, Aldrich, and Aladdin, and chemicals were obtained from authentic suppliers of at least of reagent grade and used without further purification. Nicotinamide adenine dinucleotide (NADH) and Nicotinamide adenine dinucleotide phosphate oxidase (NADPH) was purchased from Aladdin (Shanghai, China).

# Materials and analytical methods

For thin-layer chromatography (TLC) analysis throughout this work, Merck precoated TLC plates (silica gel 60 GF254, 0.25 mm) were used. The products were purified by preparative column chromatography on silica gel E. Merck 9385. NMR spectra were recorded on a Bruker AV 400 spectrometer at 400 MHz ^1^H NMR and 101 MHz ^13^C NMR. Chemical shifts were reported in ppm relative to internal TMS for ^1^H NMR data, respectively. Data are presented in the following space: chemical shift, multiplicity, coupling constant in hertz (Hz), and signal area integration in natural numbers. The conversion and enantiomeric excess were determined by GC analysis on a Shimadzu-2010 gas chromatograph with a flame ionization detector (FID) using nitrogen as carrier gas. Agilent J&W CP-Chiralsil-DEX CB capillary column (25 m × 0.25 mm × 0.25 μm) was used for determining the conversions and ee values, respectively.

# Protein expression and purification^[1]^

The gene encoding of the 15 candidate recombinant proteins with the same mutations (K67S/N260L), *Ja*AmDH mutants, *Lf*SDR1, *Lk*ADH, and GDH were inserted into expressing vector pET-28a (+), and the recombinant plasmids were transformed into *E. coli* BL21 (DE3) as the host organism for heterologous expression. Recombinant *E. coli* BL21 (DE3) strain was cultured in 10 mL LB media containing 50 μg/mL kanamycin (final concentration) at 37 °C and 180 rpm overnight. Next, 0.5 mL preculture was inoculated into a shake flask containing 50 mL TB media with 50 μg/mL kanamycin, cultured at 37 °C and 180 rpm until the culture’s optical density (OD_600_) reached 0.6-0.8. The expression of the enzyme was induced by the addition of 0.5 mM IPTG (final concentration) and performed at 24 h at 20 °C. Then, the cells were harvested by centrifugation (5000 g, 4 °C, 10 min) and washed several times with precooling potassium phosphate buffer (50 mM, pH 7.5). The resuspended cells were disrupted by high-pressure homogenization, and then, the lysate was centrifuged at 13000 g for 45 min to remove the cell debris.

Ni-NTA affinity chromatography was performed to purify the filtered lysate. After pre-equilibrating the Ni-NTA column with buffer A (50 mM KH_2_PO_4_, 500 mM NaCl, and 10 mM imidazole at pH 7.5), the filtered lysate was loaded into the Ni-NTA column. Subsequently, bound protein was recovered with gradient elution buffer (50 mM KH_2_PO_4_, 500 mM NaCl, pH 7.5) containing 30 mM, 60 mM, 300 mM, and 500 mM imidazole for 5-10 column volumes, respectively. Fractions containing target protein were verified by SDS-PAGE analysis and concentrated by ultrafiltration.

# Preparation of PCN

The PCN was synthesized by annealing urea in air. First, urea (CON_2_H_4_, 90 g) was put into a covered alumina crucible and placed inside a muffle furnace. Then, the temperature was increased up to 600 °C (ramp rate 10 ^o^C min^-1^) and held for 2 h. Finally, around 7 g of PCN was collected. For safety, the annealing process was carried out in a fume cupboard to ventilate the produced smoke. Before usage, the PCN was further ground for 20 min to get a uniform yellowish powder.

# Preparation of CoN_4_SA-POase

CoN_4_SA-POase (Co single atom doped PCN) was synthesized according to reported work.^[2]^ First, citric acid (C_6_H_8_O_7_, 1 g) was added to a mixed solution of isopropanol and acetone (volume ratio of 2:1, 5 mL). After stirring for 10 min, a transparent solution was obtained followed by the addition of cobalt (II) phthalocyanine (CoPc, 30 mg). The violet solution was stirred for 2 h and the fine ground PCN powder (1 g) was added to the above solution. The mixture was stirred and naturally evaporated to 10 mL volume. Then, the whole mixture was transferred into an agate mortar and ground to dry powder. The yellow powder was heated to 655 ^o^C at a ramp rate of 7 °C min^-1^ and kept for 2 h under Argon atmosphere at a flow rate of 50 mL min^-1^. Finally, the obtained products were grinded to powder and marked as CoN_4_SA-POase.

# Preparation of CoNP@PCN

CoNP@PCN (Co nanoparticles doped PCN) was synthesized according to reported work.^[2]^ Cobalt (II) acetylacetonate (C_10_H_16_CoO_4_, 50 mg) and PCN powder (200 mg) were first ultrasonicated for 10 min and dispersed in 30 mL of ethanol and then transferred into 100 mL of ethylene glycol solution. The dispersion was stirred for 1 h before being placed in a lab-use microwave oven (1000 W) in a fume cupboard and heated for 5 min. The solution was then filtered using a membrane film and washed with ethanol 5 times. The final yellowish powder was dried in an oven at 80 °C for 5 h and was denoted as CoNP@PCN.

# Preparation of the solid support materials and enzyme immobilization^[3]^

**Preparation of DON**

The mixture of aqueous urea solution (0.4 M, 30 g), cetyltrimethylammonium bromide (CTAB, 1 g), *n*-butanol (1 g), and cyclohexane (12 g) was ultrasonicated for 30 min at room temperature. With vigorous stirring, the mixture of Tetraethylortosilicate (TEOS, 1.4 g) and bis-1,2-(triethoxysilyl)ethane (BTSE 0.6 g) was added dropwise to the above solution. After vigorous stirring for 30 min at room temperature, the reaction was continued at 70 °C for another 24 h. The products were centrifuged at 12000 g for 10 min to remove the supernate, and washed with ethanol and water for several times. Finally, the DON was redispersed in 250 mL of acetone and refluxed at 80 °C for 48 h to remove the templates prior to use.

**Modification of DON**

Post-modification method was employed to achieve the amino-functionalization of DON. Briefly, 1.0 g of the DON was added into 100 mL of *n*-hexane, and ultrasonicated for 30 min. Then, 1.0 mL of APTES was added, and the mixture was stirred at 70 °C for another 24 h. The amino-functionalized products (NH_2_@DON) were centrifuged at 12000 g for 10 min to remove the supernate, and washed with ethanol and water for several times, and dried at room temperature overnight. Then, the NH_2_@DON was activated with glutaraldehyde (2 wt%) at room temperature for 2 h. After activation, the DON was washed extensively with phosphate buffer solution (PBS, 100 mM, pH 7.0) for complete removal of unreacted glutaraldehyde and dried at 60 °C overnight.

**Enzyme immobilization**

The glutaraldehyde-activated DON is stored at room temperature. For immobilization, the desired volume of glutaraldehyde-activated DON was first washed with 10 bed volumes of deionized water, followed by 10 bed volumes of binding buffer (50 mM PBS, pH 7.5). With the column equilibrated in binding buffer, enzyme can be introduced to the glutaraldehyde-activated DON in form of purified protein. After incubation overnight on a rotary mixer, the glutaraldehyde-activated DON is washed with 10 bed volumes of binding buffer, followed by 10 bed volumes of 50 mM PBS at pH 7.5. The glutaraldehyde-activated DON is now ready to be packed into a column to immobilized enzymes.

For *Lf*SDR1&GDH@DON: Stock solutions of *Lf*SDR1 (0.4 mg/mL) and GDH (0.8 mg/mL) in binding buffer were prepared and kept at 4 °C or on ice for the duration of the experiment. 500 mg glutaraldehyde-activated DON was packed into the PBR (4.6 × 100 mm) and the column was attached into the flow system. After equilibration of the column with at least 20 mL of binding buffer, the inlet was switched to the enzyme solution, which flowed slowly at 0.3 mL/min through the column. When the protein concentration in the effluent from the PBR outlet remains stable and no longer changes, it indicates that the immobilized enzyme has reached saturation. At this point, the flow should be stopped and the remaining enzyme solution should be recovered.

For the immobilization of *Lk*ADH&GDH@DON, an analogous procedure is employed with modifications to the initial protein concentrations, namely 0.9 mg/mL for *Lk*ADH and 0.3 mg/mL for GDH, as well as the utilization of a PBR (4.6 × 150 mm, packed with 750 mg glutaraldehyde-activated DON) as the reaction vessel.

# General procedures for synthesizing racemic alcohols^[4]^

The corresponding ketone (20 mmol) was dissolved in tetrahydrofuran solution (100 mL), lithium Aluminum Hydride (40 mmol) was added and stirred under argon atmosphere at room temperature for 4 days. The reaction was quenched with NaOH (6M, 100 mL) and the organic phase was separated. The aqueous phase was extracted with tert-butyl methyl ether (3 × 50 mL). The organic phase was dried over Na_2_SO_4_, and the solvent was evaporated under reduced pressure. Purification using silica-gel column chromatography (Mso/EtOAc).

# General procedures for synthesizing racemic amines^[5]^

Ammonium acetate (2.691 g, 35 mmol) and sodium cyanoborohydride (438 mg, 7 mmol) were added to a solution of the corresponding ketone (3.5 mmol) in anhydrous methanol (11 mL, 0.32 M). The reaction was stirred for 24 h at room temperature. After this time the solvent was evaporated under reduced pressure and the remaining residue was dissolved in distilled water (20 mL). The solution was acidified to pH 1 with concentrated HCl and then washed with EtOAc (3 × 20 mL), discarding the organic layer. Then, the aqueous phase was basified to pH 10 with an aqueous NaOH 10 M solution (5 mL) and extracted with CH_2_Cl_2_ (3 × 20 mL). The organic layers were combined, dried over Na_2_SO_4_, filtered and the solvent evaporated under reduced pressure. The crude amine was purified by column chromatography on silica gel (eluent gradient EtOAc/MeOH 99:1 to 90:10).

# General procedure for C-H bond oxidation catalyzed by CoN_4_SA-POase in batch

The reaction was conducted in a two-necked flask equipped with a 20-mL pressure-equalizing dropping funnel, filled with 10 mg catalyst and substrate (0.5 mmol). Then, TBHP (2.5 mmol, 70 wt% in water) and water (10 mL) were filled in the funnel, and added dropwise into the flask over 30 minutes at room temperature. After that, the reaction mixture was stirred at 40 °C for 15 h. After the reaction, the solid catalyst was recovered by filtration, while the filtrate was extracted with ethyl acetate (3 × 10 mL). The products were quantitatively analyzed by using an Agilent CP7503 system. Dodecane was used as an internal standard.

# General procedure for bienzymatic DARA in batch

The corresponding ketones (50 mM) was dissolved in 5 mL PBS buffer (100 mM, pH = 6) containing 20 mg *Lf*DSR1&GDH@DON, NADP^+^ (2.5 mM), glucose (75 mM, 1.5 equiv.). The reaction mixture was shaken vigorously at a 30 °C incubator for 6 h. After the completion of the reaction, the solution was extracted with ethyl acetate (3 × 10 mL). The organic phase containing the product was then dried by Na_2_SO_4_, and concentrated under reduced pressure.

The corresponding ketones (50 mM) was dissolved in 5 mL PBS buffer (100 mM, pH = 6) containing 100 mg *LK*ADH&GDH@DON, NADP^+^ (2.5 mM), glucose (75 mM, 1.5 equiv.). The reaction mixture was shaken vigorously at a 30 °C incubator for 6 h. After the completion of the reaction, the solution was extracted with ethyl acetate (3 × 10 mL). The organic phase containing the product was then dried by Na_2_SO_4_, and concentrated under reduced pressure.

The corresponding ketones (50 mM) was dissolved in 5 mL NH_4_Cl/NH_3_H_2_O buffer (2 M, pH = 9.5) containing 100 mg *Ja*AmDH&GDH@DON, NAD^+^ (2.5 mM), glucose (75 mM, 1.5 equiv.). The reaction mixture was shaken vigorously at a 30 °C incubator for 12 h. After the completion of the reaction, the solution was extracted with ethyl acetate (3 × 10 mL). The organic phase containing the product was then dried by Na_2_SO_4_, and concentrated under reduced pressure.

# Continuous flow equipment

Continuous flow reactions were performed using the following equipment: High pressure infusion pumps (AP0010, flow rate 0.001-10.000 mL min^-1^) was purchased from Sanotac (Shanghai, China). High pressure infusion pumps (P3700, flow rate 0.001-10.000 mL min^-1^) was purchased from Eclassical (Dalian, China). Each module was equipped with a 250 mL reservoir bottle. Stainless steel (1/16 outer diameter, 0.75 mm inner diameter) and tee junctions were purchased from Zhijia (Hangzhou, China).

# Continuous oxidation of ethylbenzene to acetophenone in PBRs

Optimized conditions: the reaction mixture was made into two separate solutions, solution one: deionized water, pumped through P1 (0.042 mL/min); solution two: **1a** (300 mM) in TBHP (the concentration here is the final concentration of aromatics after mixing the two phases of water and oil to correspond to the batch reaction, and the amount of TBHP is 10 equiv. of the **1a**), pumped through P2 (0.018 mL/min). The substrate solution was kept at room temperature. The CoN_4_SA-POase (100 mg) and silica gel powder (1 g) were mixed and packed into the liquid chromatography empty column tube (PBR, 4.6 × 200 mm) and the column temperature was maintained at 40 ^o^C under oil bath. The two-phase system passed into the pipeline (1/16 outer diameter, 0.75 mm inner diameter), mixed and then entered the PBR. The mixed solution then flowed out from the PBR and the effluent was processed for testing.

# Continuous operational stability of the CoN_4_SA-POase

The substrate tank was filled with a sufficient amount of substrate and solvent. The operation stability of the CoN_4_SA-POase was determined by continuous running the flow system for a long period of time (72 h) by monitoring the AcPO yield and selectivity each 8 hours. The yield observed at the first 8 h moment was assumed to be 100% activity. Effluent for every 8 h operation was collected and processed for testing.

# Continuous-flow artificial peroxygenase-natural enzyme cascades for enantioselective C-H functionalization in PBRs

Optimized conditions: the initial reaction mixture was made into two separate solutions, solution one: deionized water, pumped through P1 (0.042 mL/min); solution two: substrate (300 mM) in TBHP (the concentration here is the final concentration of substrate after mixing the two phases of water and oil to correspond to the batch reaction, and the amount of TBHP is 10 equiv. of the substrate), pumped through P2 (0.018 mL/min). The substrate solution was kept at room temperature. The CoN_4_SA-POase (100 mg) and silica gel powder (1 g) were packed into the first liquid chromatography empty column tube (PBR1, 4.6 × 200 mm) and the column temperature was maintained at 40 °C under oil bath. The effluent flowed through the first tee junction, shunt to Path I (chiral alcohols) and Path II (chiral amines). Path I: P3 (0.009 mL/min) was then pumped the *Lf*SDR1/*Lk*ADH substrate reservoir (2 M PBS, pH 6.0, containing 1 M of Na_2_SO_3_, 1.5 M of glucose, 20 mM of NADP^+^) and mixed with effluent from the PBR1. The mixture flowed through the second tee junction into PBR2 (4.6 × 100 mm, filled with about 500 mg *Lf*SDR1&GDH@DON) and PBR3 (4.6 × 150 mm, filled with about 750 mg *Lk*ADH&GDH@DON), respectively. Path II: P4 (0.009 mL/min) was then pumped the *Ja*AmDH substrate reservoir (5 M NH_4_Cl/NH_3_H_2_O, pH 9.9, containing 1 M of Na_2_SO_3_, 1.5 M of glucose, 20 mM of NAD^+^) and mixed with effluent from the PBR1. The mixture flowed into PBR4 (4.6 × 150 mm, filled with about 750 mg *Ja*AmDH&GDH@DON). The final effluent was processed for testing.

# NMR and GC data

^1^H NMR (400 MHz, Chloroform-d) δ 7.45 – 7.30 (m, 5H), 4.92 (q, J = 6.3 Hz, 1H), 2.24 (s, 1H), 1.53 (d, J = 6.5 Hz, 3H). ^13^C NMR (101 MHz, Chloroform-d) δ 145.91, 128.56, 127.52, 125.47, 70.44, 25.20. GC conditions: Agilgent CP-Chirasil Dex CB (df = 0.25 μm, 0.32 mm i.d. × 25 m); carrier gas, N_2_ (flow 30 mL/min); injection temp, 280 °C; initial column temperature 100 °C, then progress rate, 5 °C/min; final column temperature, 200 °C for 5 min.

(*S*)-**5a**, 99% ee, t*_R_* = 5.787 min (minor) and t*_S_* = 5.964 min (major).

(*R*)-**5a**, 97% ee, t*_R_* = 5.724 min (major) and t*_S_* = 6.609 min (minor).

1H NMR (400 MHz, Chloroform-d) δ 7.30 (d, J = 7.6 Hz, 2H), 7.20 (d, J = 7.6 Hz, 2H), 4.89 (q, J = 6.1 Hz, 1H), 2.39 (s, 3H), 2.04 (s, 1H), 1.59 – 1.45 (m, 3H). 13C NMR (101 MHz, Chloroform-d) δ 137.18, 129.22, 125.44, 70.28, 25.15, 21.16. GC conditions: Agilgent CP-Chirasil Dex CB (df = 0.25 μm, 0.32 mm i.d. × 25 m); carrier gas, N_2_ (flow 30 mL/min); injection temp, 280 °C; initial column temperature 100 °C, then progress rate, 5 °C/min; final column temperature, 200 °C for 5 min.

(*S*)-**5b**, 97% ee, t*_R_* = 7.183 min (minor) and t*_S_* = 7.589 min (major).

(*R*)-**5b**, 94% ee, t*_R_* = 7.139 min (major) and t*_S_* = 7.604 min (minor).

1H NMR (400 MHz, Chloroform-d) δ 7.28 (t, J = 7.2 Hz, 1H), 7.25 – 7.17 (m, 2H), 7.13 (d, J = 7.1 Hz, 1H), 4.89 (q, J = 5.7 Hz, 1H), 2.41 (s, 3H), 2.05 (s, 1H), 1.55 – 1.50 (m, 3H). 13C NMR (101 MHz, Chloroform-d) δ 138.21, 128.48, 128.27, 126.18, 122.51, 70.47, 25.17, 21.52. GC conditions: Agilgent CP-Chirasil Dex CB (df = 0.25 μm, 0.32 mm i.d. × 25 m); carrier gas, N_2_ (flow 30 mL/min); injection temp, 280 °C; initial column temperature 100 °C, then progress rate, 5 °C/min; final column temperature, 200 °C for 5 min.

(*S*)-**5c**, 99% ee, t*_R_* = 7.278 min (minor) and t*_S_* = 7.423 min (major).

(*R*)-**5c**, 98% ee, t*_R_* = 7.279 min (major) and t*_S_* = 7.545 min (minor).

1H NMR (400 MHz, Chloroform-d) δ 7.55 (d, J = 7.5 Hz, 1H), 7.23 (ddd, J = 27.3, 13.0, 6.6 Hz, 3H), 5.15 (q, J = 6.2 Hz, 1H), 2.39 (s, 3H), 2.03 (s, 1H), 1.50 (d, J = 6.3 Hz, 3H). 13C NMR (101 MHz, Chloroform-d) δ 143.94, 134.29, 130.43, 127.22, 126.44, 124.57, 66.85, 23.98, 18.97. GC conditions: Agilgent CP-Chirasil Dex CB (df = 0.25 μm, 0.32 mm i.d. × 25 m); carrier gas, N_2_ (flow 30 mL/min); injection temp, 280 °C; initial column temperature 100 °C, then progress rate, 5 °C/min; final column temperature.

(*S*)-**5d**, 99% ee, t*_R_* = 8.484 min (minor) and t*_S_* = 9.137 min (major).

(*R*)-**5d**, >99% ee, t*_R_* = 8.410 min (major) and t*_S_* not detected.

1H NMR (400 MHz, Chloroform-d) δ 7.36 – 7.30 (m, 2H), 6.91 (d, J = 8.5 Hz, 2H), 4.96 – 4.78 (m, 1H), 3.83 (s, 3H), 1.99 (d, J = 18.8 Hz, 1H), 1.50 (d, J = 6.4 Hz, 3H). 13C NMR (101 MHz, Chloroform-d) δ 159.03, 138.11, 126.73, 113.90, 70.00, 55.34, 25.07. GC conditions: Agilgent CP-Chirasil Dex CB (df = 0.25 μm, 0.32 mm i.d. × 25 m); carrier gas, N_2_ (flow 30 mL/min); injection temp, 280 °C; initial column temperature 100 °C, then progress rate, 5 °C/min; final column temperature, 200 °C for 5 min.

(*S*)-**5e**, 99% ee, t*_R_* = 26.444 min (minor) and t*_S_* = 26.603 min (major).

(*R*)-**5e**, 92% ee, t*_R_* = 26.305 min (major) and t*_S_* = 26.875 min (minor).

1H NMR (600 MHz, Chloroform-d) δ 7.36 – 7.30 (m, 2H), 7.02 (t, J = 8.7 Hz, 2H), 4.88 (s, 1H), 1.85 (d, J = 12.0 Hz, 1H), 1.47 (dd, J = 6.6, 1.9 Hz, 3H). 13C NMR (151 MHz, Chloroform-d) δ 162.94, 161.32, 141.55, 141.53, 127.06, 127.01, 115.31, 115.17, 69.76, 25.26. GC conditions: Agilgent CP-Chirasil Dex CB (df = 0.25 μm, 0.32 mm i.d. × 25 m); carrier gas, N_2_ (flow 30 mL/min); injection temp, 280 °C; initial column temperature 100 °C, then progress rate, 5 °C/min; final column temperature, 200 °C for 5 min.

(*S*)-**5f**, 96% ee, t*_R_* =6.670 min (minor) and t*_S_* = 7.075 min (major).

(*R*)-**5f**, 98% ee, t*_R_* = 6.648 min (major) and t*_S_* = 7.097 min (minor).

1H NMR (400 MHz, Chloroform-d) δ 7.34 (q, J = 7.4 Hz, 1H), 7.20 – 7.10 (m, 2H), 6.99 (t, J = 8.0 Hz, 1H), 4.93 (q, J = 6.4 Hz, 1H), 1.90 (s, 1H), 1.53 (d, J = 6.2 Hz, 3H). 13C NMR (101 MHz, Chloroform-d) δ 164.29, 161.84, 148.62, 148.55, 130.08, 130.00, 121.00, 120.98, 114.36, 114.15, 112.47, 112.25, 25.25. GC conditions: Agilgent CP-Chirasil Dex CB (df = 0.25 μm, 0.32 mm i.d. × 25 m); carrier gas, N_2_ (flow 30 mL/min); injection temp, 280 °C; initial column temperature 100 °C, then progress rate, 5 °C/min; final column temperature, 200 °C for 5 min.

(*S*)-**5g**, 98% ee, t*_R_* =11.038 min (minor) and t*_S_* = 11.195 min (major).

(*R*)-**5g**, 99% ee, t*_R_* = 11.013 min (major) and t*_S_* = 11.415 min (minor).

1H NMR (500 MHz, Chloroform-d) δ 7.40 – 7.26 (m, 2H), 7.18 (d, J = 9.5 Hz, 1H), 7.15 – 7.08 (m, 1H), 5.10 – 5.03 (m, 1H), 3.59 (d, J = 4.9 Hz, 1H). 13C NMR (125 MHz, Common NMR Solvents) δ 161.88, 133.47, 129.37, 127.88, 125.98, 115.19, 65.75, 23.95. GC conditions: Agilgent CP-Chirasil Dex CB (df = 0.25 μm, 0.32 mm i.d. × 25 m); carrier gas, N_2_ (flow 30 mL/min); injection temp, 280 °C; initial column temperature 100 °C, then progress rate, 5 °C/min; final column temperature, 200 °C for 5 min.

(*S*)-**5h**, 98% ee, t*_R_* = 6.84in (minor) and t*_S_* = 6.661in (major).

(*R*)-**5h**, 97% ee, t*_R_* = 6.294 min (major) and t*_S_* = 6.671 min (minor).

1H NMR (600 MHz, Chloroform-d) δ 7.46 (d, J = 8.3 Hz, 2H), 7.24 (d, J = 8.3 Hz, 2H), 4.86 (q, J = 6.5 Hz, 1H), 1.86 (s, 1H), 1.47 (d, J = 5.5 Hz, 3H). 13C NMR (151 MHz, Chloroform-d) δ 144.79, 131.56, 127.15, 121.16, 69.77, 25.23. GC conditions: Agilgent CP-Chirasil Dex CB (df = 0.25 μm, 0.32 mm i.d. × 25 m); carrier gas, N_2_ (flow 30 mL/min); injection temp, 280 °C; initial column temperature 100 °C, then progress rate, 5 °C/min; final column temperature, 200 °C for 5 min.

(*S*)-**5i**, 96% ee, t*_R_* =10.444 min (minor) and t*_S_* = 10.794 min (major).

(*R*)-**5i**, 94% ee, t*_R_* = 10.360 min (major) and t*_S_* = 10.931 min (minor).

1H NMR (400 MHz, Chloroform-d) δ 7.40 (s, 1H), 7.34 – 7.22 (m, 3H), 4.89 (q, J = 6.1 Hz, 1H), 2.09 (d, J = 12.9 Hz, 1H), 1.51 (dd, J = 6.4, 1.5 Hz, 3H). 13C NMR (101 MHz, Chloroform-d) δ 147.93, 134.41, 129.84, 127.57, 125.69, 123.60, 69.82, 25.25.GC conditions: Agilgent CP-Chirasil Dex CB (df = 0.25 μm, 0.32 mm i.d. × 25 m); carrier gas, N_2_ (flow 30 mL/min); injection temp, 280 °C; initial column temperature 100 °C, then progress rate, 5 °C/min; final column temperature, 200 °C for 5 min.

(*S*)-**5j**, 98% ee, t*_R_* =15.863 min (minor) and t*_S_* = 16.019 min (major).

(*R*)-**5j**, 98% ee, t*_R_* = 15.848 min (major) and t*_S_* = 16.054 min (minor).

1H NMR (400 MHz, Chloroform-d) δ 7.63 (d, J = 7.4 Hz, 1H), 7.39 – 7.20 (m, 3H), 5.32 (td, J = 6.2, 2.5 Hz, 1H), 2.03 (d, J = 19.9 Hz, 1H), 1.53 (dd, J = 6.3, 2.7 Hz, 3H). 13C NMR (101 MHz, Chloroform-d) δ 143.14, 131.70, 129.46, 128.45, 127.27, 126.48, 67.00, 23.56. GC conditions: Agilgent CP-Chirasil Dex CB (df = 0.25 μm, 0.32 mm i.d. × 25 m); carrier gas, N_2_ (flow 30 mL/min); injection temp, 280 °C; initial column temperature 100 °C, then progress rate, 5 °C/min; final column temperature, 200 °C for 5 min.

(*S*)-**5k**, 99% ee, t*_R_* =10.175 min (minor) and t*_S_* = 11.002 min (major).

(*R*)-**5k**, 97% ee, t*_R_* = 9.934 min (major) and t*_S_* = 10.992 min (minor).

1H NMR (600 MHz, Chloroform-d) δ 7.46 (d, J = 8.3 Hz, 2H), 7.24 (d, J = 8.3 Hz, 2H), 4.86 (q, J = 6.5 Hz, 1H), 1.86 (s, 1H), 1.47 (d, J = 5.5 Hz, 3H). 13C NMR (151 MHz, Chloroform-d) δ 144.79, 131.56, 127.15, 121.16, 69.77, 25.23. GC conditions: Agilgent CP-Chirasil Dex CB (df = 0.25 μm, 0.32 mm i.d. × 25 m); carrier gas, N_2_ (flow 30 mL/min); injection temp, 280 °C; initial column temperature 100 °C, then progress rate, 5 °C/min; final column temperature, 200 °C for 5 min.

(*S*)-**5l**, >99% ee, t*_R_* not detected and t*_S_* = 12.669 min (major).

(*R*)-**5l**, 98% ee, t*_R_* = 12.088 min (major) and t*_S_* = 12.780 min (minor).

1H NMR (400 MHz, Chloroform-d) δ 7.57 (s, 1H), 7.43 (d, J = 7.7 Hz, 1H), 7.32 (d, J = 7.6 Hz, 1H), 7.24 (t, J = 7.7 Hz, 1H), 4.90 (q, J = 6.4 Hz, 1H), 1.87 (s, 1H), 1.51 (dd, J = 6.4, 1.5 Hz, 3H). 13C NMR (101 MHz, Chloroform-d) δ 148.18, 130.53, 130.15, 128.63, 124.07, 122.68, 69.81, 25.31. GC conditions: Agilgent CP-Chirasil Dex CB (df = 0.25 μm, 0.32 mm i.d. × 25 m); carrier gas, N_2_ (flow 30 mL/min); injection temp, 280 °C; initial column temperature 100 °C, then progress rate, 5 °C/min; final column temperature, 200 °C for 5 min.

(*S*)-**5m**, 98% ee, t*_R_* = 11.747 min (minor) and t*_S_* = 11.958 min (major).

(*R*)-**5m**, 99% ee, t*_R_* = 11.693 min (major) and t*_S_* = 12.058 min (minor).

1H NMR (400 MHz, Chloroform-d) δ 7.62 (d, J = 7.6 Hz, 1H), 7.54 (d, J = 7.3 Hz, 1H), 7.37 (t, J = 6.6 Hz, 1H), 7.21 – 7.08 (m, 1H), 5.41 – 5.12 (m, 1H), 1.97 (s, 1H), 1.52 (dd, J = 6.3, 2.4 Hz, 3H). 13C NMR (101 MHz, Chloroform-d) δ 144.70, 132.72, 128.82, 127.91, 126.74, 121.77, 69.24, 23.63. GC conditions: Agilgent CP-Chirasil Dex CB (df = 0.25 μm, 0.32 mm i.d. × 25 m); carrier gas, N_2_ (flow 30 mL/min); injection temp, 280 °C; initial column temperature 100 °C, then progress rate, 5 °C/min; final column temperature, 200 °C for 5 min.

(*S*)-**5n**, 99% ee, t*_R_* = 11.924 min (minor) and t*_S_* = 13.035 min (major).

(*R*)-**5n**, 98% ee, t*_R_* = 12.048 min (major) and t*_S_* = 13.058 min (minor).

1H NMR (600 MHz, Chloroform-d) δ 7.26 (d, J = 1.6 Hz, 2H), 7.24 (d, J = 8.1 Hz, 2H), 7.15 (t, J = 6.6 Hz, 1H), 4.04 (q, J = 6.6 Hz, 1H), 1.54 (s, 2H), 1.31 (d, J = 6.7 Hz, 3H). 13C NMR (151 MHz, Chloroform-d) δ 147.79, 128.48, 126.80, 125.68, 51.34, 25.65. GC conditions: Agilgent CP-Chirasil Dex CB (df = 0.25 μm, 0.32 mm i.d. × 25 m); carrier gas, N_2_ (flow 30 mL/min); injection temp, 280 °C; initial column temperature 100 °C, then progress rate, 5 °C/min; final column temperature, 200 °C for 5 min; t_S_ = 10.976 min (minor) and t*_R_* = 11.229 min (major).

1H NMR (600 MHz, Chloroform-d) δ 7.24 (d, J = 8.0 Hz, 2H), 7.14 (d, J = 7.8 Hz, 2H), 4.09 (q, J = 6.6 Hz, 1H), 2.34 (s, 3H), 1.38 (d, J = 6.6 Hz, 3H). 13C NMR (151 MHz, Chloroform-d) δ 144.88, 136.34, 129.14, 125.58, 51.03, 25.67, 21.00. GC conditions: Agilgent CP-Chirasil Dex CB (df = 0.25 μm, 0.32 mm i.d. × 25 m); carrier gas, N_2_ (flow 30 mL/min); injection temp, 280 °C; initial column temperature 100 °C, then progress rate, 5 °C/min; final column temperature, 200 °C for 5 min; t*_S_* = 12.186 min (minor) and t*_R_* =12.525 min (major).

1H NMR (600 MHz, Chloroform-d) δ 7.40 – 7.27 (m, 2H), 7.19 (dd, J = 46.5, 7.6 Hz, 2H), 4.18 (q, J = 6.6 Hz, 1H), 2.45 (s, 3H), 1.97 (s, 2H), 1.49 (d, J = 6.6 Hz, 3H).13C NMR (151 MHz, Chloroform-d) δ 147.59, 138.10, 128.41, 127.58, 126.43, 122.71, 51.29, 25.50, 21.44. GC conditions: Agilgent CP-Chirasil Dex CB (df = 0.25 μm, 0.32 mm i.d. × 25 m); carrier gas, N_2_ (flow 30 mL/min); injection temp, 280 °C; initial column temperature 100 °C, then progress rate, 5 °C/min; final column temperature, 200 °C for 5 min; tR = 10.769 min (minor) and 11.409 min (major).

1H NMR (600 MHz, Chloroform-d) δ 7.60 (d, J = 7.7 Hz, 1H), 7.36 (dt, J = 8.2, 4.2 Hz, 1H), 7.27 (t, J = 1.3 Hz, 1H), 7.26 (d, J = 1.0 Hz, 1H), 4.50 (q, J = 6.6 Hz, 1H), 2.49 (s, 3H), 1.49 (d, J = 6.6 Hz, 3H).13C NMR (151 MHz, Chloroform-d) δ 145.67, 134.43, 130.38, 126.46, 126.40, 124.17, 46.85, 24.53, 19.02. GC conditions: Agilgent CP-Chirasil Dex CB (df = 0.25 μm, 0.32 mm i.d. × 25 m); carrier gas, N_2_ (flow 30 mL/min); injection temp, 280 °C; initial column temperature 100 °C, then progress rate, 5 °C/min; final column temperature, 200 °C for 5 min; t_S_ = 9.661 min (minor) and t*_R_* = 9.984 min (major).

1H NMR (600 MHz, Chloroform-d) δ 7.22 (dd, J = 8.2, 0.7 Hz, 2H), 6.85 (d, J = 8.3 Hz, 2H), 4.09 (q, J = 5.5 Hz, 1H), 3.78 (s, 3H), 2.69 (d, J = 5.7 Hz, 2H), 1.39 (d, J = 5.3 Hz, 3H). 13C NMR (125 MHz, Common NMR Solvents) δ 158.90, 139.69, 127.08, 113.71, 55.32, 50.62, 24.69. GC conditions: Agilgent CP-Chirasil Dex CB (df = 0.25 μm, 0.32 mm i.d. × 25 m); carrier gas, N_2_ (flow 30 mL/min); injection temp, 280 °C; initial column temperature 100 °C, then progress rate, 5 °C/min; final column temperature, 200 °C for 5 min; t_S_ = 14.554 min (minor) and t*_R_* = 14.972 min (major).

1H NMR (400 MHz, Chloroform-d) δ 7.39 – 7.31 (m, 2H), 7.05 (dt, J = 8.5, 4.3 Hz, 2H), 4.26 – 4.05 (m, 1H), 1.78 (d, J = 7.9 Hz, 2H), 1.41 (dd, J = 6.4, 3.0 Hz, 3H). 13C NMR (101 MHz, Chloroform-d) δ 162.99, 160.56, 143.37, 143.34, 127.33, 127.25, 115.31, 115.10, 50.71, 25.84. GC conditions: Agilgent CP-Chirasil Dex CB (df = 0.25 μm, 0.32 mm i.d. × 25 m); carrier gas, N_2_ (flow 30 mL/min); injection temp, 280 °C; initial column temperature 100 °C, then progress rate, 5 °C/min; final column temperature, 200 °C for 5 min; t_S_ = 10.013 min (minor) and t*_R_* = 10.469 min (major).

1H NMR (400 MHz, Chloroform-d) δ 7.31 (d, J = 8.8 Hz, 1H), 7.12 (dd, J = 15.9, 8.9 Hz, 2H), 7.02 – 6.87 (m, 1H), 4.15 (q, J = 6.5 Hz, 1H), 1.70 (d, J = 4.0 Hz, 2H), 1.41 (d, J = 6.6 Hz, 3H). 13C NMR (101 MHz, Chloroform-d) δ 164.34, 161.89, 150.56, 150.50, 130.01, 129.93, 121.43, 121.41, 113.75, 113.54, 112.77, 112.55, 51.01, 25.65. GC conditions: Agilgent CP-Chirasil Dex CB (df = 0.25 μm, 0.32 mm i.d. × 25 m); carrier gas, N_2_ (flow 30 mL/min); injection temp, 280 °C; initial column temperature 100 °C, then progress rate, 5 °C/min; final column temperature, 200 °C for 5 min; t_S_ = 9.181 min (minor) and t*_R_* = 9.993 min (major).

1H NMR (400 MHz, Chloroform-d) δ 7.44 (t, J = 7.5 Hz, 1H), 7.24 (q, J = 6.6, 6.0 Hz, 1H), 7.15 (t, J = 7.4 Hz, 1H), 7.09 – 7.00 (m, 1H), 4.42 (q, J = 6.7 Hz, 1H), 1.75 – 1.72 (m, 2H), 1.46 (d, J = 6.7 Hz, 3H). 13C NMR (101 MHz, Chloroform-d) δ 161.66, 159.23, 134.52, 134.39, 128.20, 128.12, 126.78, 126.73, 124.29, 124.26, 115.58, 115.36, 45.44, 24.07. GC conditions: Agilgent CP-Chirasil Dex CB (df = 0.25 μm, 0.32 mm i.d. × 25 m); carrier gas, N_2_ (flow 30 mL/min); injection temp, 280 °C; initial column temperature 100 °C, then progress rate, 5 °C/min; final column temperature, 200 °C for 5 min; t_S_ = 9.444 min (minor) and t*_R_* = 9.914 min (major).

1H NMR (400 MHz, Chloroform-d) δ 7.32 (s, 4H), 4.18 – 4.11 (m, 1H), 1.65 (s, 2H), 1.40 (d, J = 6.6 Hz, 3H). 13C NMR (101 MHz, Chloroform-d) δ 146.14, 132.42, 128.61, 127.22, 50.78. GC conditions: Agilgent CP-Chirasil Dex CB (df = 0.25 μm, 0.32 mm i.d. × 25 m); carrier gas, N_2_ (flow 30 mL/min); injection temp, 280 °C; initial column temperature 100 °C, then progress rate, 5 °C/min; final column temperature, 200 °C for 5 min; t_S_ = 14.075min (minor) and t*_R_* = 14.524min (major).

1H NMR (400 MHz, Chloroform-d) δ 7.39 (s, 1H), 7.27 (td, J = 13.3, 12.2, 5.5 Hz, 3H), 4.14 (q, J = 6.6 Hz, 1H), 1.67 (d, J = 4.9 Hz, 2H), 1.41 (d, J = 6.6 Hz, 3H). 13C NMR (101 MHz, Chloroform-d) δ 149.85, 134.36, 129.81, 126.99, 126.06, 124.03, 51.02, 25.66. GC conditions: Agilgent CP-Chirasil Dex CB (df = 0.25 μm, 0.32 mm i.d. × 25 m); carrier gas, N_2_ (flow 30 mL/min); injection temp, 280 °C; initial column temperature 100 °C, then progress rate, 5 °C/min; final column temperature, 200 °C for 5 min; t_S_ = 13.011 min (minor) and t*_R_* = 13.493 min (major).

1H NMR (400 MHz, Chloroform-d) δ 7.55 (d, J = 7.6 Hz, 1H), 7.36 (d, J = 7.9 Hz, 1H), 7.30 (t, J = 7.4 Hz, 1H), 7.19 (t, J = 7.6 Hz, 1H), 4.63 – 4.49 (m, 1H), 1.66 (s, 2H), 1.42 (d, J = 6.6 Hz, 3H). 13C NMR (101 MHz, Chloroform-d) δ 144.65, 132.71, 129.65, 127.89, 127.24, 126.37, 47.66, 23.70. GC conditions: Agilgent CP-Chirasil Dex CB (df = 0.25 μm, 0.32 mm i.d. × 25 m); carrier gas, N_2_ (flow 30 mL/min); injection temp, 280 °C; initial column temperature 100 °C, then progress rate, 5 °C/min; final column temperature, 200 °C for 5 min; t_S_ = 12.976 min (minor) and t*_R_* = 13.497 min (major).

1H NMR (400 MHz, Chloroform-d) δ 7.49 (d, J = 8.3 Hz, 2H), 7.28 (d, J = 8.2 Hz, 2H), 4.14 (q, J = 6.5 Hz, 1H), 1.68 (s, 2H), 1.41 (d, J = 6.6 Hz, 3H). 13C NMR (101 MHz, Chloroform-d) δ 146.67, 131.56, 127.62, 120.48, 50.84, 25.72. GC conditions: Agilgent CP-Chirasil Dex CB (df = 0.25 μm, 0.32 mm i.d. × 25 m); carrier gas, N_2_ (flow 30 mL/min); injection temp, 280 °C; initial column temperature 100 °C, then progress rate, 5 °C/min; final column temperature, 200 °C for 5 min; t_S_ = 15.788 min (minor) and t*_R_* = 16.270 min (major).

1H NMR (400 MHz, Chloroform-d) δ 7.54 (s, 1H), 7.39 (d, J = 7.7 Hz, 1H), 7.29 (s, 1H), 7.22 (t, J = 7.7 Hz, 1H), 4.12 (q, J = 6.4 Hz, 1H), 1.69 (s, 2H), 1.40 (d, J = 6.6 Hz, 3H). GC conditions: Agilgent CP-Chirasil Dex CB (df = 0.25 μm, 0.32 mm i.d. × 25 m); carrier gas, N_2_ (flow 30 mL/min); injection temp, 280 °C; initial column temperature 100 °C, then progress rate, 5 °C/min; final column temperature, 200 °C for 5 min; t_S_ = 14.839 min (minor) and t*_R_* = 15.208 min (major).

1H NMR (400 MHz, Chloroform-d) δ 7.56 (d, J = 7.1 Hz, 2H), 7.35 (t, J = 7.4 Hz, 1H), 7.13 (t, J = 7.6 Hz, 1H), 4.56 (s, 1H), 1.77 (s, 2H), 1.43 (d, J = 6.5 Hz, 3H). GC conditions: Agilgent CP-Chirasil Dex CB (df = 0.25 μm, 0.32 mm i.d. × 25 m); carrier gas, N_2_ (flow 30 mL/min); injection temp, 280 °C; initial column temperature 100 °C, then progress rate, 5 °C/min; final column temperature, 200 °C for 5 min; t_S_ = 13.046 min (minor) and t*_R_* = 13.244 min (major)

1H NMR (600 MHz, Chloroform-d) δ 7.17 (dd, J = 5.0, 1.3 Hz, 1H), 6.94 (dd, J = 5.0, 3.5 Hz, 1H), 6.92 – 6.90 (m, 1H), 4.37 (q, J = 6.5 Hz, 1H), 1.72 (s, 2H), 1.49 (d, J = 6.6 Hz, 3H). 13C NMR (151 MHz, Chloroform-d) δ 152.86, 126.59, 123.33, 122.12, 47.35, 26.34. GC conditions: Agilgent CP-Chirasil Dex CB (df = 0.25 μm, 0.32 mm i.d. × 25 m); carrier gas, N_2_ (flow 30 mL/min); injection temp, 280 °C; initial column temperature 100 °C, then progress rate, 5 °C/min; final column temperature, 200 °C for 5 min; t_S_ = 10.547 min (minor) and t*_R_* = 10.892 min (major).

1H NMR (600 MHz, Chloroform-d) δ 7.69 (d, J = 2.8 Hz, 1H), 7.22 (d, J = 2.8 Hz, 1H), 4.41 (qd, J = 6.6, 2.4 Hz, 1H), 1.90 (s, 2H), 1.53 (d, J = 6.7 Hz, 3H). 13C NMR (151 MHz, Chloroform-d) δ 178.41, 142.46, 118.31, 49.75, 24.72. GC conditions: Agilgent CP-Chirasil Dex CB (df = 0.25 μm, 0.32 mm i.d. × 25 m); carrier gas, N_2_ (flow 30 mL/min); injection temp, 280 °C; initial column temperature 100 °C, then progress rate, 5 °C/min; final column temperature, 200 °C for 5 min; t_S_ = 10.228 min (minor) and t*_R_* = 10.419 min (major).

# Characterization

Scanning electron microscopy (SEM) images were recorded on Nova Nano SEM450 field-emission microscope at an accelerating voltage of 200 kV. All samples were dispersed in absolute ethyl alcohol ultrasonically and were dropped on a piece of monocrystalline silicon. Transmission electron microscopy (TEM) micrographs of samples were obtained with a JEM-2100 microscope operated at an acceleration voltage of 1.0 kV. Samples powders were dispersed in ethanol for 30 min with sonication. Then the mixture was dropped onto copper grid using a micropipette. Nitrogen adsorption-desorption experiments were carried out on a micromeritics ASAP 2020 gas sorptometer at 77 K. The samples were outgassed at 120 °C for 12 h. The specific surface area was calculated by BrunauerEmmett-Teller (BET) method. The total pore volume of the samples was estimated from the amount adsorbed at the highest P/P_0_ (ca. 0.99) by Barrett–Joyner–Halenda (BJH) model. Water contact angles were measured by KRUSS DAS30 optical contact angle measuring instrument. X-ray photoelectron spectrometry (XPS) was performed on a PHI-Vesoprobe 5000 Ш X-ray photoelectron spectrometer. The content of Rh was quantified by an Optima 7300 DV inductively coupled plasma atomic emission spectrometer (ICP-AES). X-ray diffraction (XRD) studies were carried out on a Japan Rigaku SmartLabTM rotation anode X-ray diffractometer equipped or Holland X’Pert PRO fixed anode X-ray diffractometer equipped with graphite monochromatized Cu Kα radiation (λ = 1.54 Å).

# XAFS measurements and analysis details

Data reduction, data analysis, and EXAFS fitting were performed and analyzed with the Athena and Artemis programs of the Demeter data analysis packages^[6]^ that utilizes the FEFF6 program^[7]^ to fit the EXAFS data. The energy calibration of the sample was conducted through standard and Co foil, which as a reference was simultaneously measured. A linear function was subtracted from the pre-edge region, then the edge jump was normalized using Athena software. The *χ(k)* data were isolated by subtracting a smooth, third-order polynomial approximating the absorption background of an isolated atom. The *k^3^*-weighted *χ(k)* data were Fourier transformed after applying a HanFeng window function (*Δk* = 1.0). For EXAFS modeling. The global amplitude EXAFS (*CN*, *R*, *σ*^2^ and Δ*E*_0_) were obtained by nonlinear fitting, with least-squares refinement, of the EXAFS equation to the Fourier-transformed data in *R*-space, using Artemis software, EXAFS of the Co foil are fitted and the obtained amplitude reduction factor *S_0_^2^* value (0.721) was set in the EXAFS analysis to determine the coordination numbers (*CNs*) in sample.

# Computational details

All first-principles calculations are performed using the Vienna Ab initio Simulation Package (VASP)^[8]^. The generalized gradient approximation (GGA) with the functional described by Perdew–Burke–Ernzerhof (PBE) functional was used for structure optimization^[9]^. The projector augmented wave (PAW)^[10]^ method was used to describe the wave function of the core region, while the valence wave function were extended to a linear combination of plane waves with 450 eV cut-off energy. The weak Van der Waals interaction is described by dispersion correction PBE + D3^[11]^. The spin-polarized method was applied to all calculations. In geometric optimization, the total energy was converged to 10^-5^eV, and the Hellmann-Feynman force on each relaxed atom was less than 0.02 eV/Å. The Brillouin zone integration is performed using a gamma k-mesh.

# Calculation formulas

Residence time (RT) was calculated as follows:

(1)


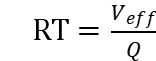


(2)


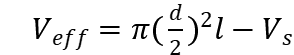


Where *V_eff_* is the effective bioreactor volume (mL); *Q* is the flow rate (mL/min); *d* is the packed column inner diameter (cm); *l* is the packed column length (cm); and *Vs* is the biocatalyst volume (mL).

The space-time yield (STY) in continuous flow reactions was calculated as follows:

(3)


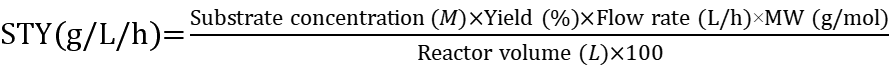


Catalyst productivity is defined as the amount of product per unit of catalyst (e.g. g_AcPO_ g_Co_^-1^). Co catalyst productivity was calculated by summing the amounts of AcPO obtained in each of the time points after steady stage and dividing them by the amount of Co used.

The half-life of bioreactors was calculated as follows:

(4)


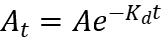


(5)


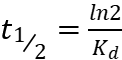


# Figure S1. FTIR spectra of CoPc and CoN_4_SA-POase.

#

# Figures

# Figure S2. SEM images of (a) PCN and (b) CoN_4_SA-POase. (c) TEM image of CoNP@PCN

# Figure S3. N_2_ absorption and desorption curves of PCN, CoNP@PCN and CoN_4_SA-POase.

# Figure S4. The structure model of CoN_4_SA-POase.


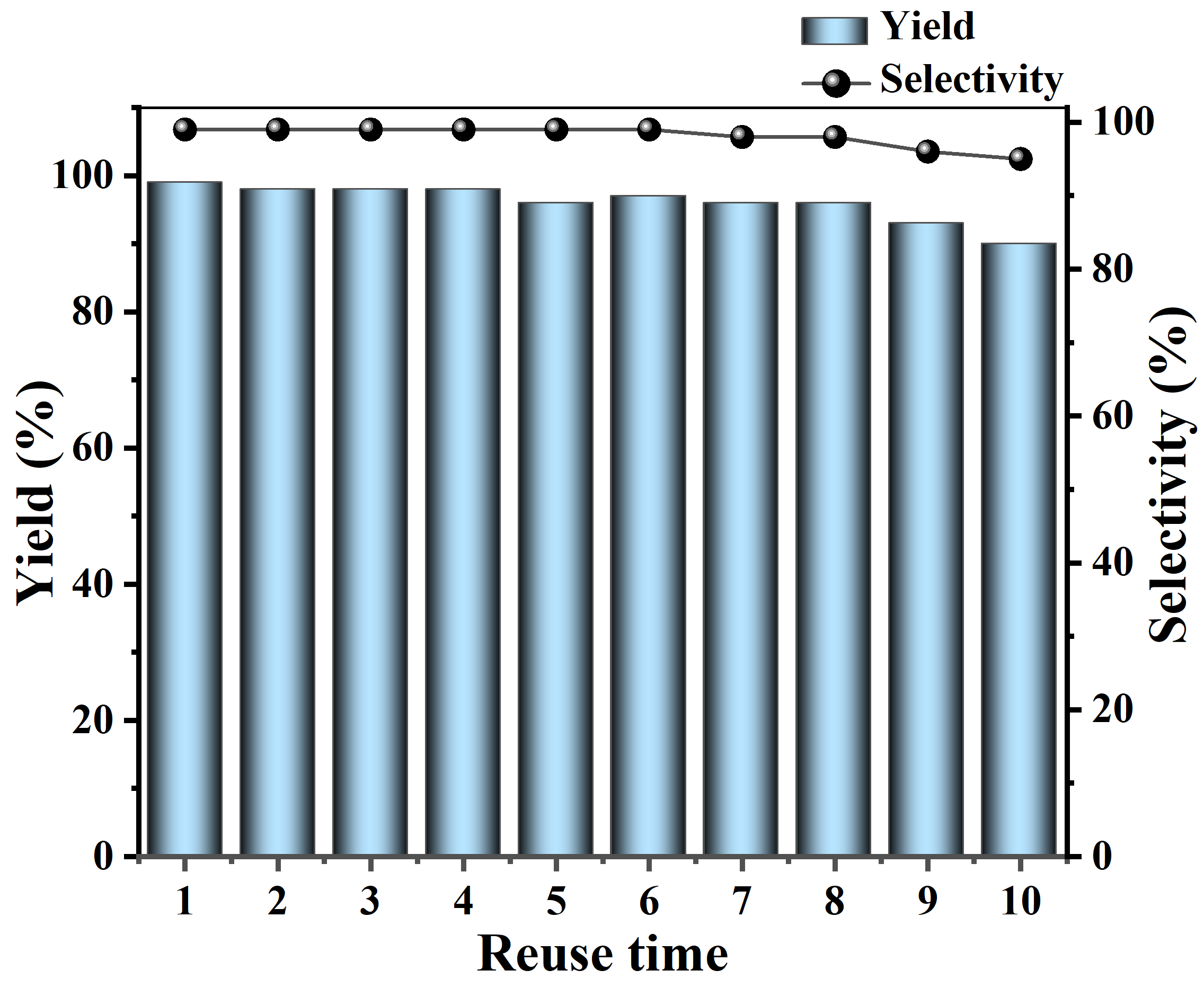


# Figure S5. Reusability of the CoN_4_SA-POase catalyst for catalyzing 1a oxidation.

# Figure S6. TEM of CoN_4_SA-POase after 10 reuse times.

# Figure S6. XRD patterns of CoN_4_SA-POase before use and after 10th reuse.

# Figure S8. Reaction time curves of CoN_4_SA-POase-catalyzed oxidation of 1a.

# Figure S9. DMPO (5,5-dimethyl-1-pyrroline N-oxide) spin-trapping EPR experiment. The spectrum was recorded after 1 min upon introducing DMPO to the reaction system. Ethylbenzene was used as the substrate.

# Figure S10. Operational stability of the flow system in the continuous synthesis of 2a.

# Tables

# Table S1. EXAFS fitting parameters at the Co K–edge for various samples.

| Sample | Shell | *CN^a^* | *R* (Å)*^b^* | *σ*^2^ (Å^2^)*^c^* | Δ*E*_0_ (eV)*^d^* | *R* factor |
| --- | --- | --- | --- | --- | --- | --- |
| Co foil | Co-Co | 12 | 2.49±0.01 | 0.0063 | 6.5 | 0.0011 |
| CoPc | Co-N | 4.1±0.3 | 1.90±0.01 | 0.0017 | 12.3 | 0.0166 |
| **CoN_4_SA-POase** | **Co-C/N** | **3.9±0.2** | **1.90±0.01** | **0.0089** | **5.6** | **0.0067** |

*^a^ CN*, coordination number; *^b^ R*, distance between absorber and backscatter atoms; *^c^ σ*^2^, Debye-Waller factor to account for both thermal and structural disorders; *^d^ ΔE*_0_, inner potential correction; *R* factor indicates the goodness of the fit. S_0_^2^ was fixed to 0.721, according to the experimental EXAFS fit of Co foil by fixing CN as the known crystallographic value. A reasonable range of EXAFS fitting parameters: 0.600 < *Ѕ*_0_^2^ < 1.000; *CN >* 0; *σ*^2^ > 0 Å^2^; |Δ*E*_0_| < 15 eV; *R* factor < 0.02.

# Table S2. Comparison of Co-N coordination modes.

| Catalyst | *CN^a^* | *R* (Å)*^b^* | *CB^c^* | Reference |
| --- | --- | --- | --- | --- |
| Co/ZIF-800 | 4 | 1.89 | Co-N2 | [12] |
| Co/ZIF-900 | 3.5 | 1.88 | Co-N2 |  |
| Co/ZIF-1000 | 3.1 | 1.88 | Co-N2 |  |
| Co/g-C_3_N_4_-0.9 | 3 | 1.85/1.86 | Co-N2 | [13] |
| Co SACs | 2.3 | 1.95 | Co-N2 | [14] |
| Co-CCN/PTI-4 | 4 | 2.31 | Co-N2 | [15] |
| Co-CCN/PTI-40 | 4 | 2.10/2.52 |  |  |
| CoSA-N_3_-C | 3 | 1.94 | Co-N2 | [16] |
| Co-C_3_N_4_ | 4 | 2.00 | Co-N2 | [17] |
| CoNxOy-900 | 3 | 1.94 | Co-N2 | [18] |
| SA-Co-CN/g-C_3_N_4_ | 4.2 | 2.00 | Co-N2 | [19] |
| BM-PCN/Co-c | 5 | 2.04 | Co-N2/O | [20] |
| **CoN_4_SA-POase** | **4** | **1.86/1.90** | Co-N1/N2 | **This work** |

*^a^ CN*, coordination number. *^b^ R*, distance between Co and N atom. *^c^* *CB*, coordination bond.

Table S3. Comparison of ethylbenzene oxidation by different catalytic systems**.**

| Catalyst | Oxidant | T*^c^* (^o^C) | Time (h) | **1a**  Conv. (%) | Sel. (%) | TOF (h^-1^) | Ref |
| --- | --- | --- | --- | --- | --- | --- | --- |
| SACo@PCN | PMS*^a^* | 60 | 15 | 97.5 | 95.6 | 2.3 | [2] |
| Pd@N-doped carbon | O_2_/1 atm | 120 | 20 | 14.2 | 94 | 245 | [21] |
| Co SACs | O_2_ | 120 | 24 | 46 | 97 | 19.6 | [14] |
| Co@NCNT | O_2_/0.8 MPa | 120 | 5 | 68.1 | 93.2 | 93.4 | [22] |
| Co/oxide | TBHP*^b^* | 120 | 12 | 69.5 | 80.4 | 5.1 | [23] |
| Cu-BTC-SiO_2_ | TBHP | 60 | 6 | 99 | 99 | 3.3 | [24] |
| Co/AC | TBHP | 80 | 4 | 47.9 | 83.5 | 15.7 | [25] |
| Ag/SBA-15 | TBHP | 90 | 5 | 92 | 99 | 9.9 | [26] |
| CoCuAlLDH/graphene | TBHP | 120 | 12 | 96.8 | 95.4 | 6.1 | [27] |
| Co-N-C | TBHP | 80 | 12 | 99 | 96 | 27.9 | [28] |
| Au/LDH | TBHP | 140 | 24 | 39 | 91 | 5240 | [29] |
| Fe-N-C-700 | TBHP | RT*^d^* | 7 | 99 | 99 | 24.7 | [30] |
| Co-ISA/CNB | TBHP | RT | 48 | 98 | 99 | 58 | [31] |
| **CoN_4_SA-POase** | TBHP | 40 | 15 | 96 | 99 | 56 | Herein |

*^a^* Peroxymonosulfate. *^b^* Tert-butyl hydroperoxide. *^c^* Reaction temperature. *^d^* Room temperature.

# NMR spectra


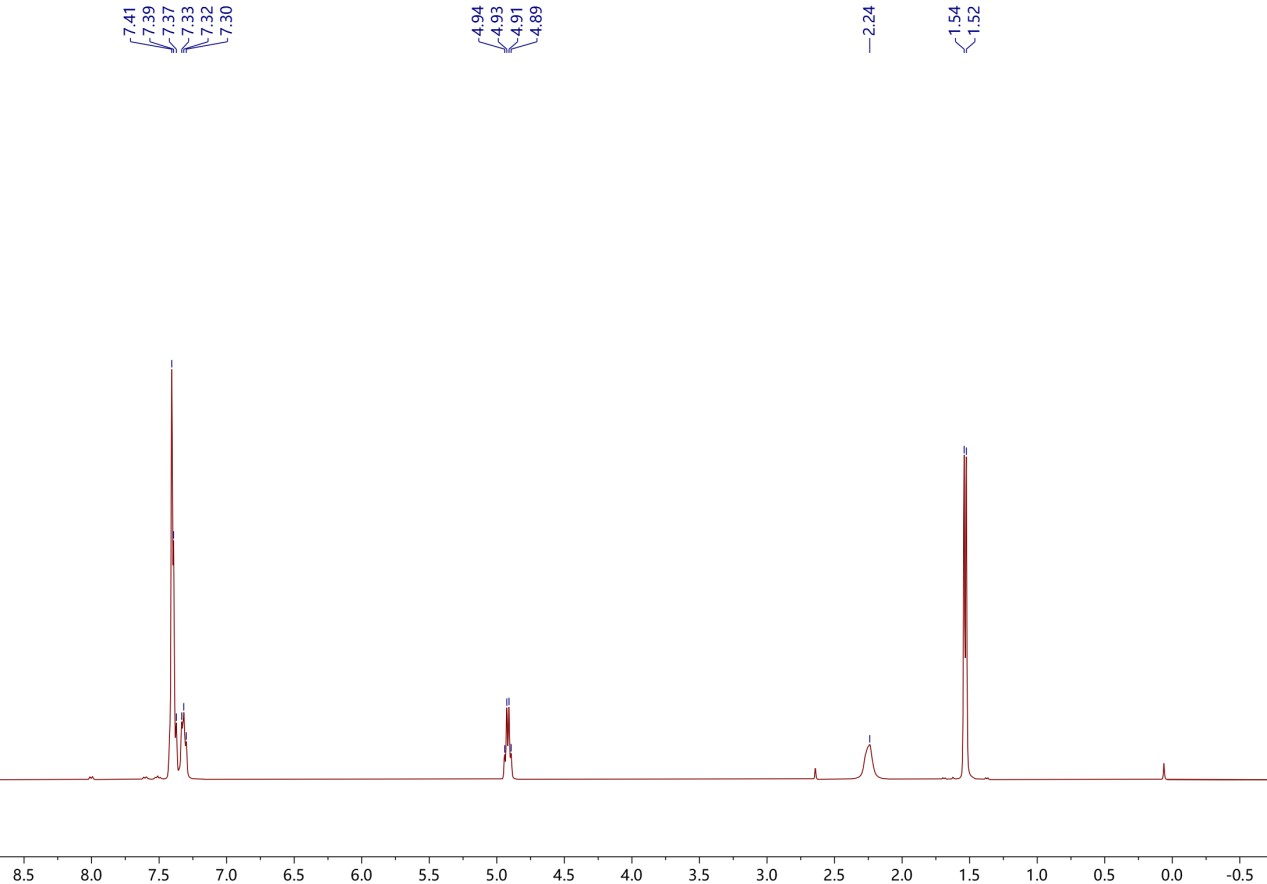

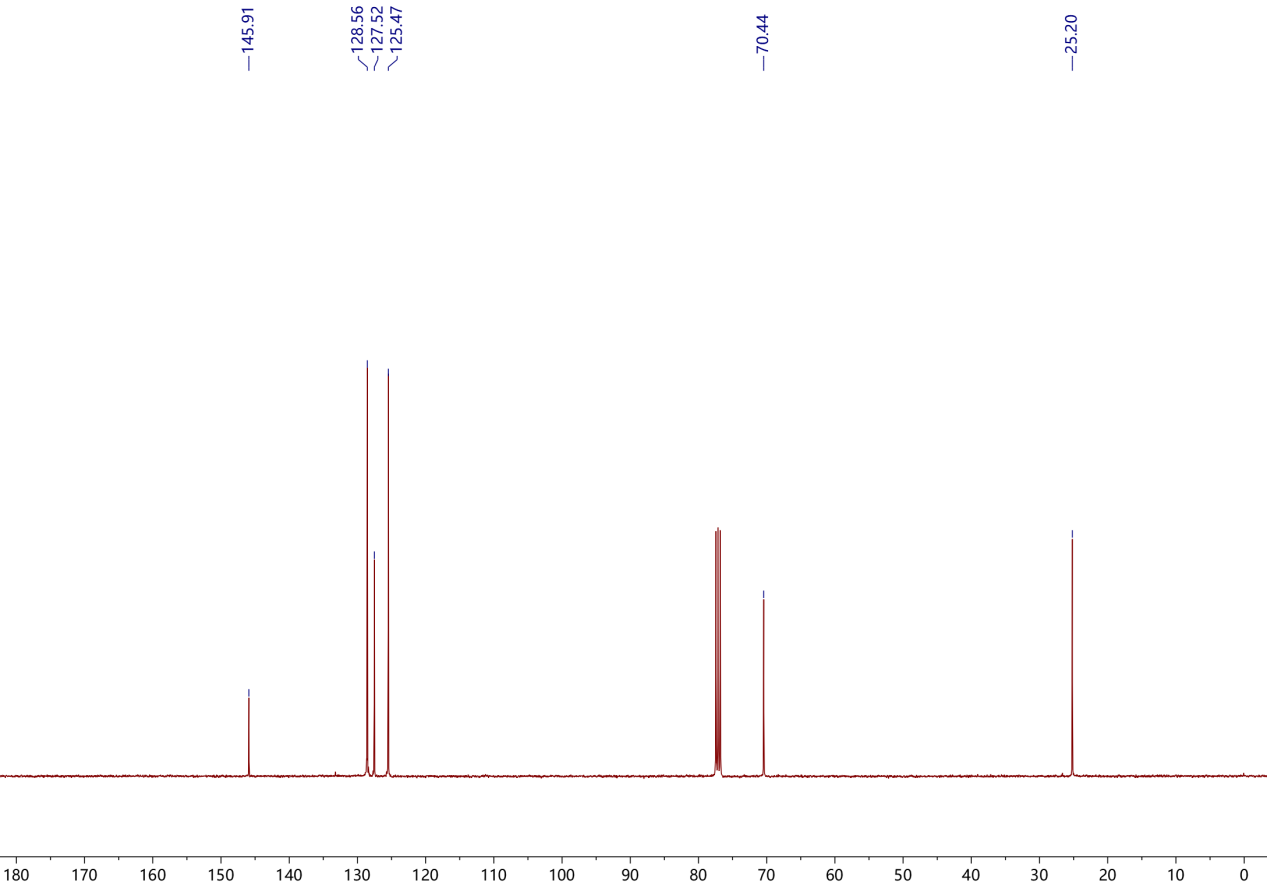

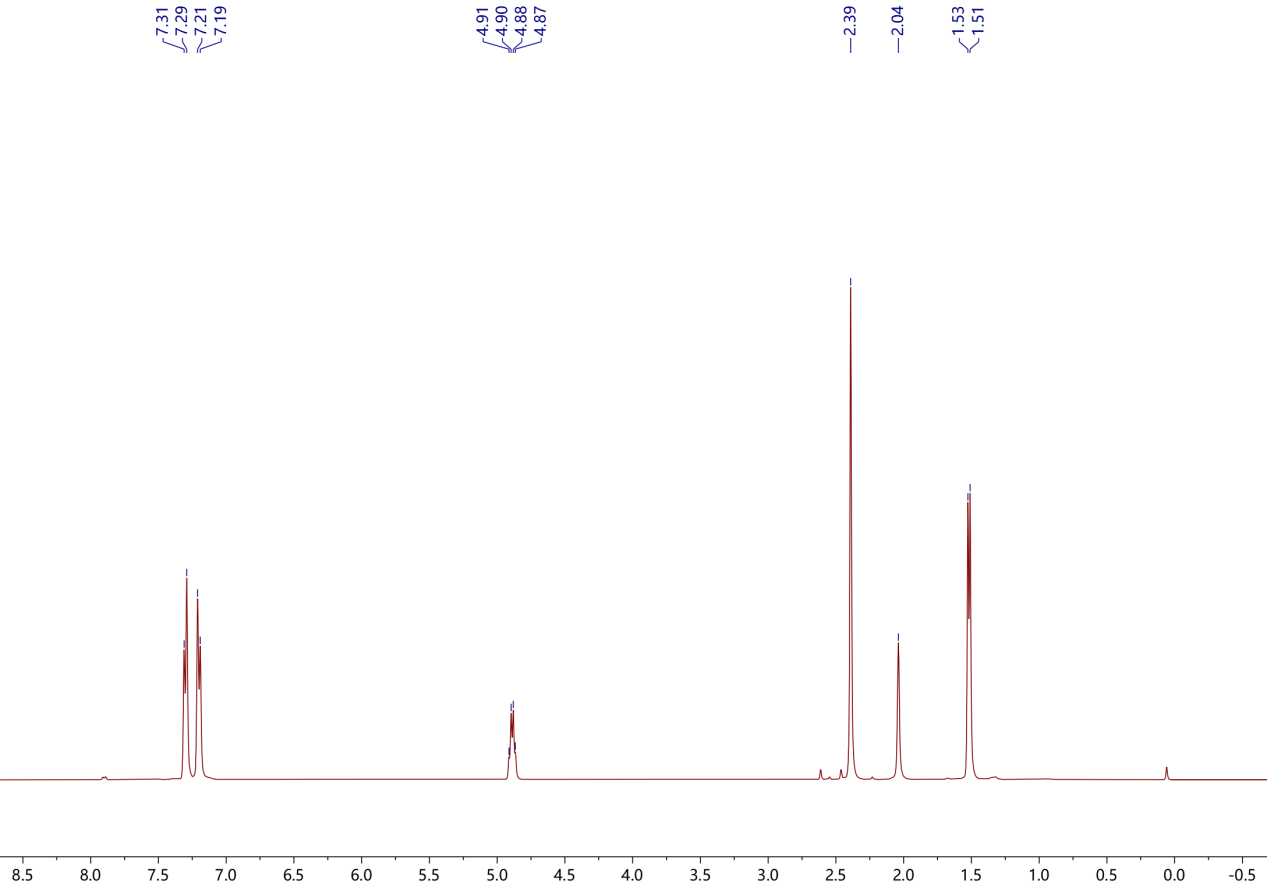

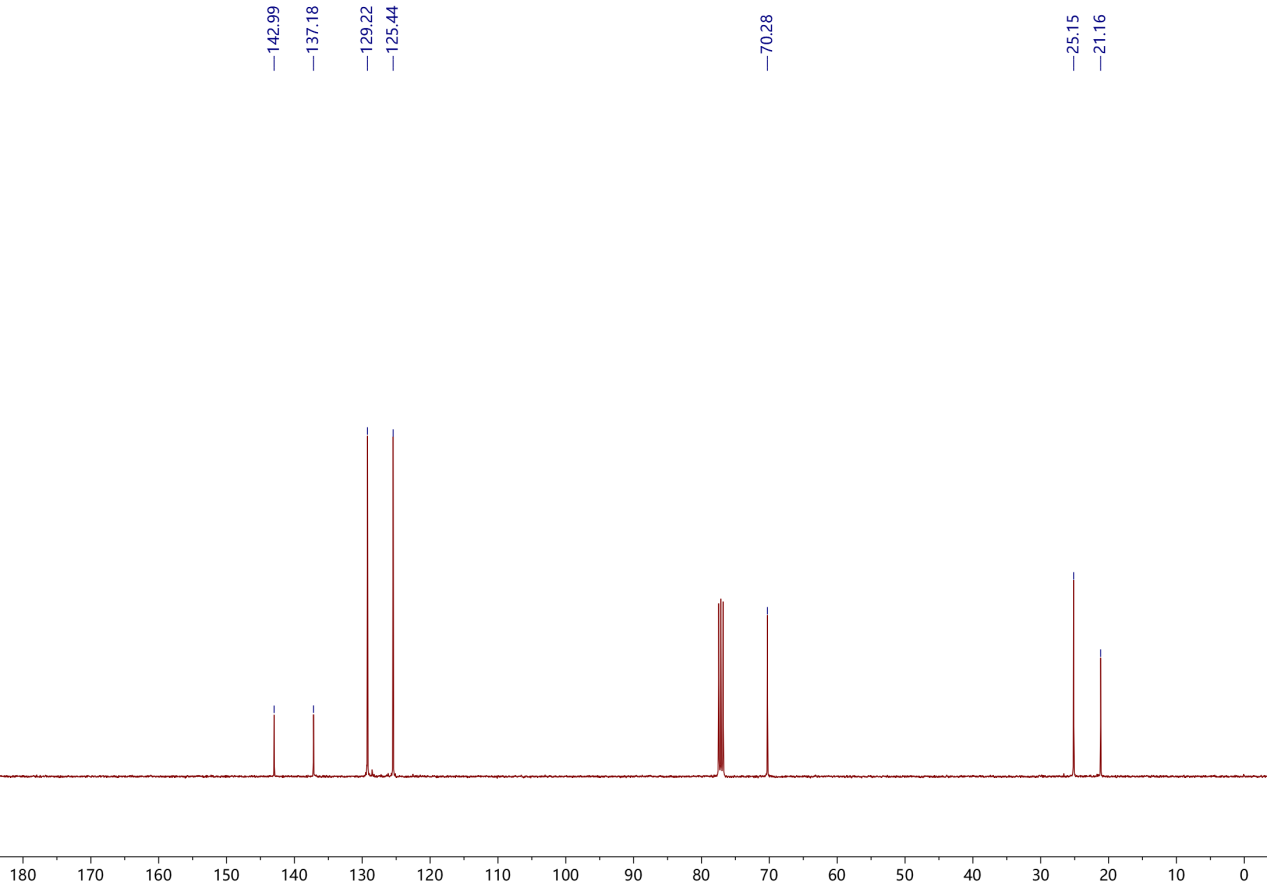

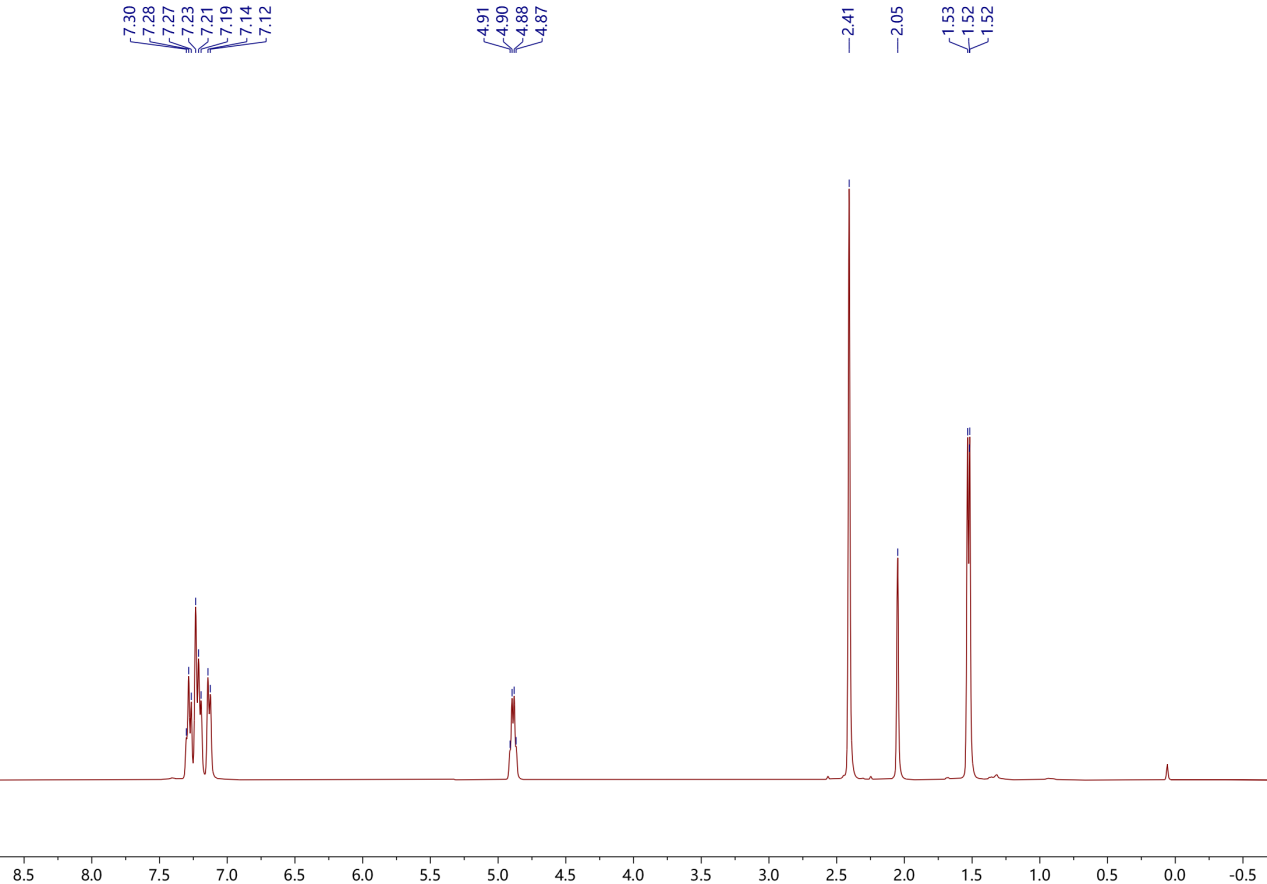

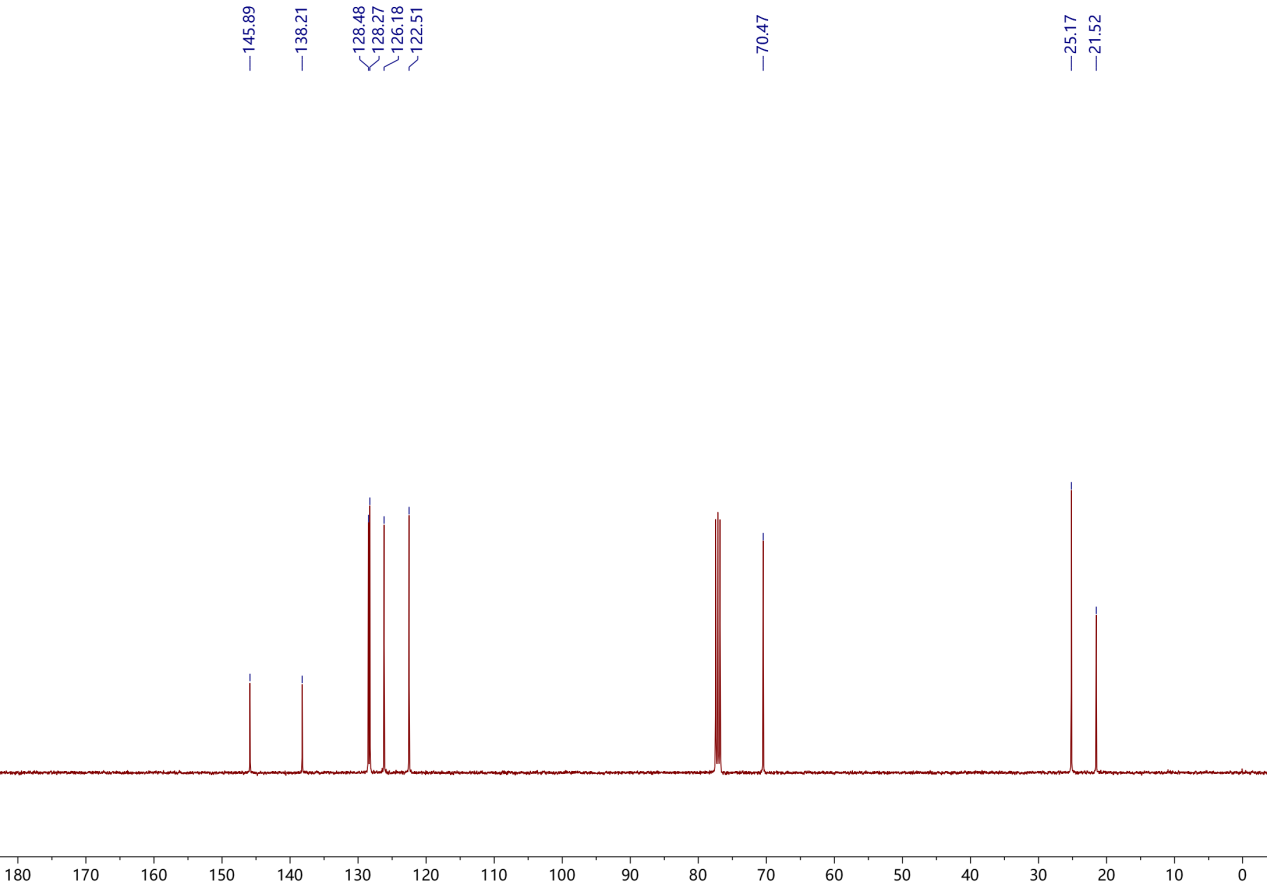

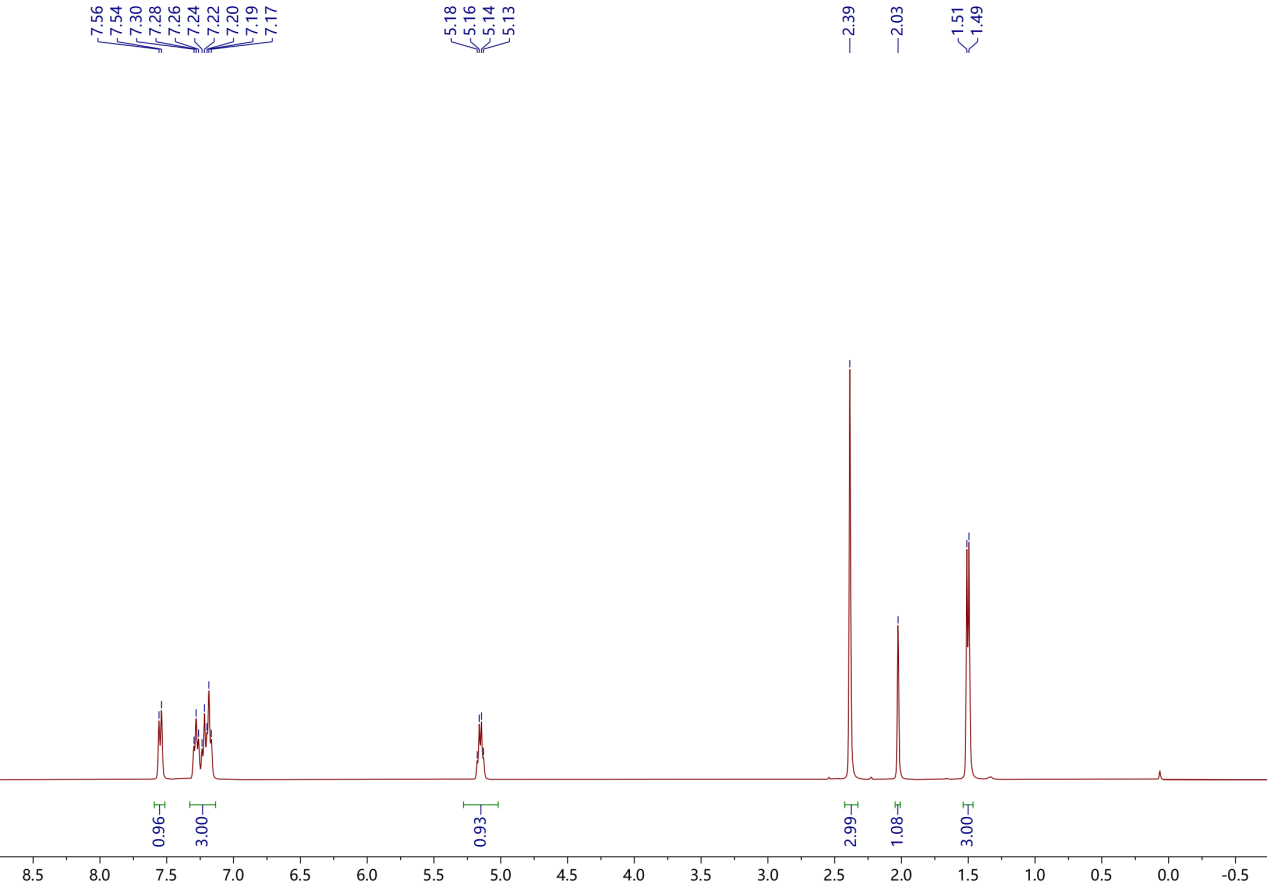

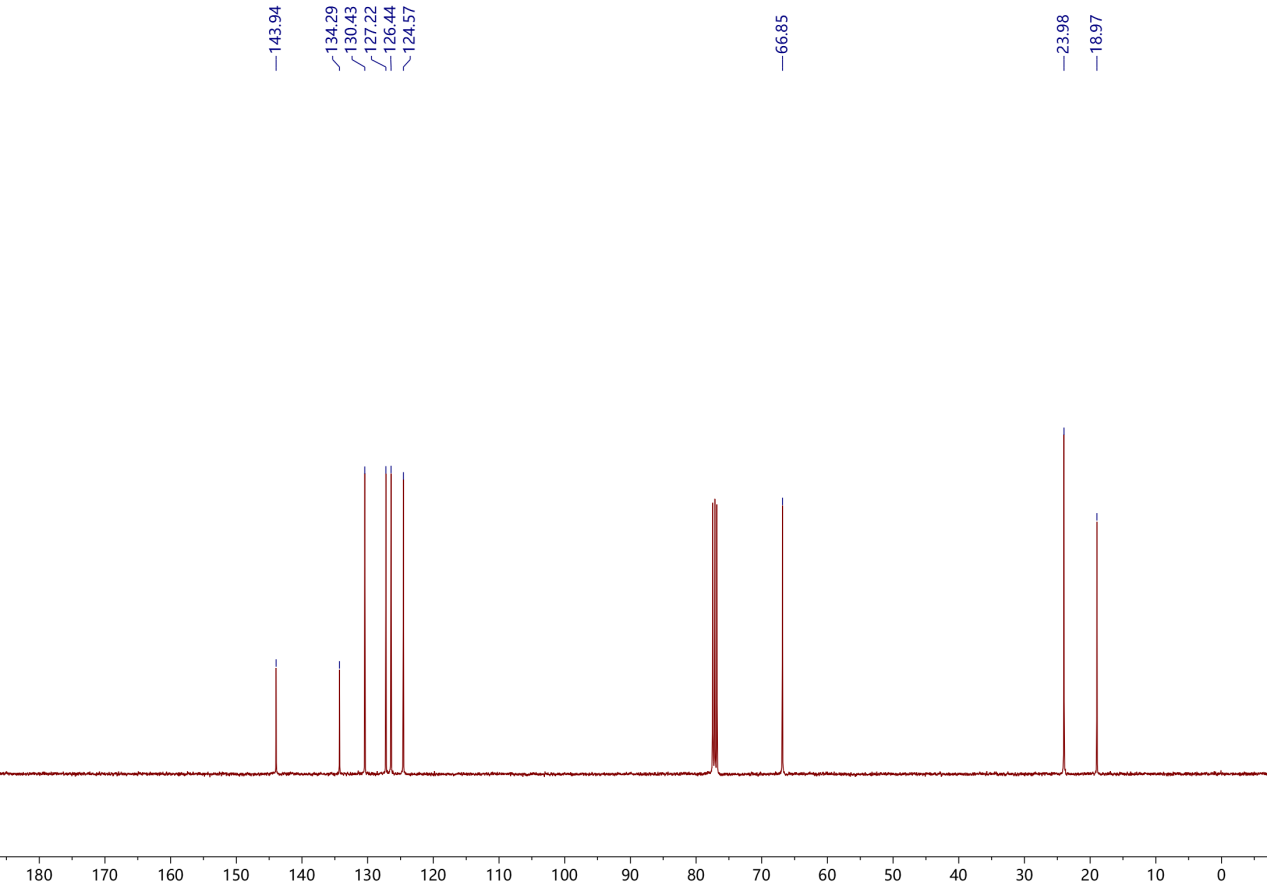

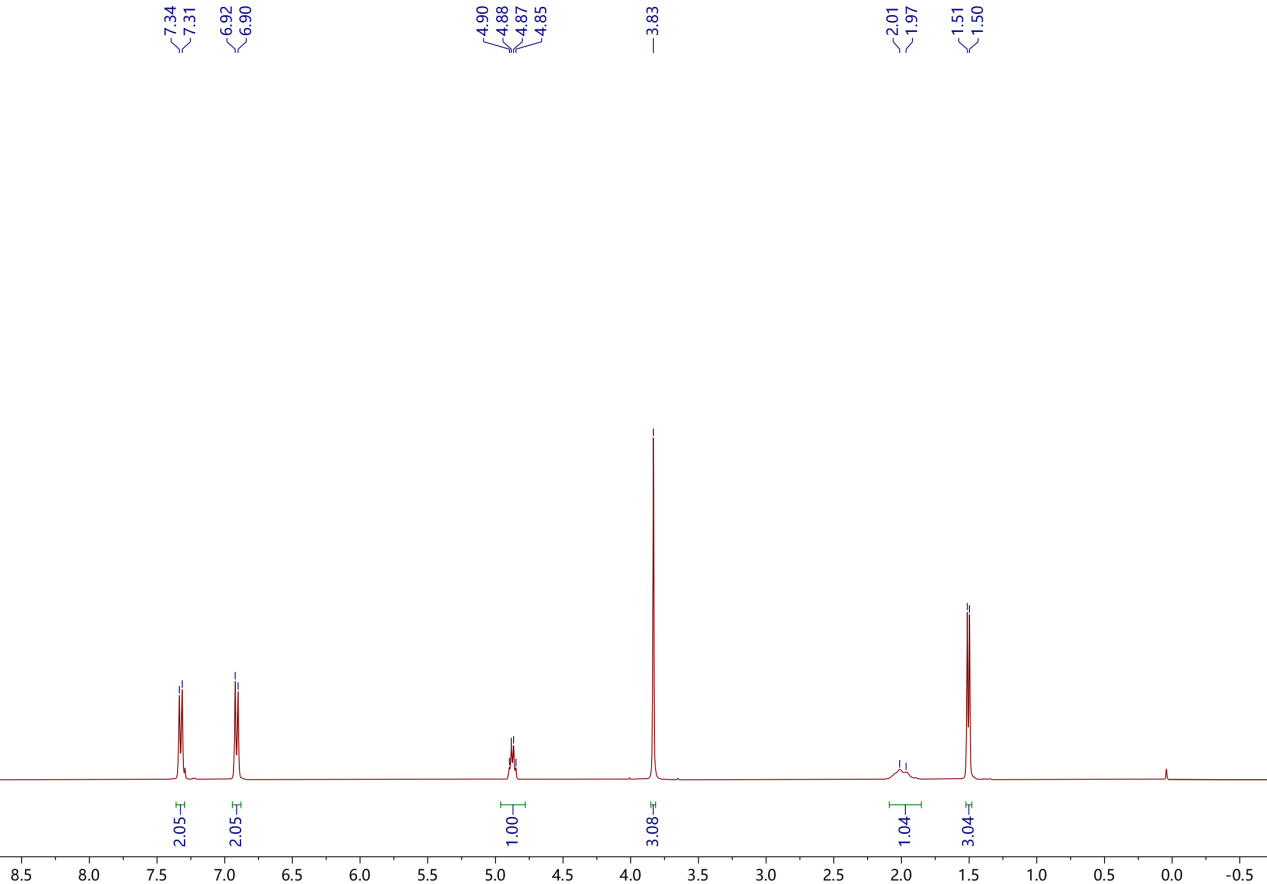

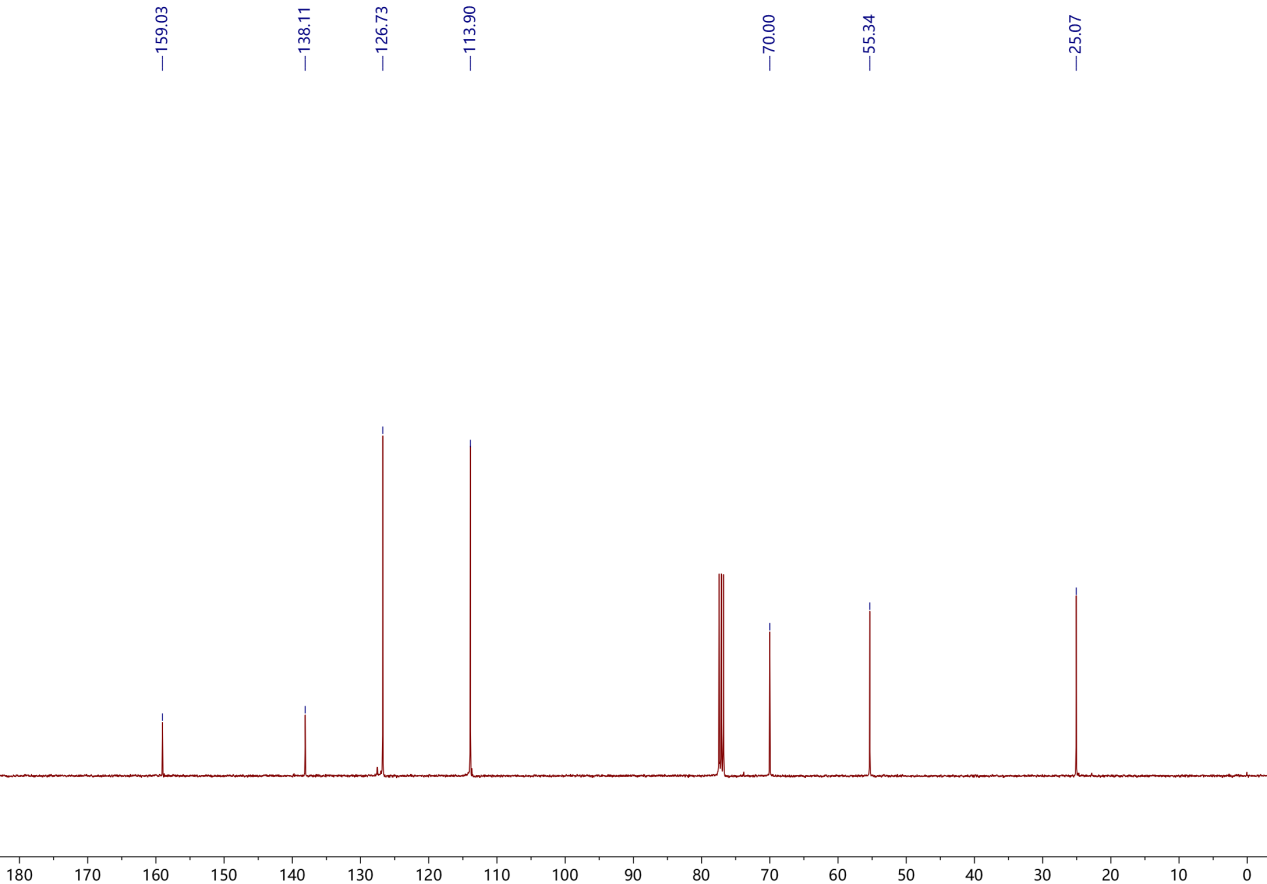

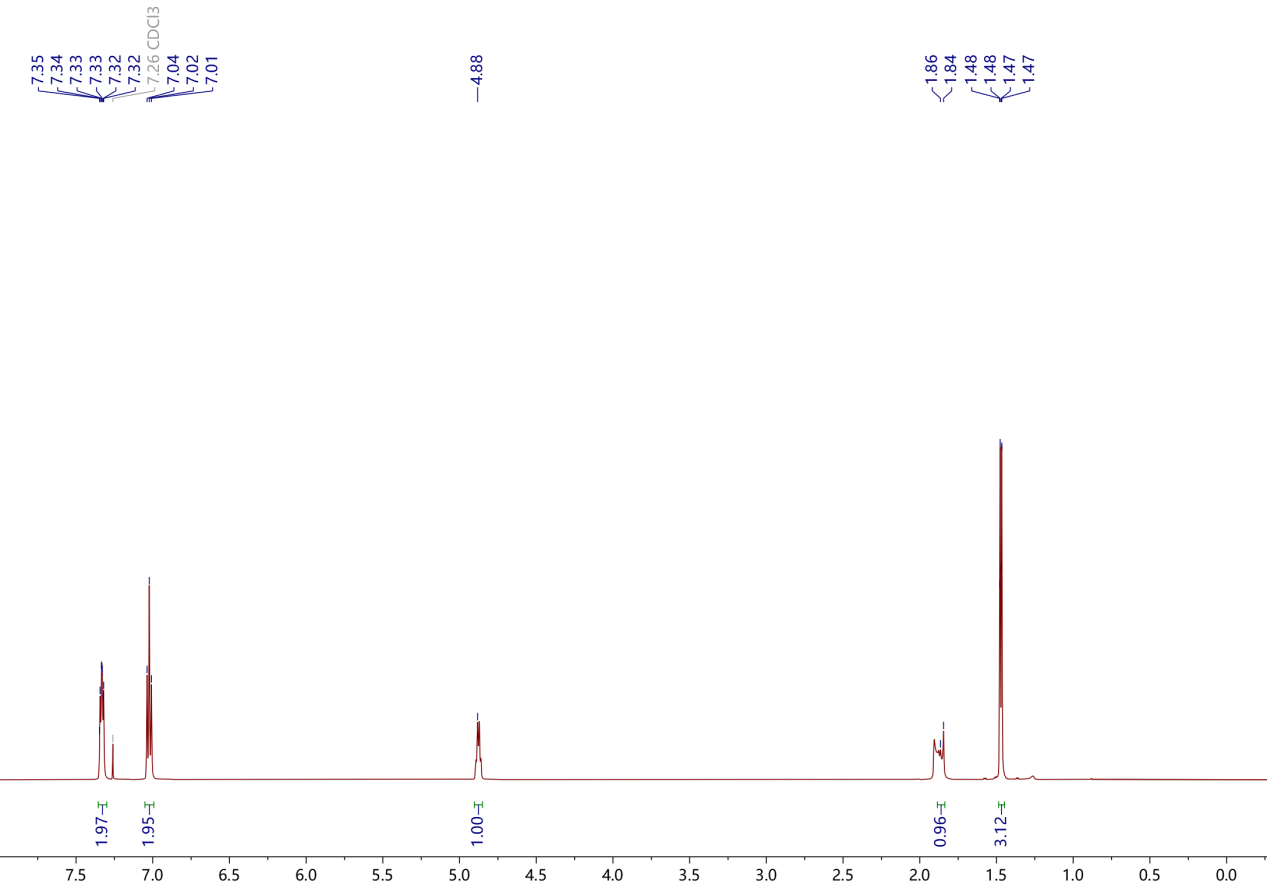

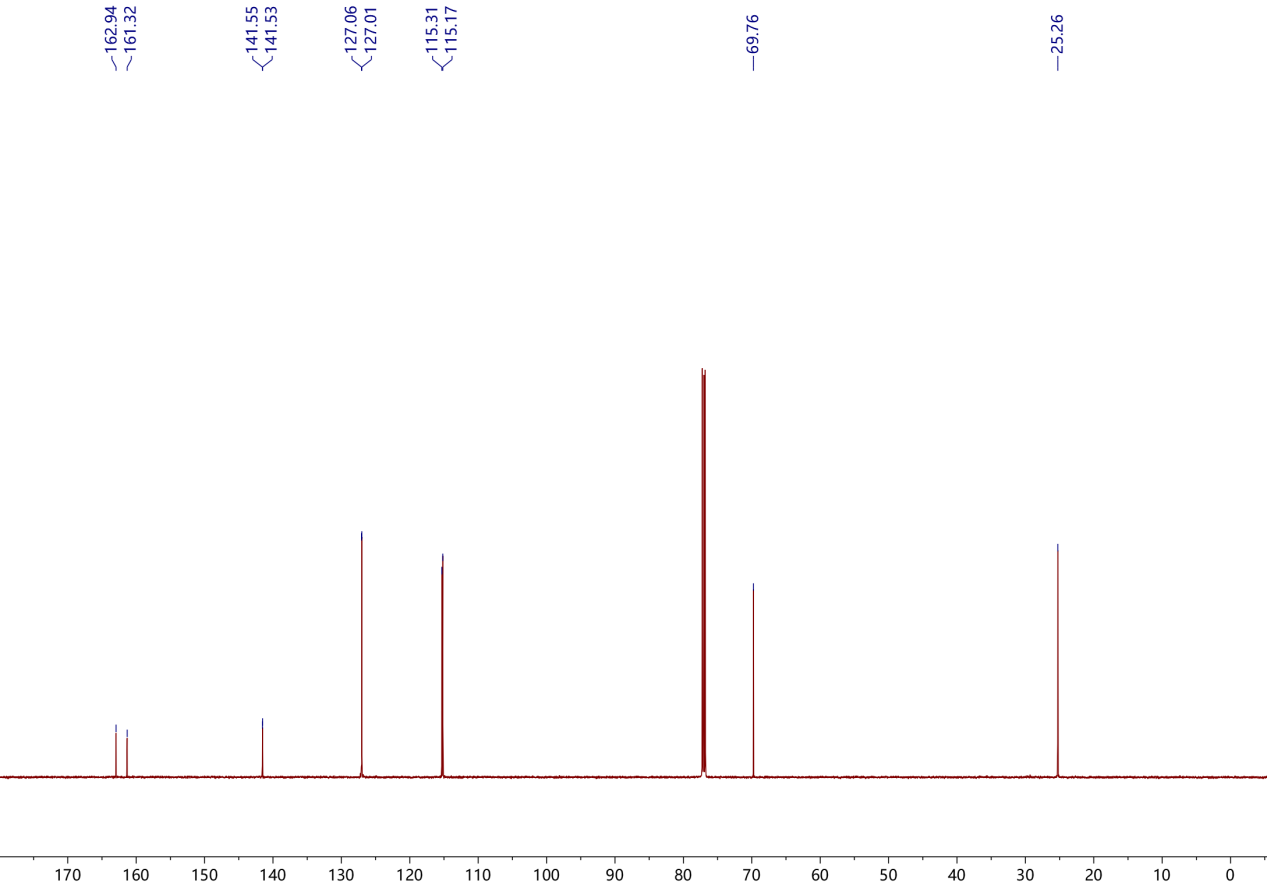

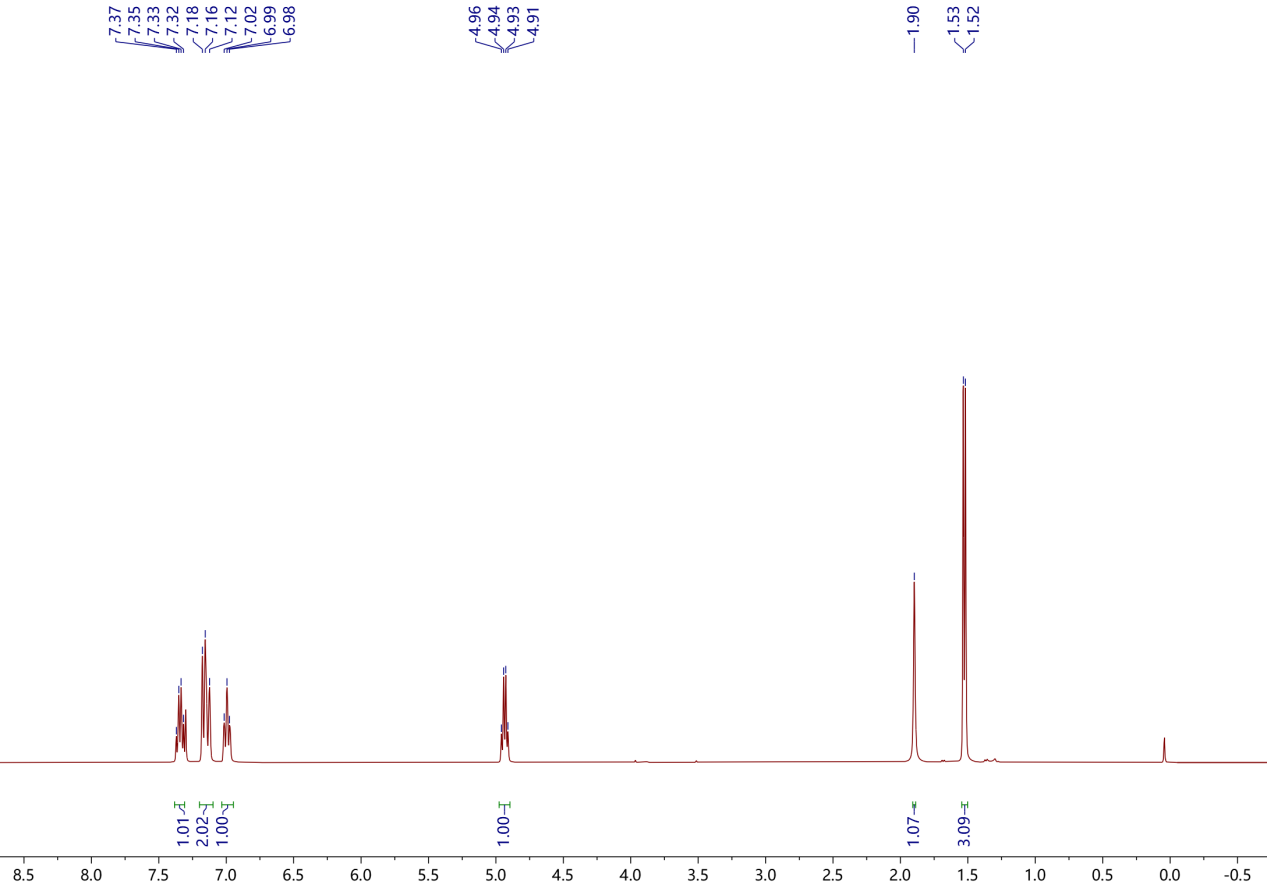

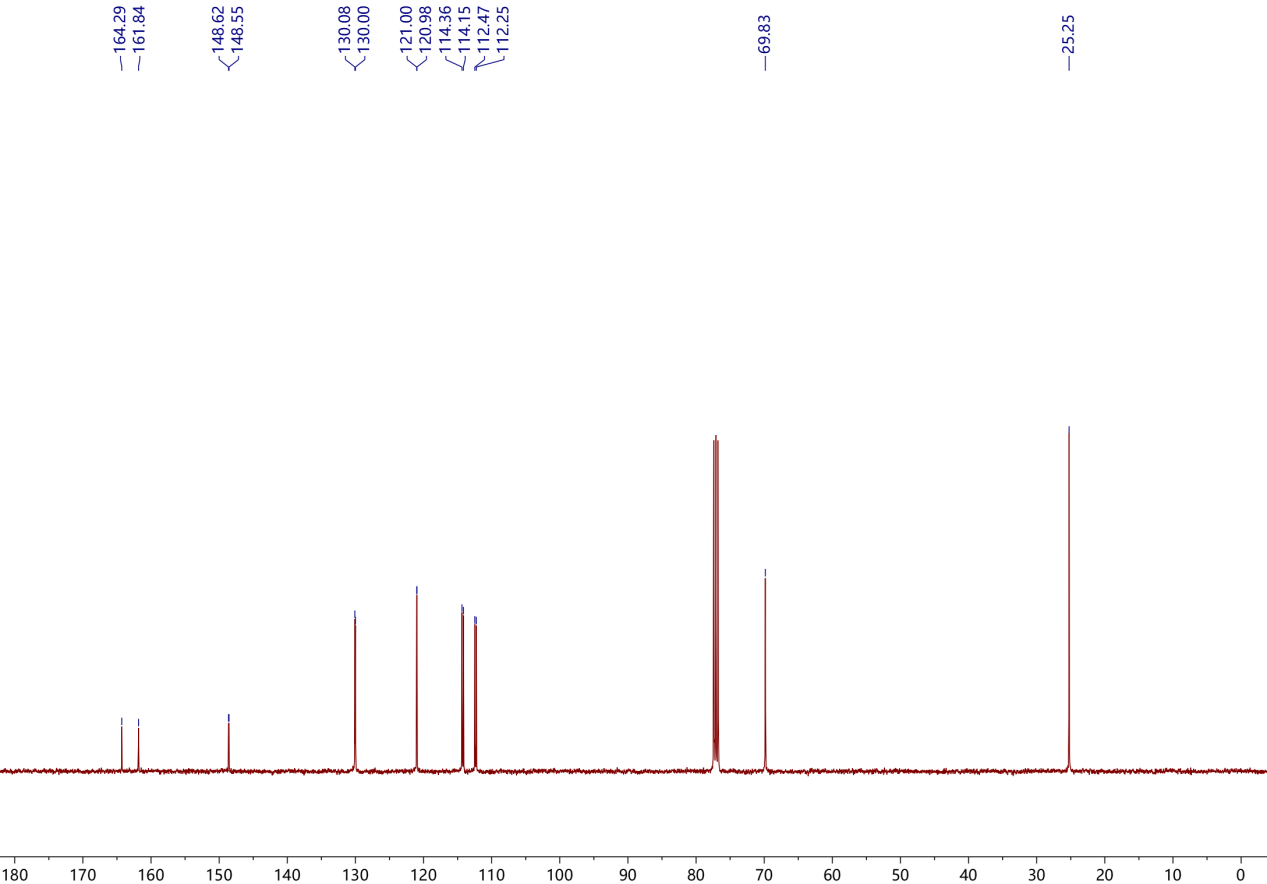

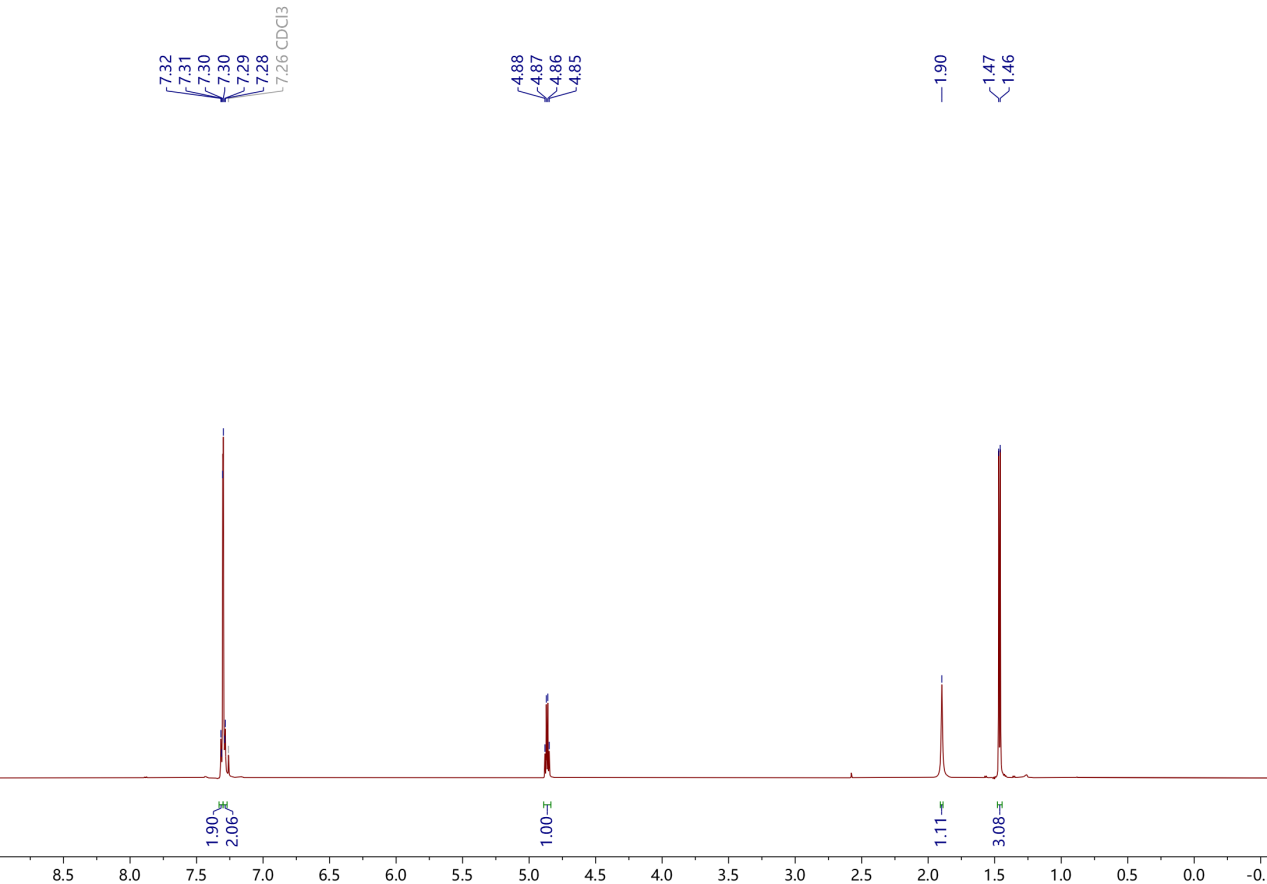

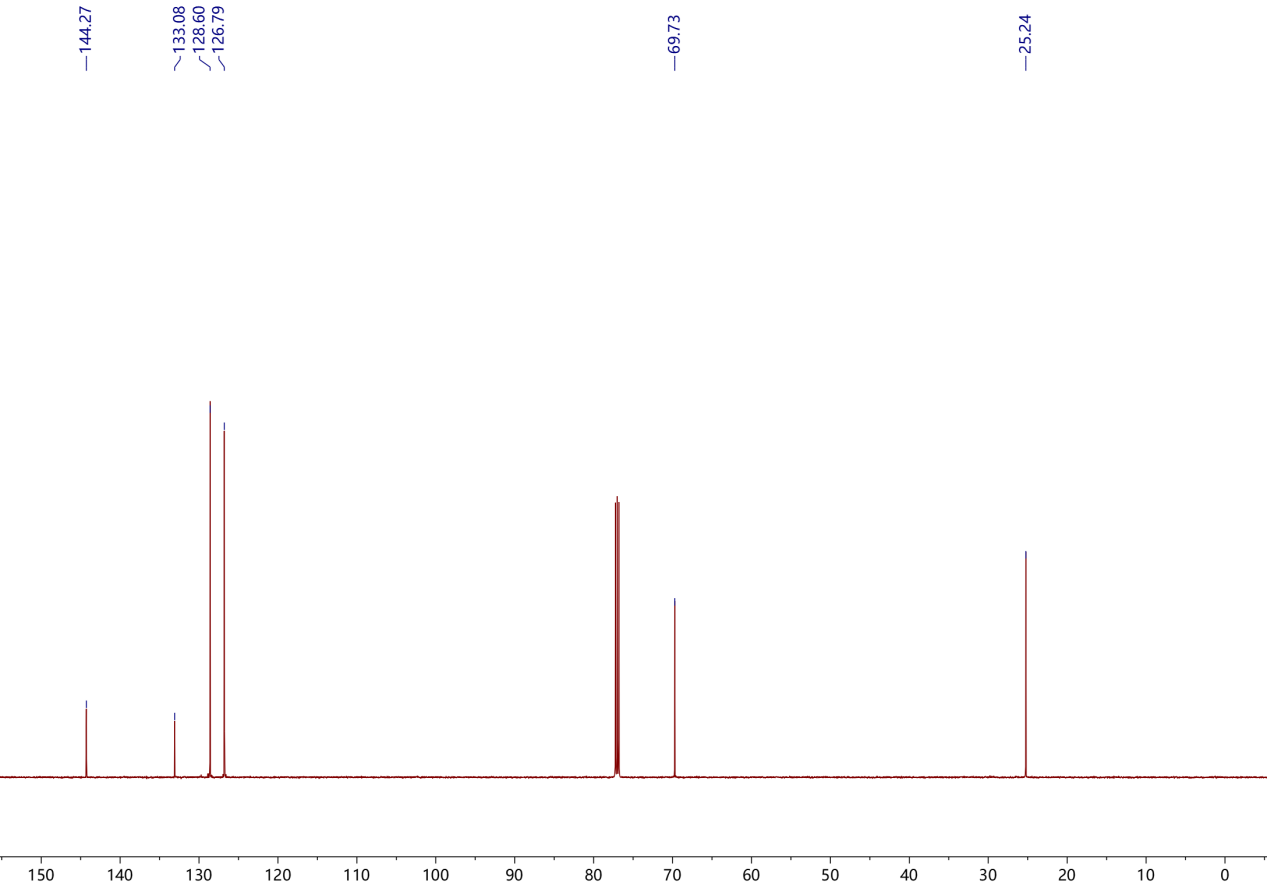

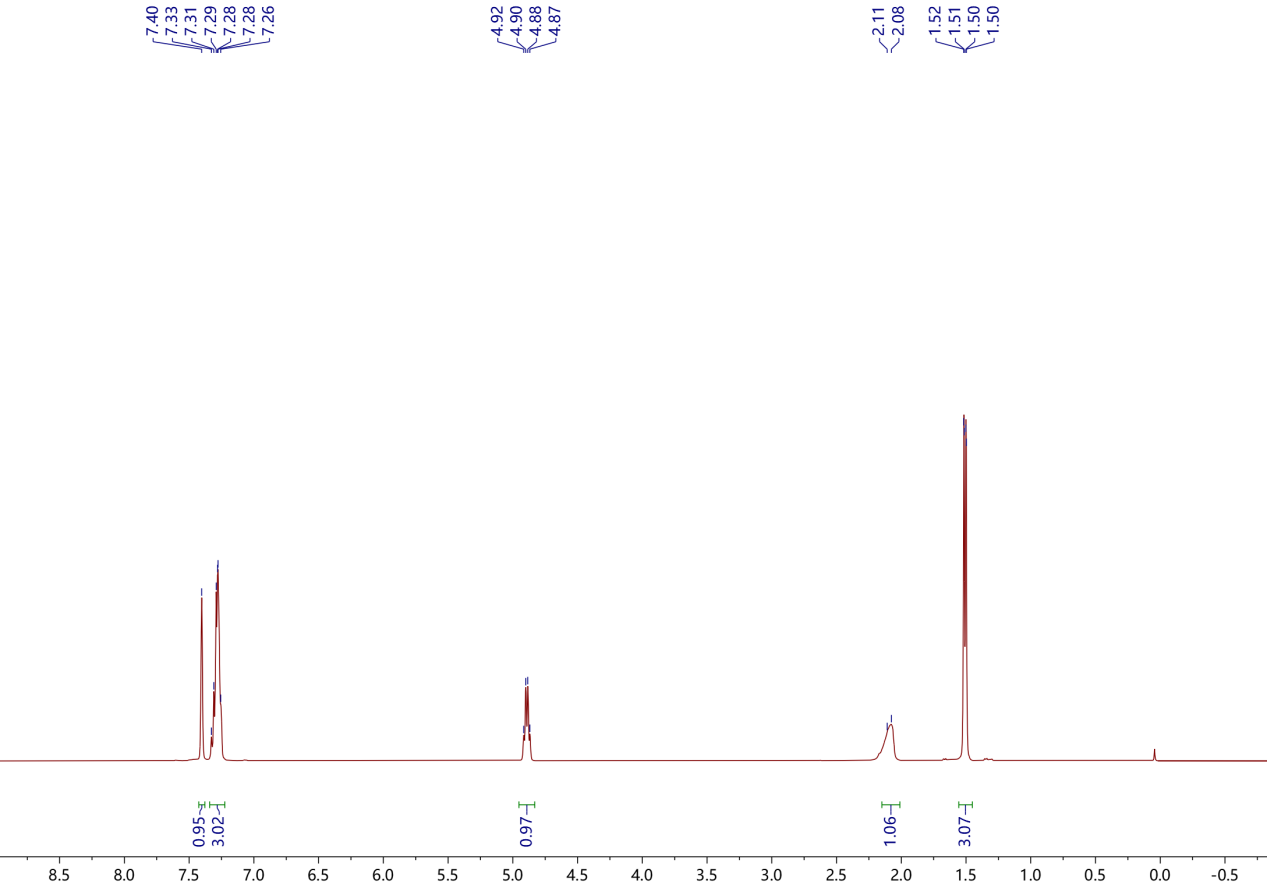

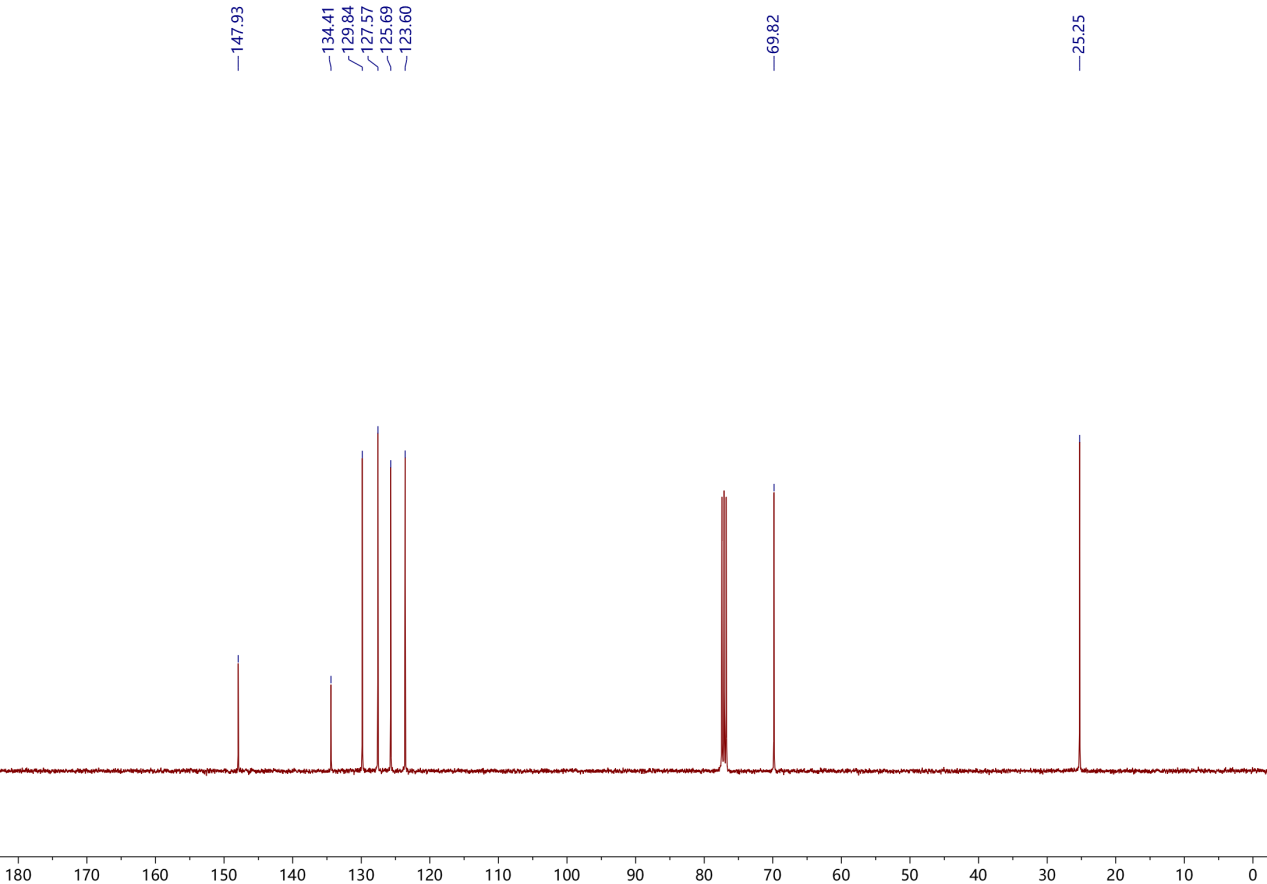

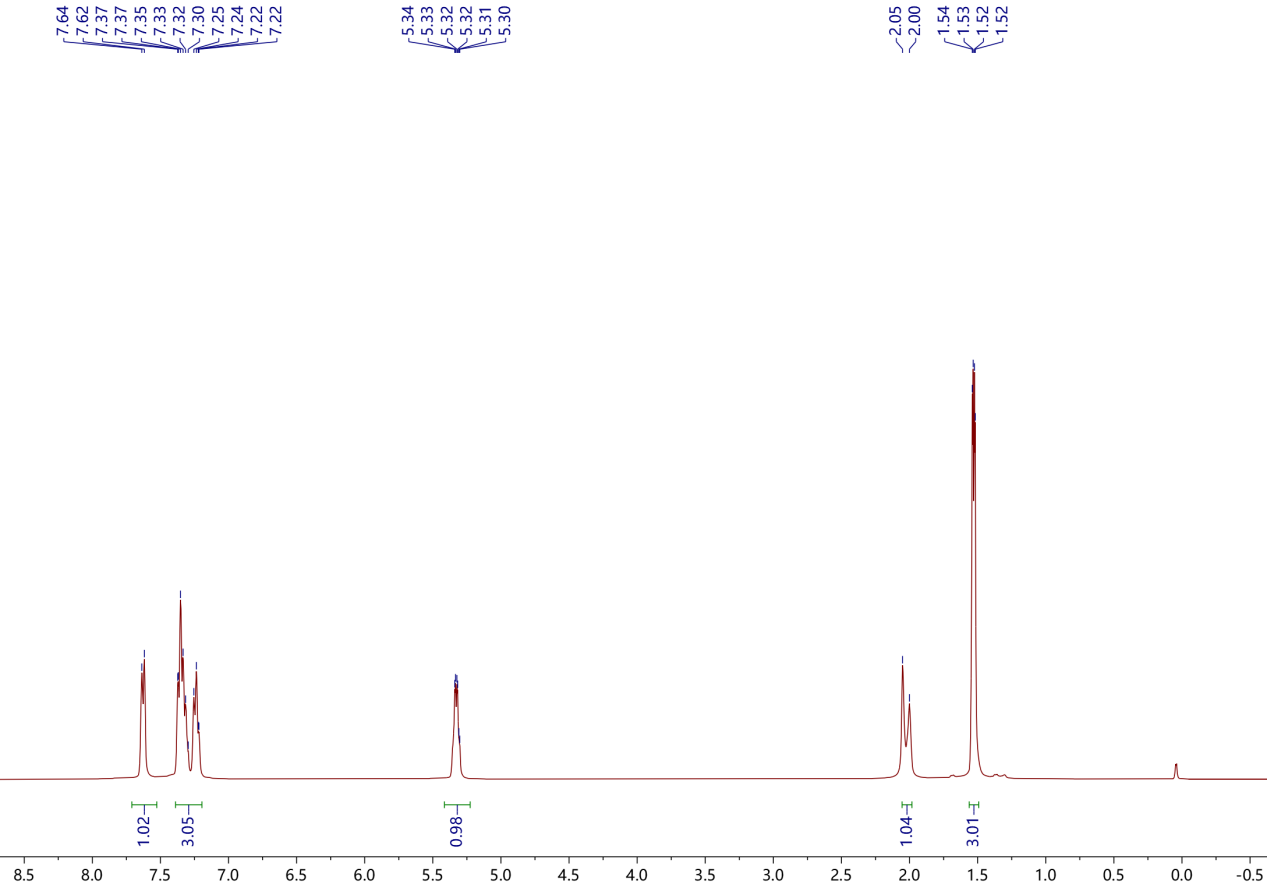

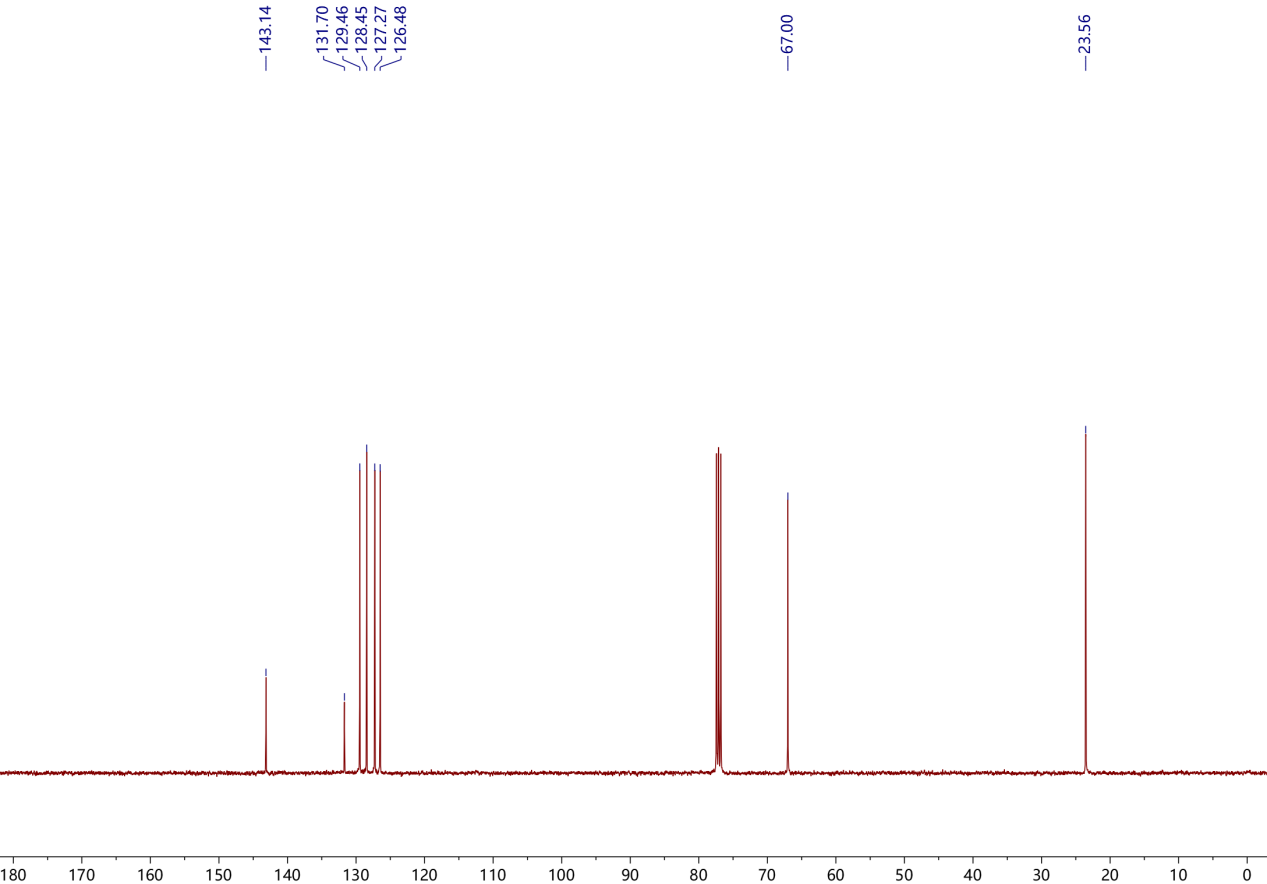

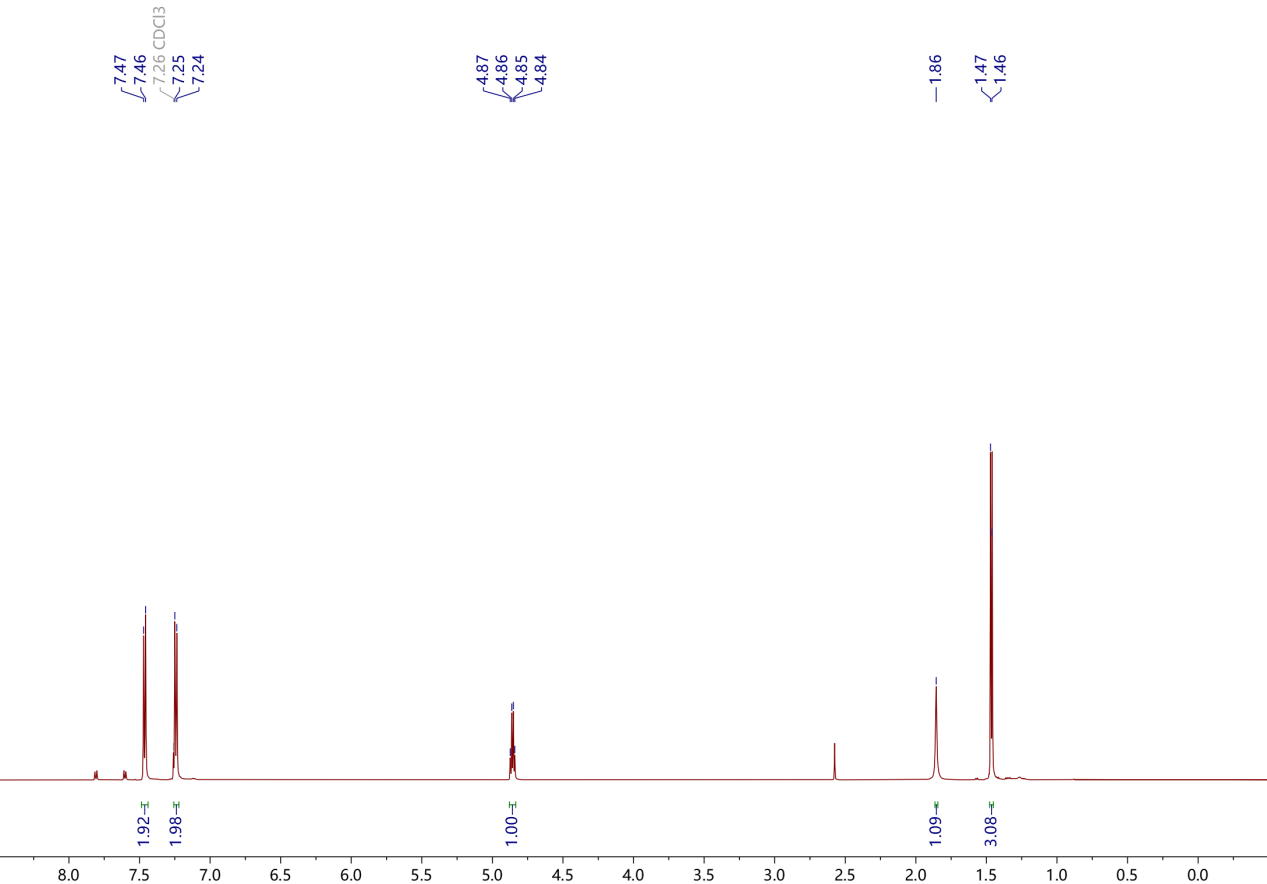

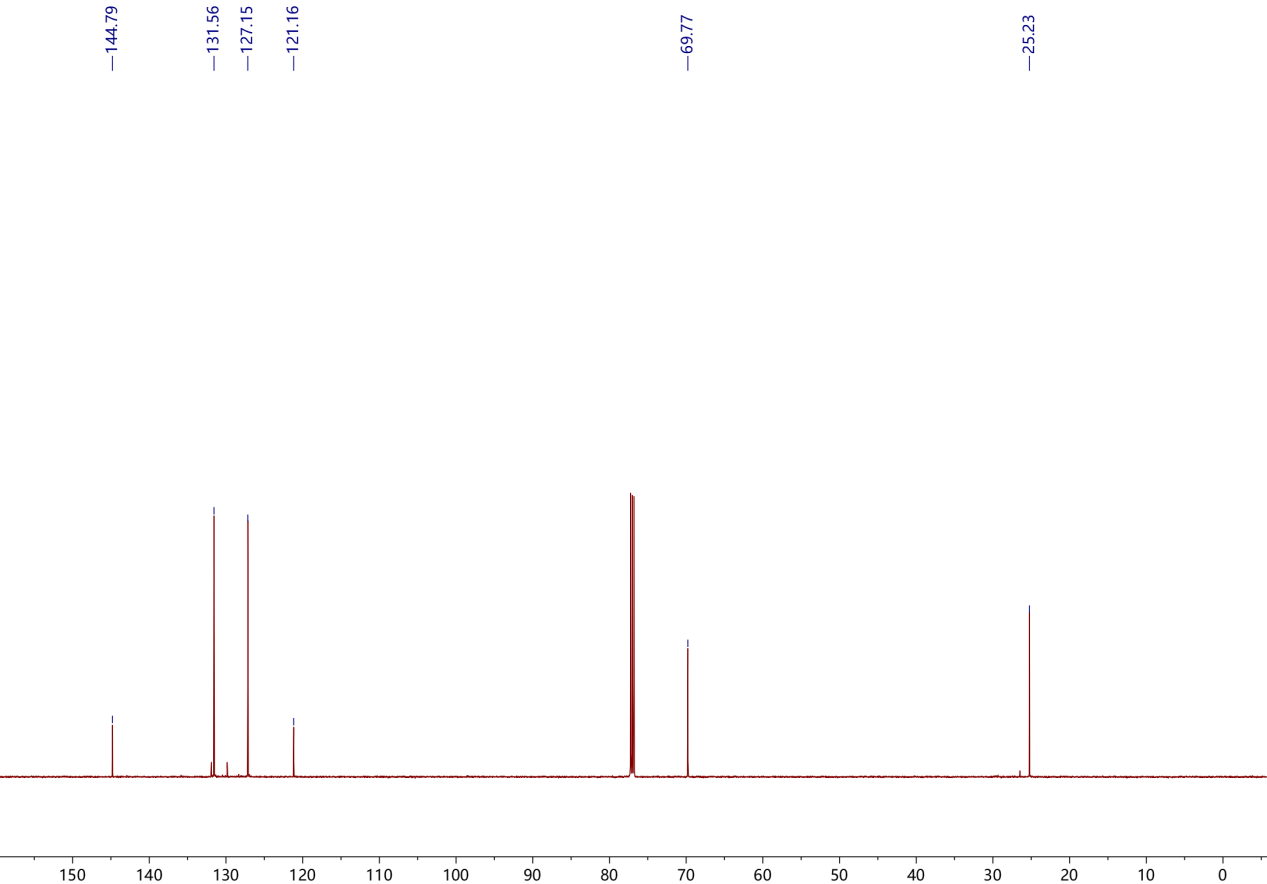

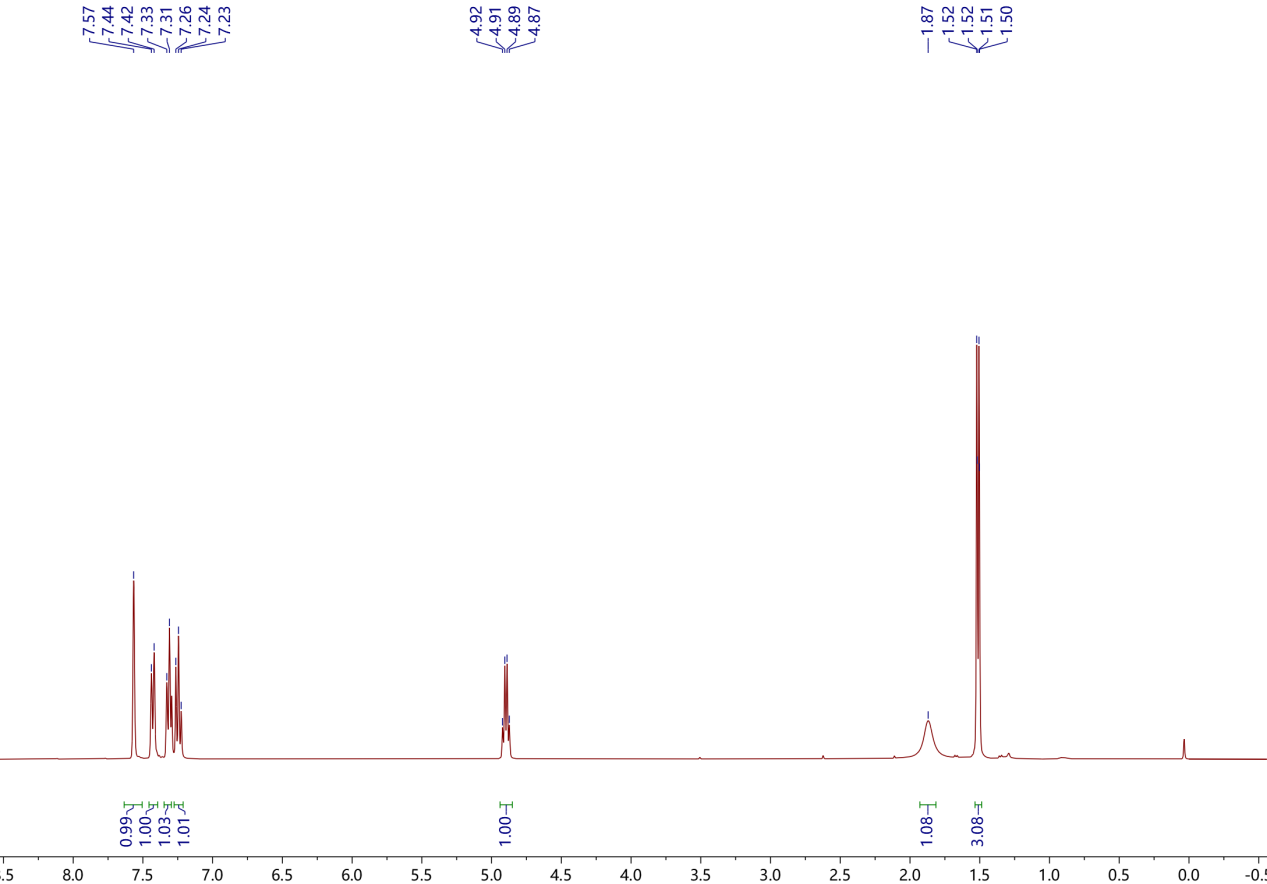

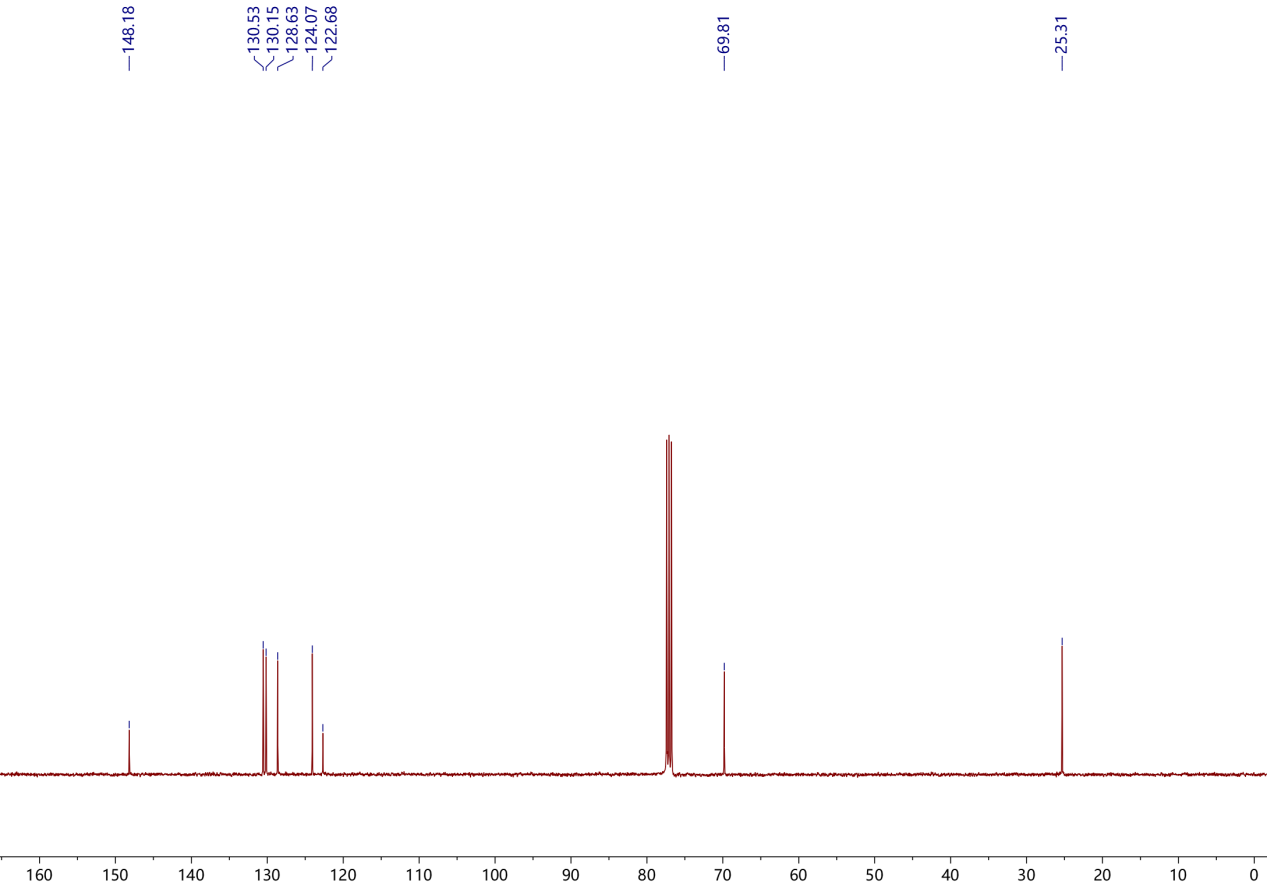

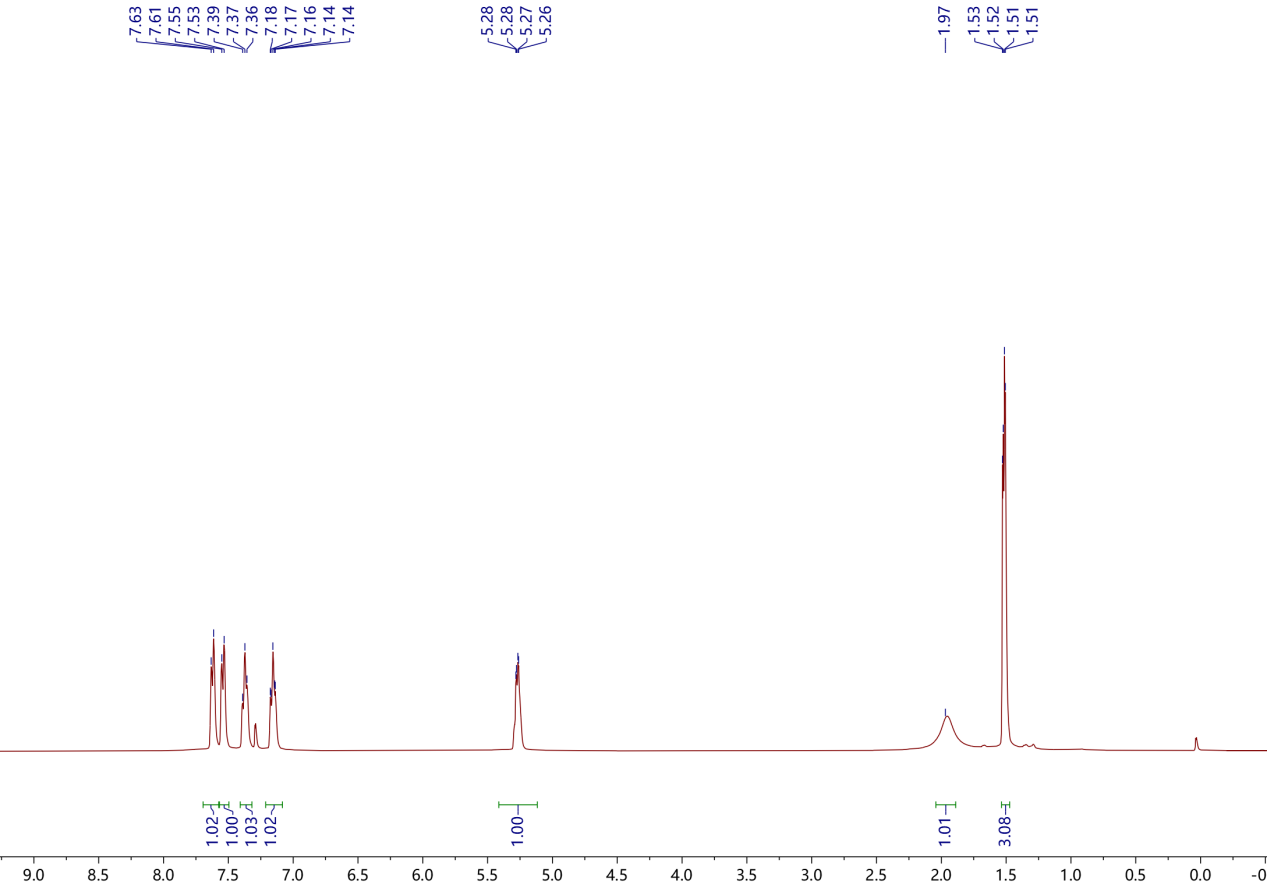

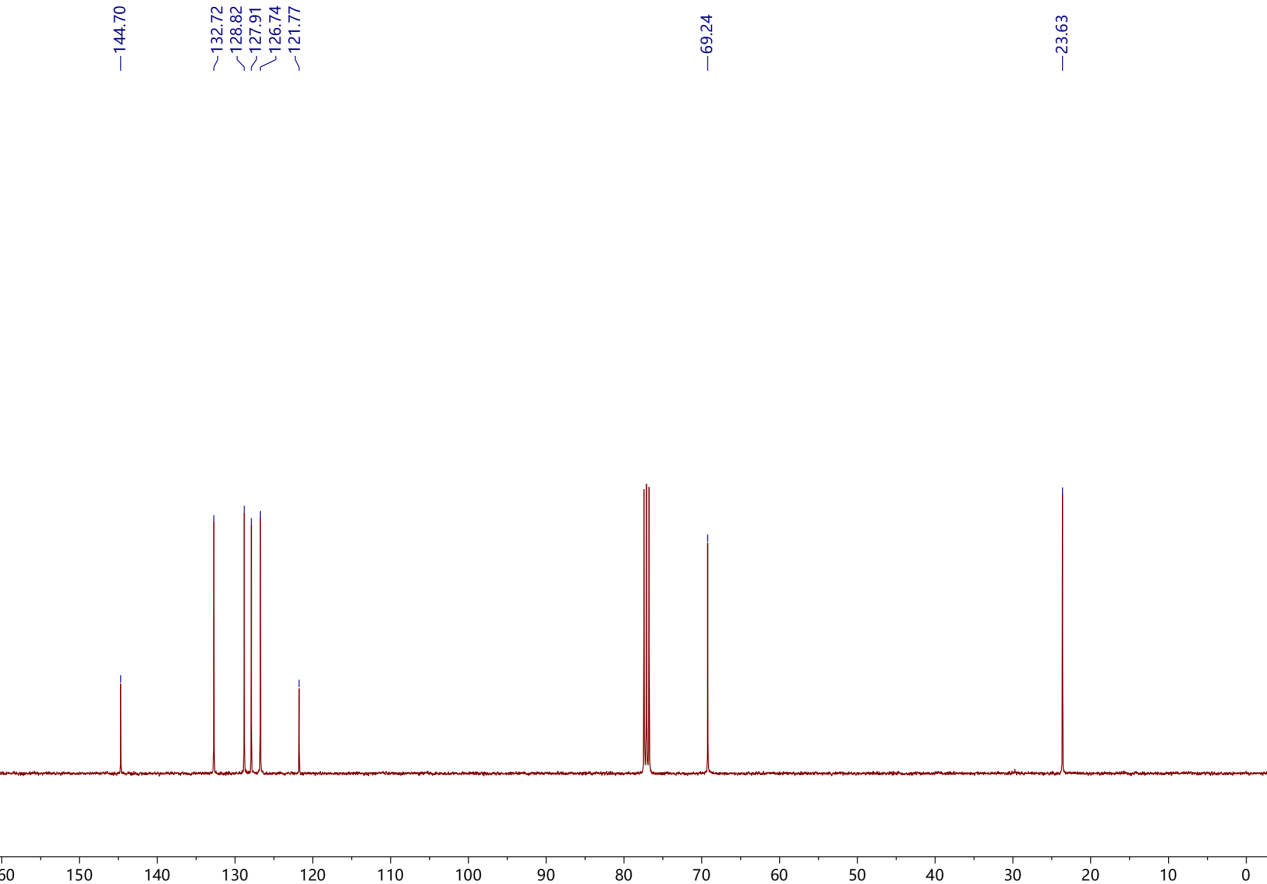

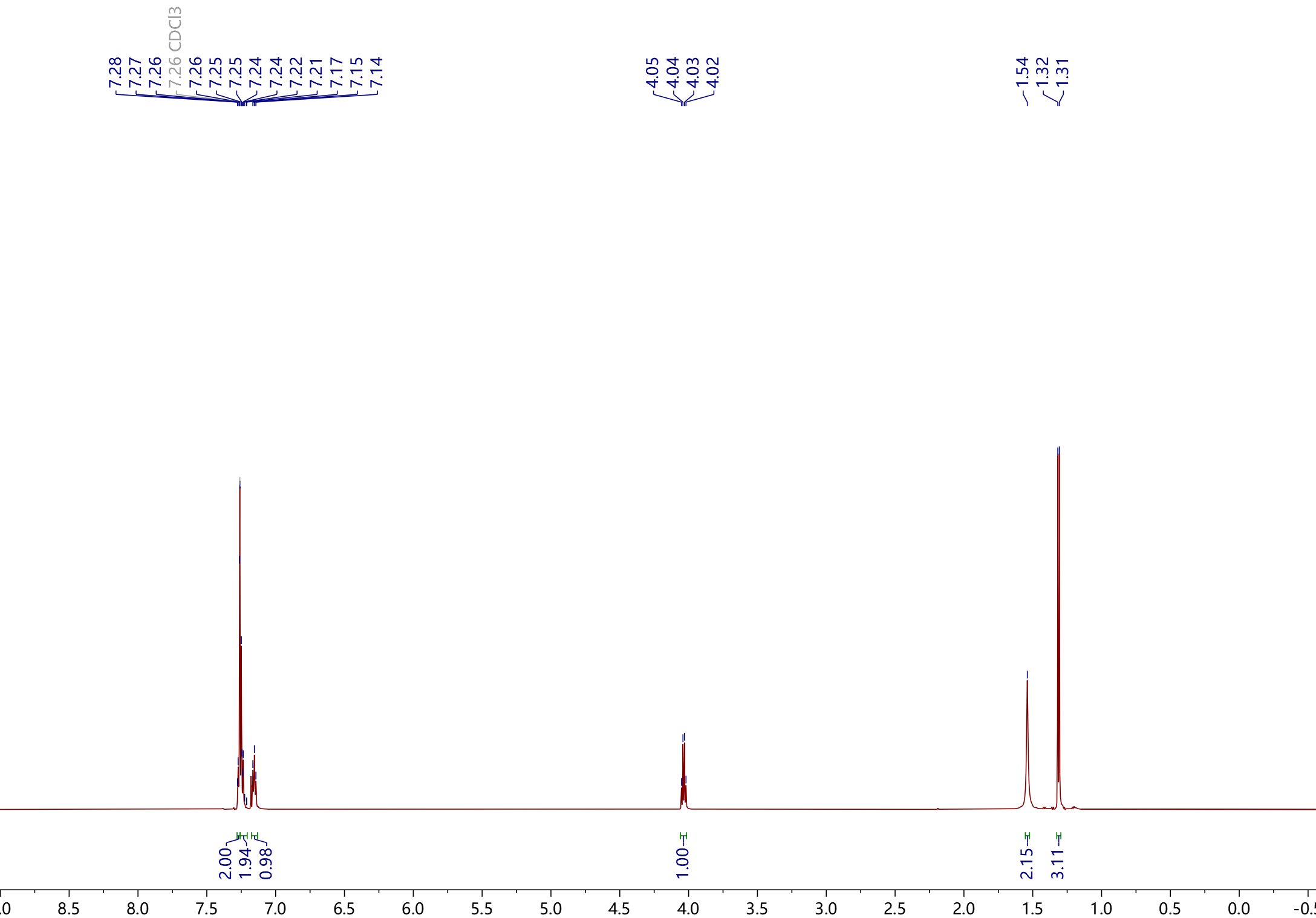

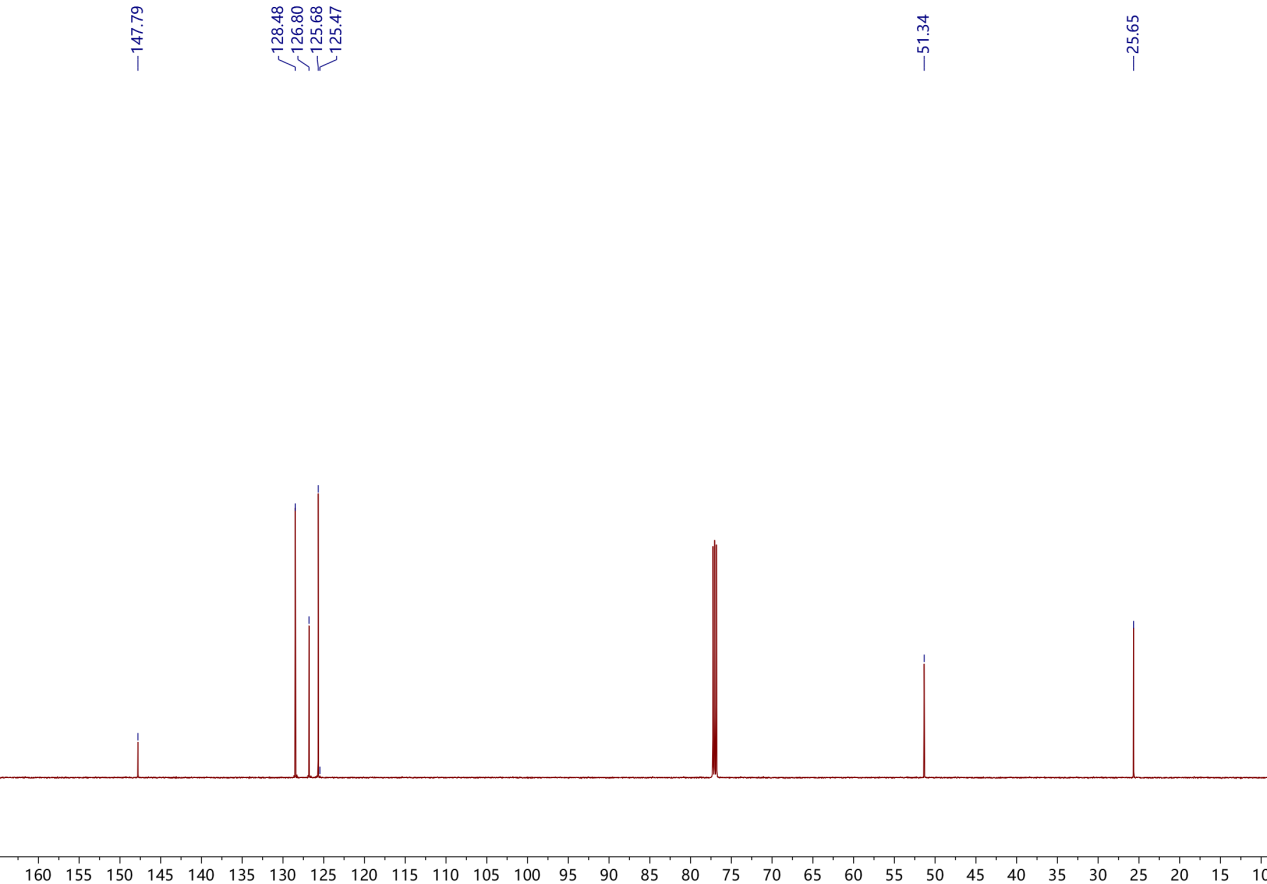

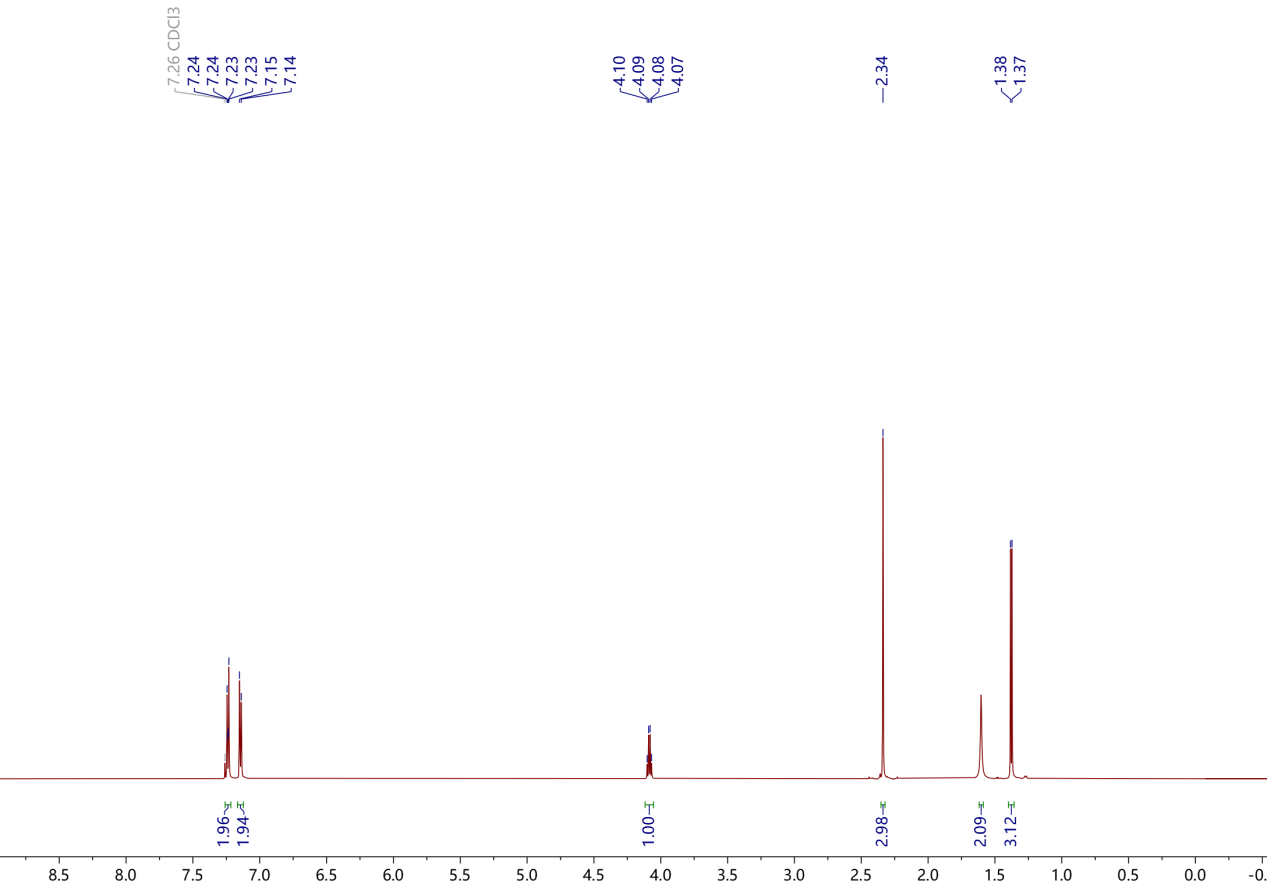

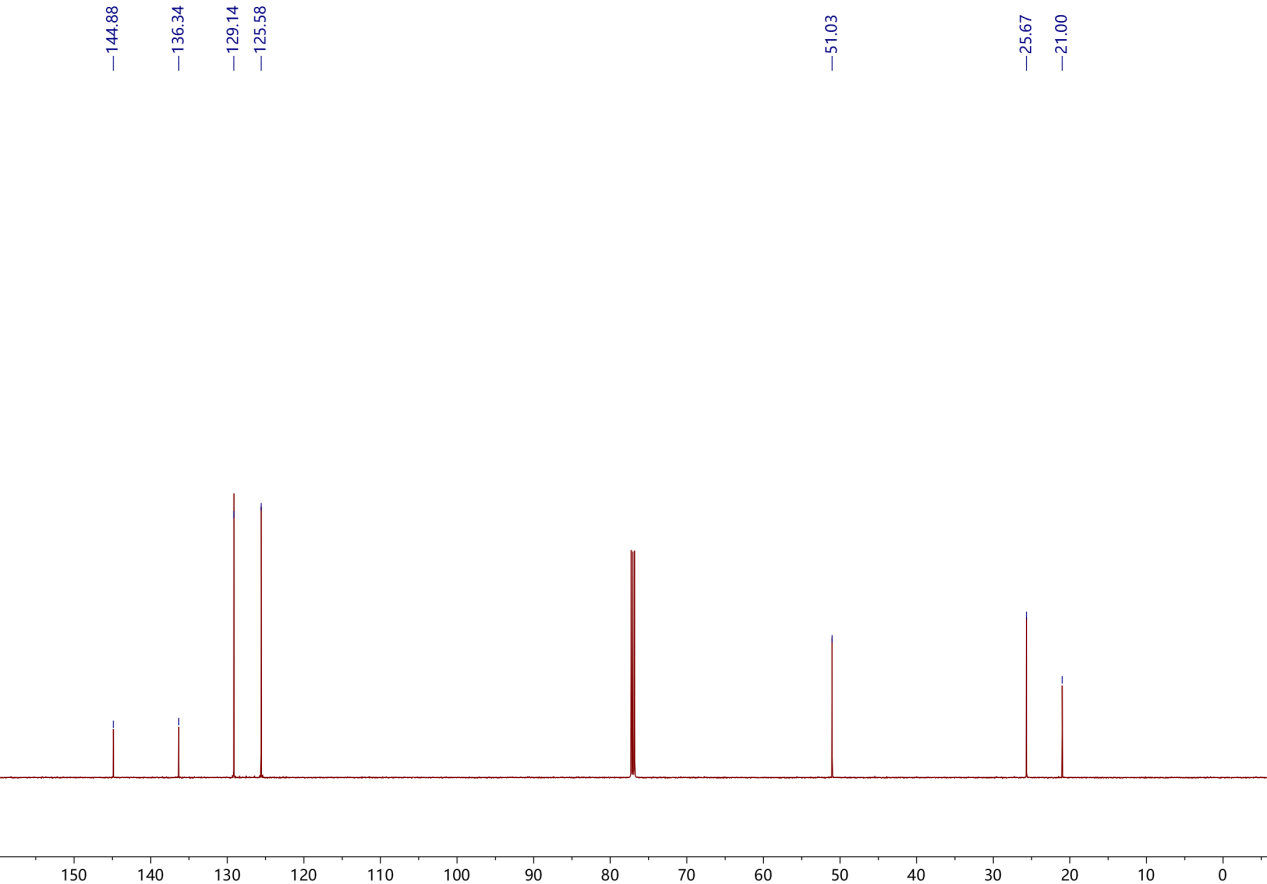

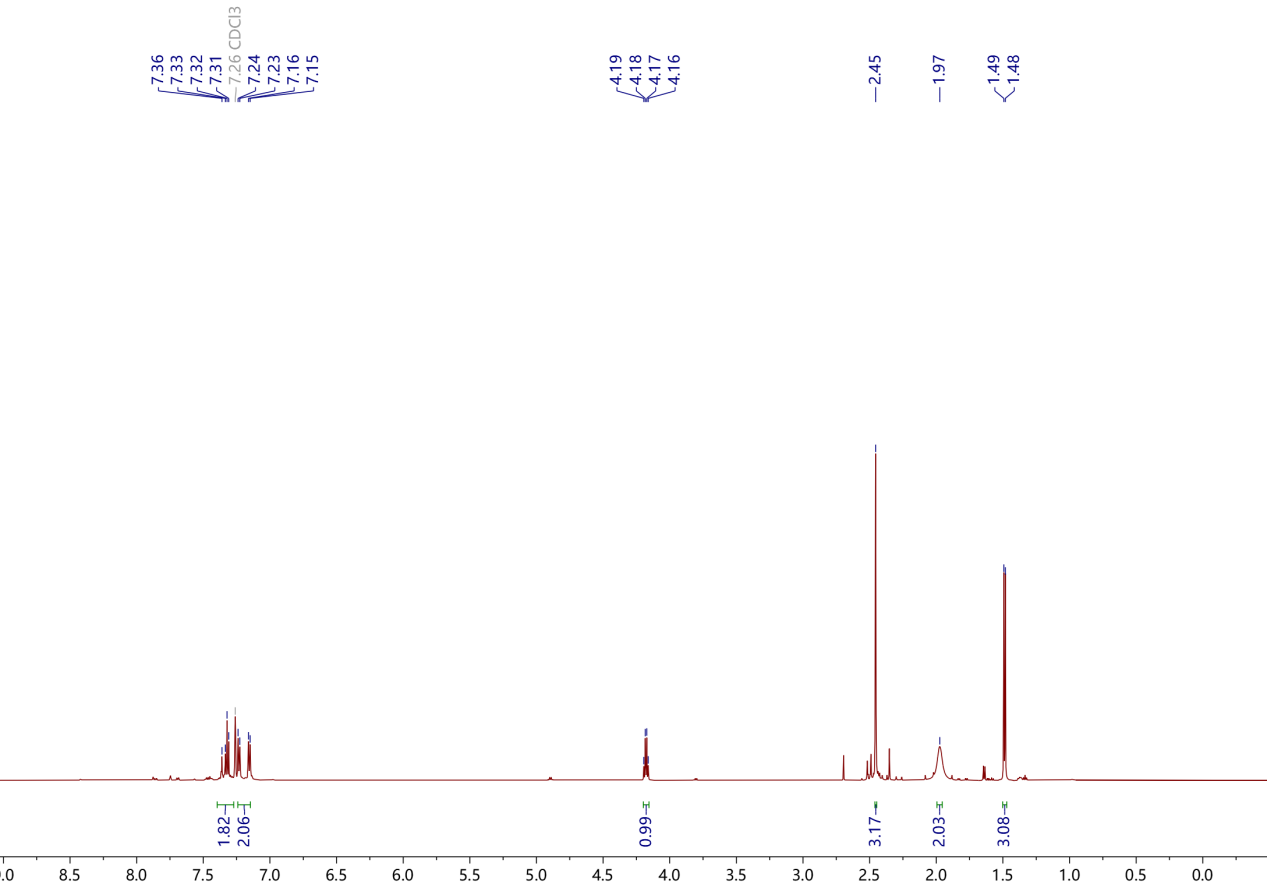

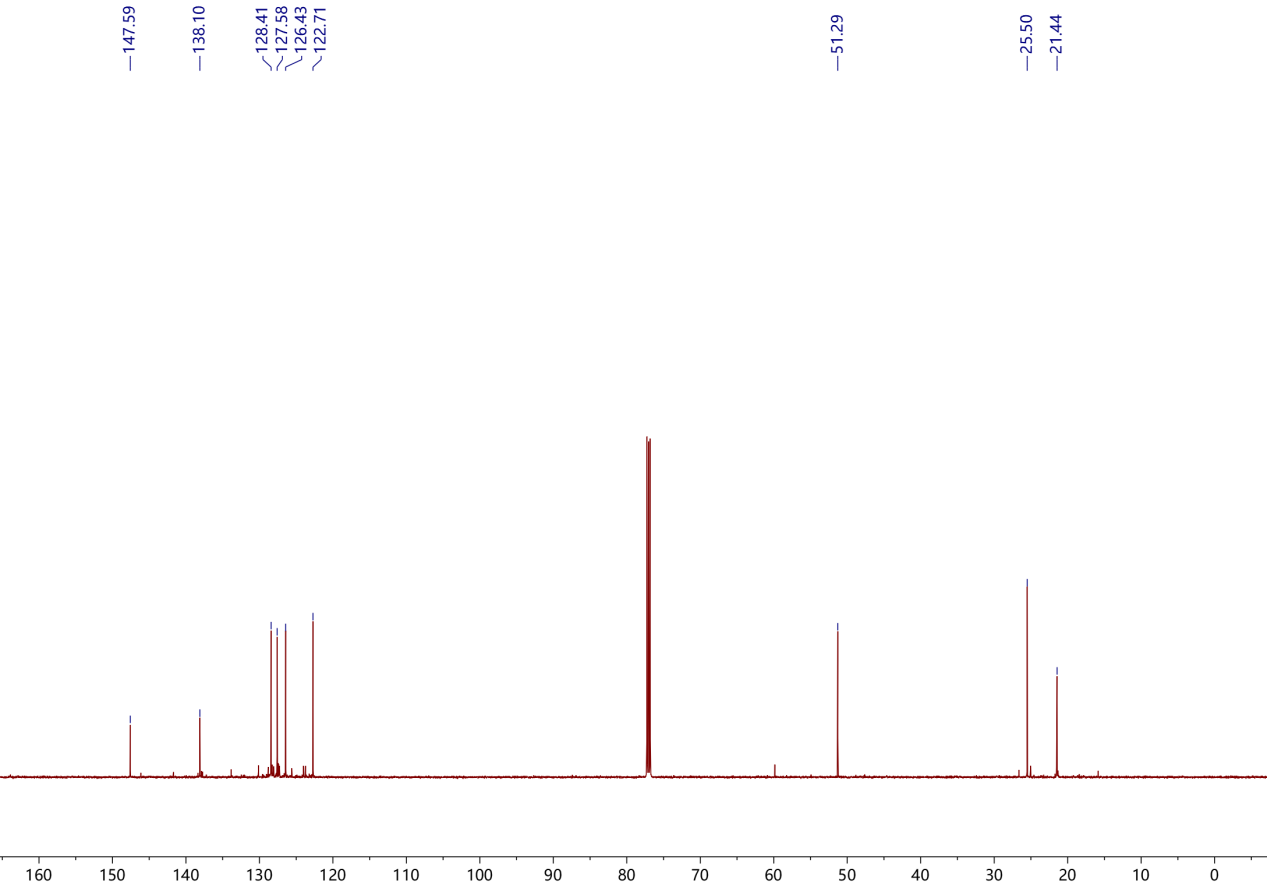

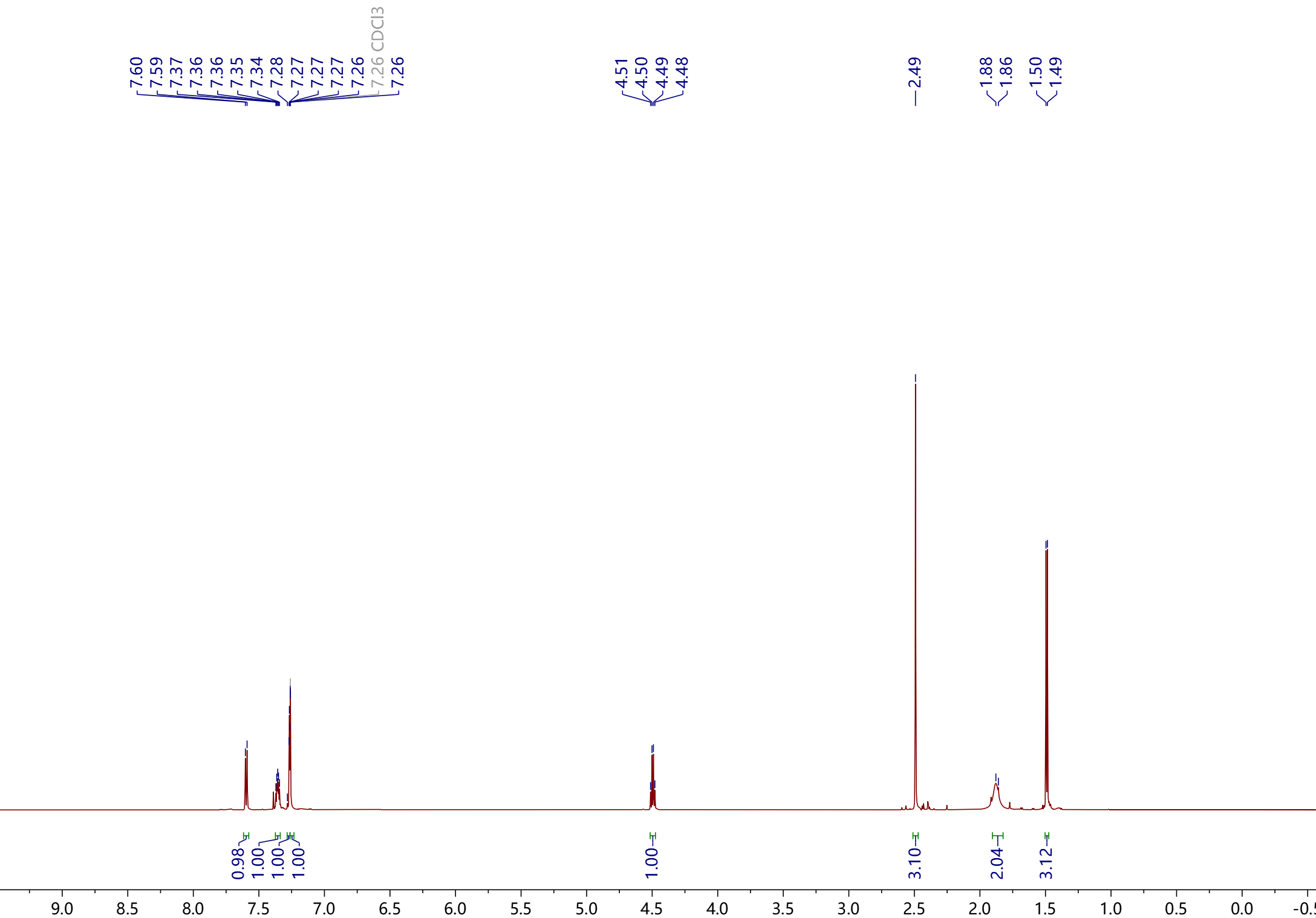

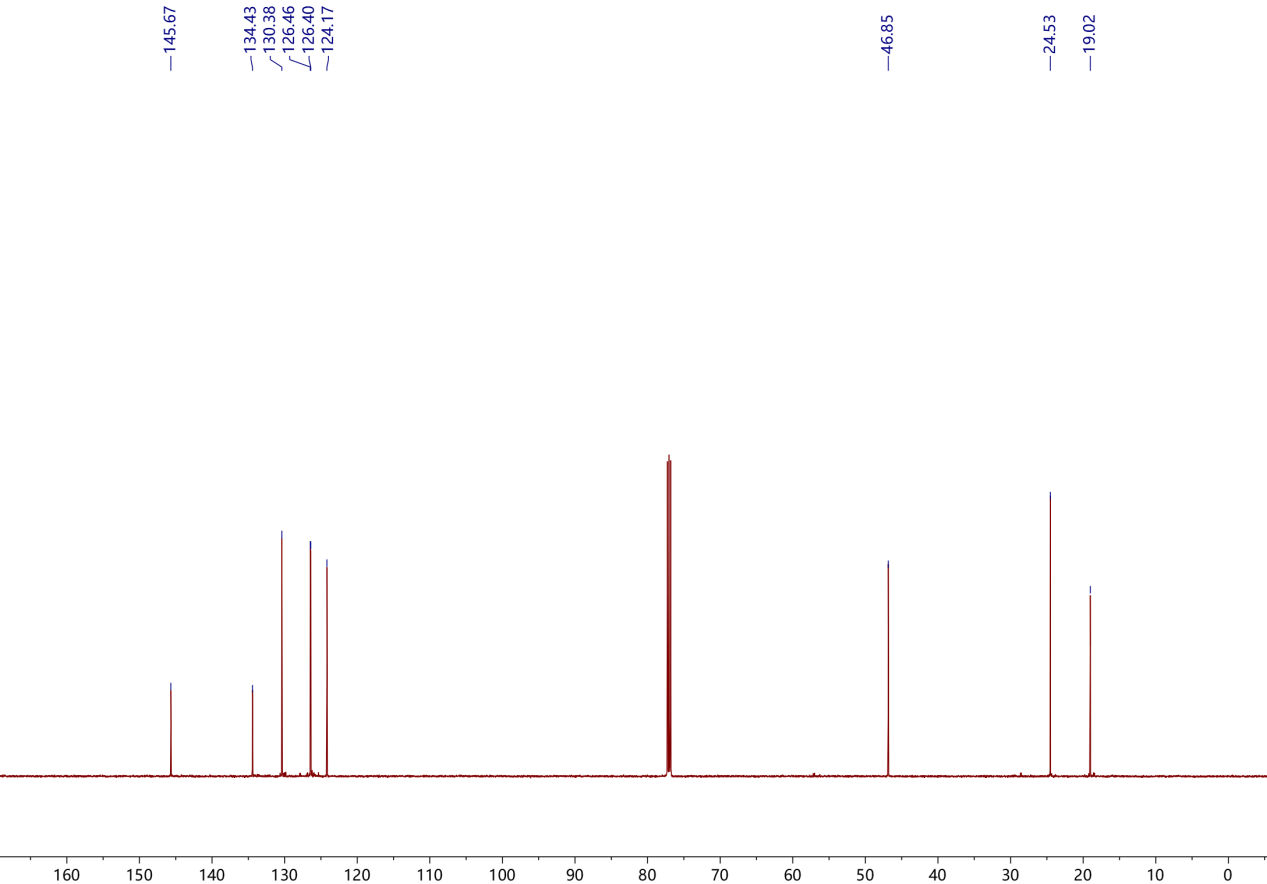

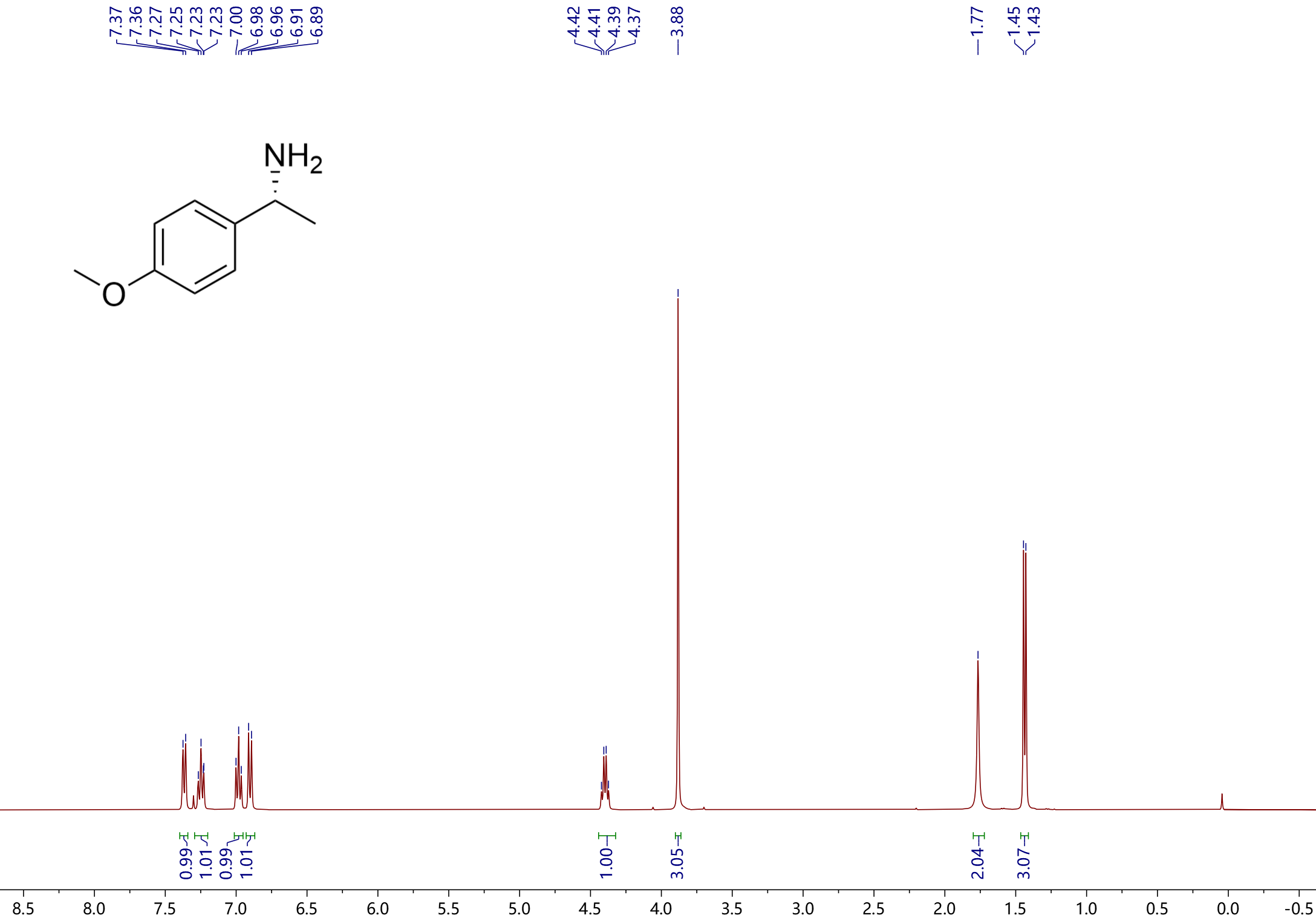

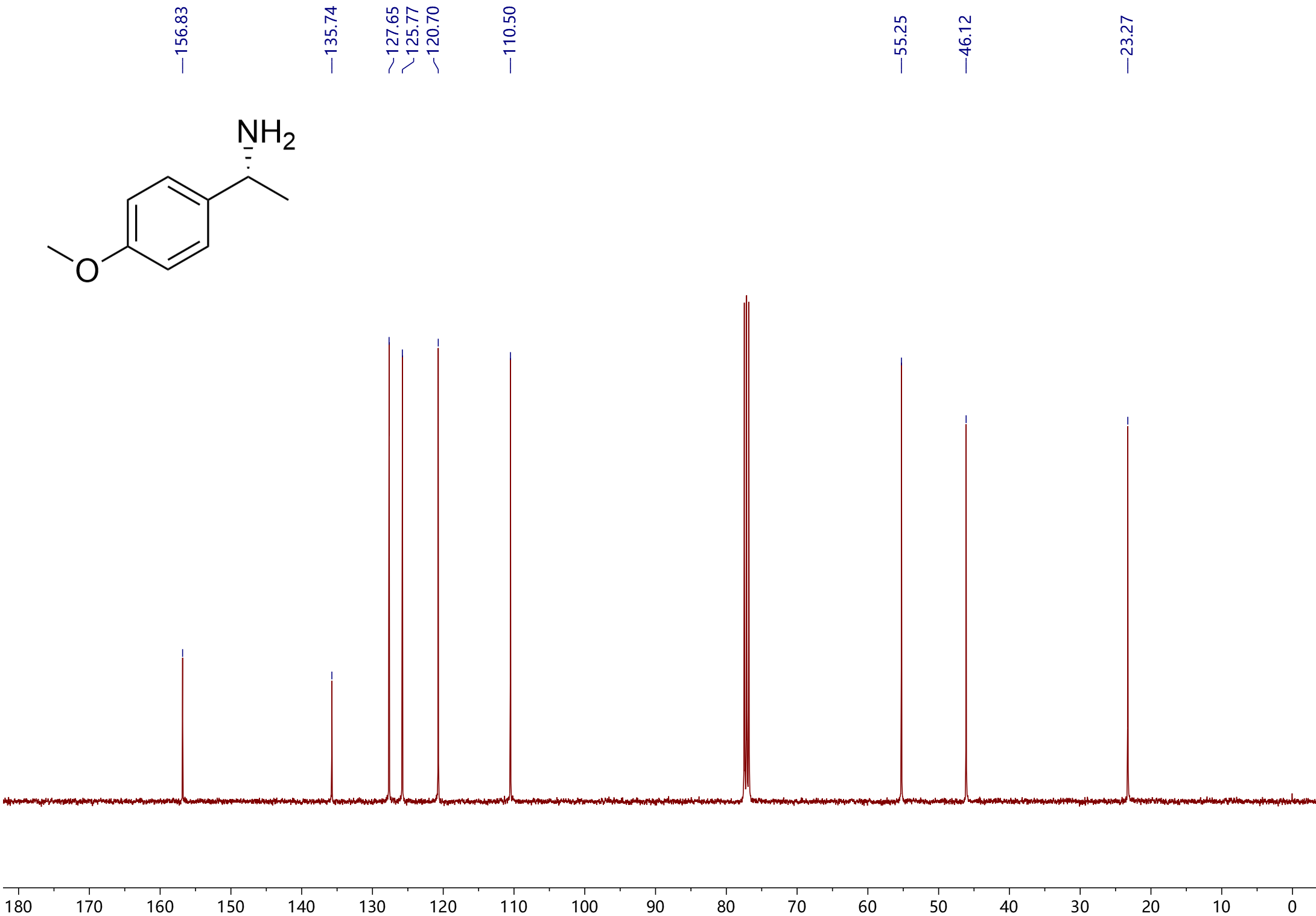

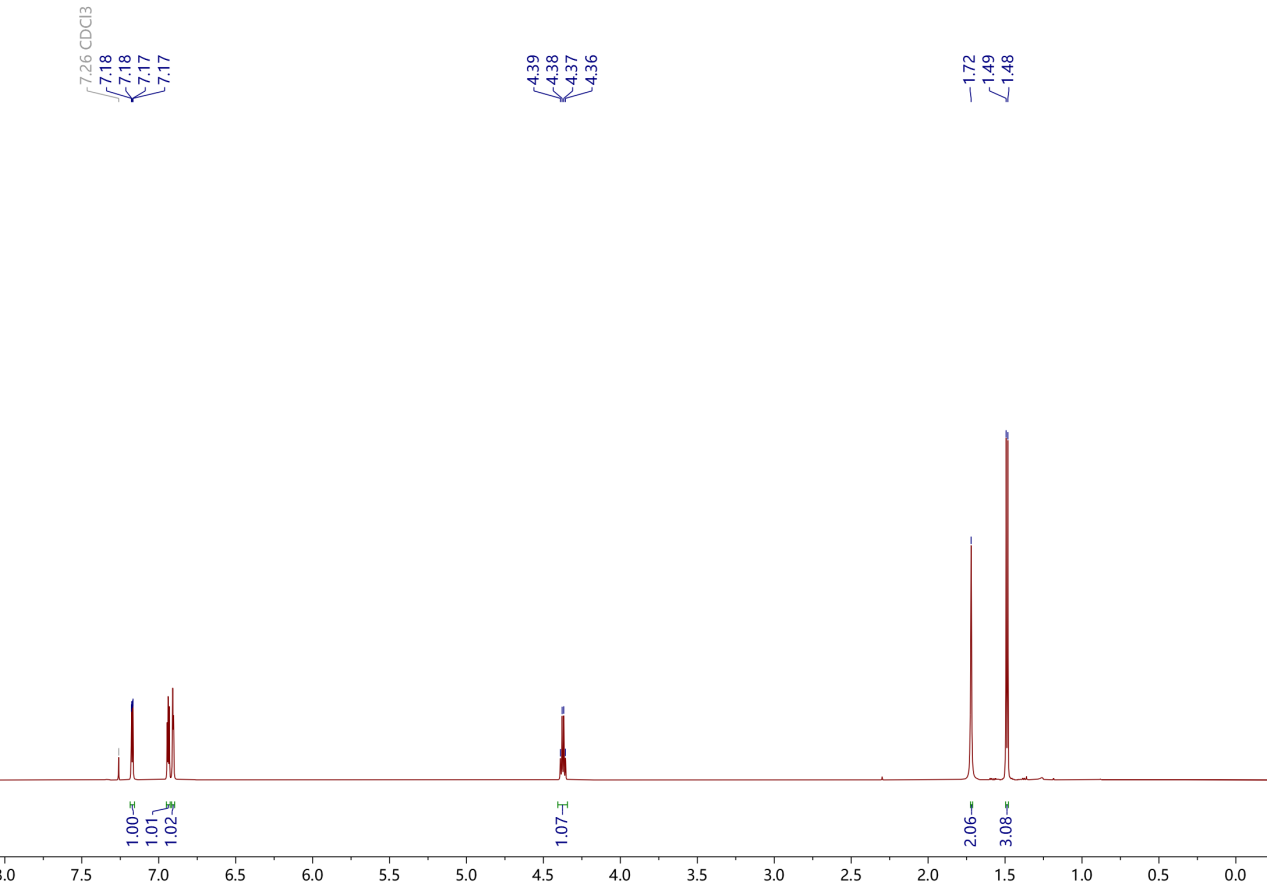

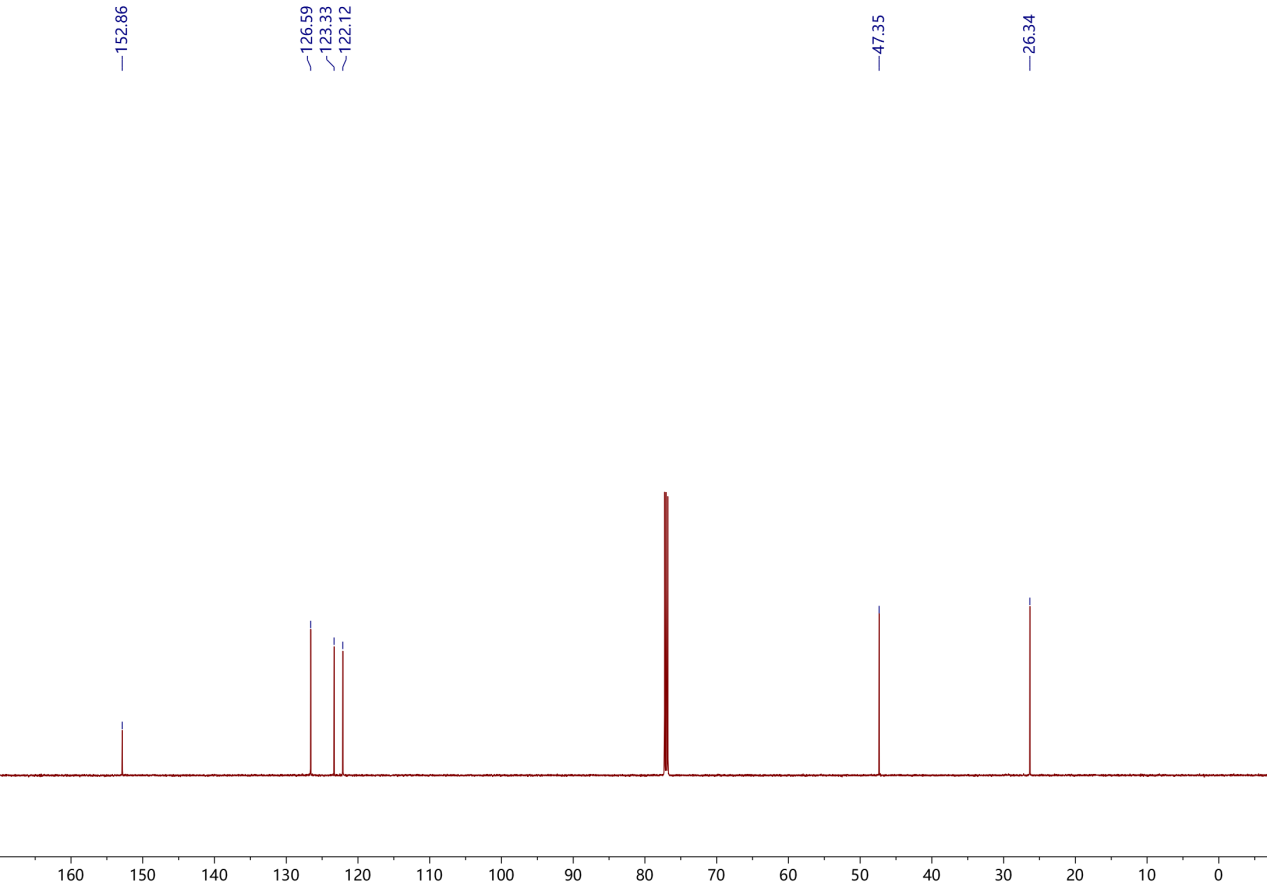

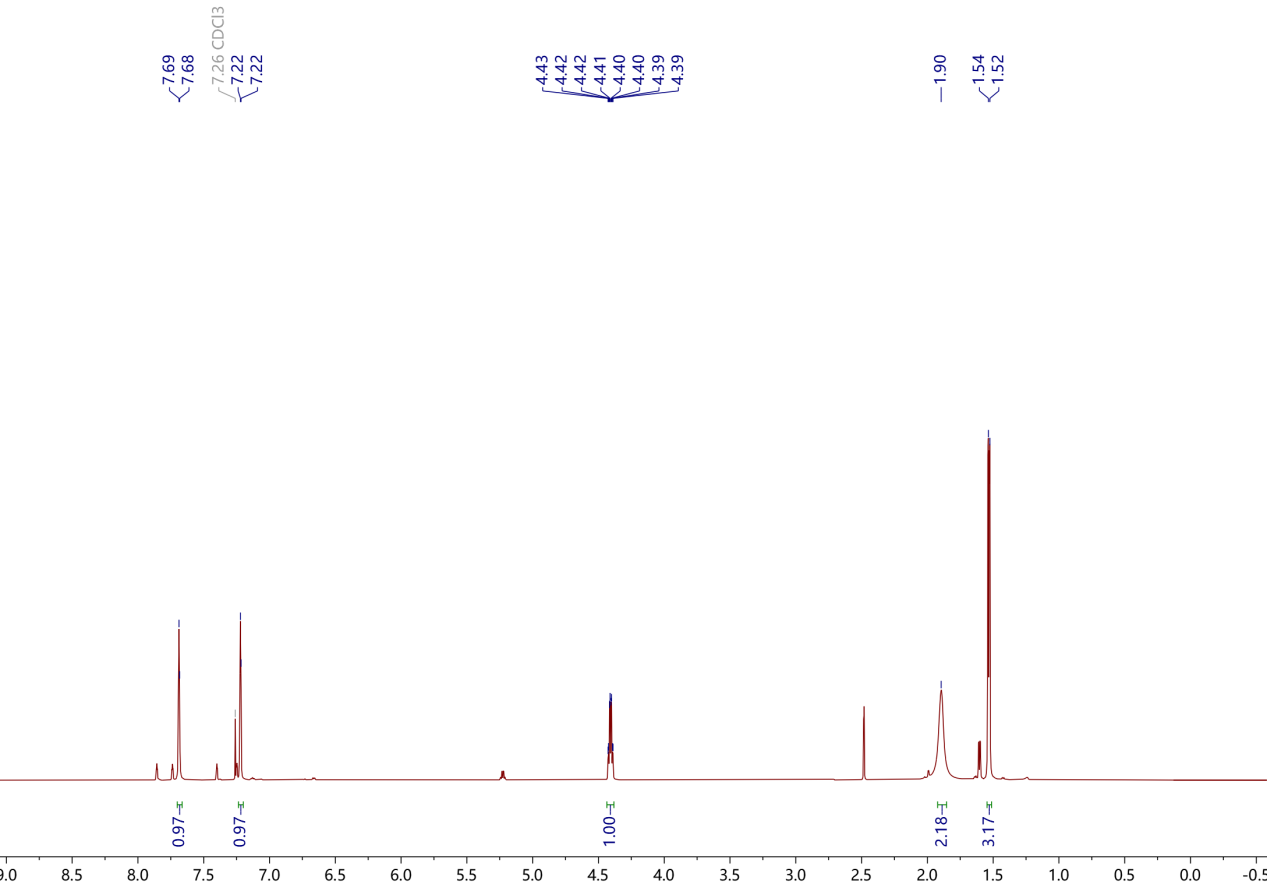

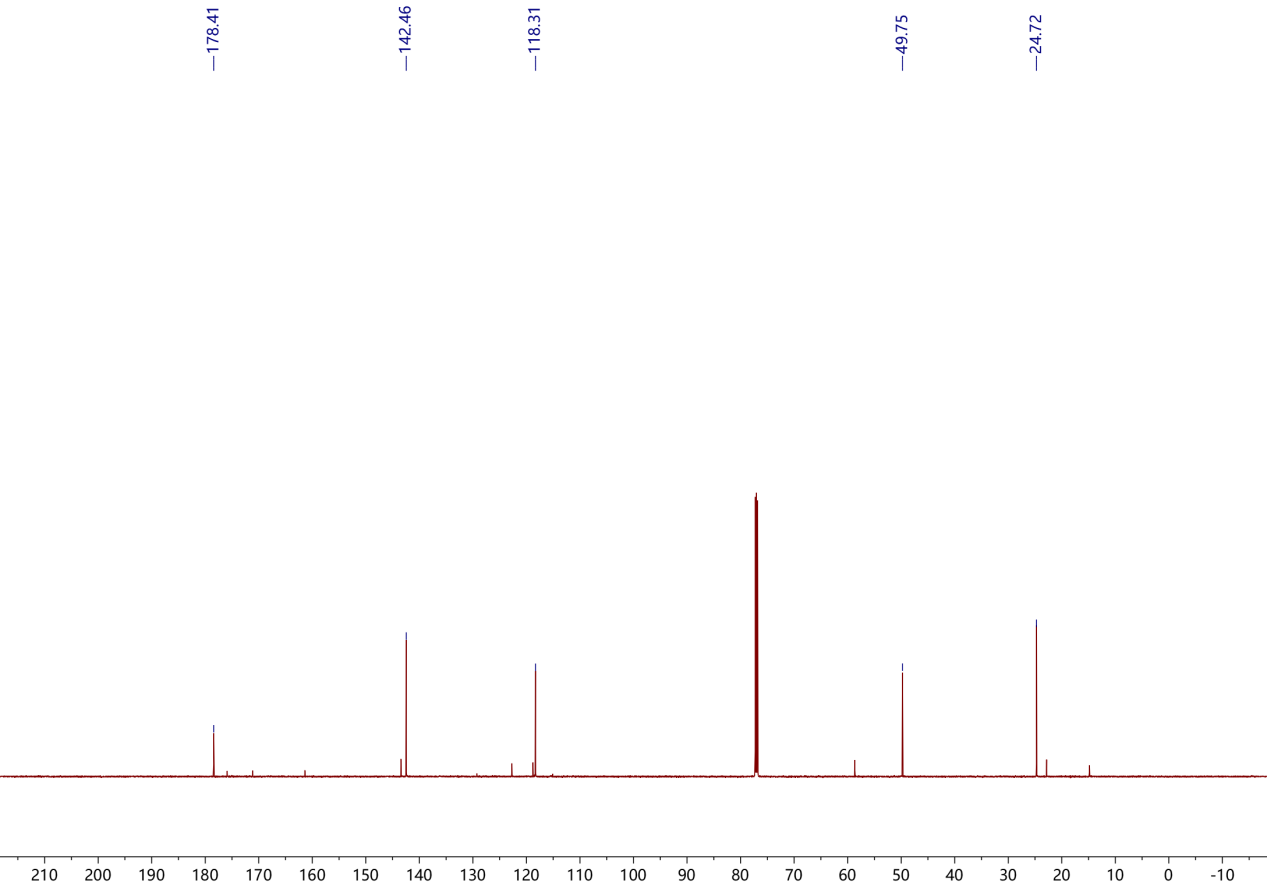

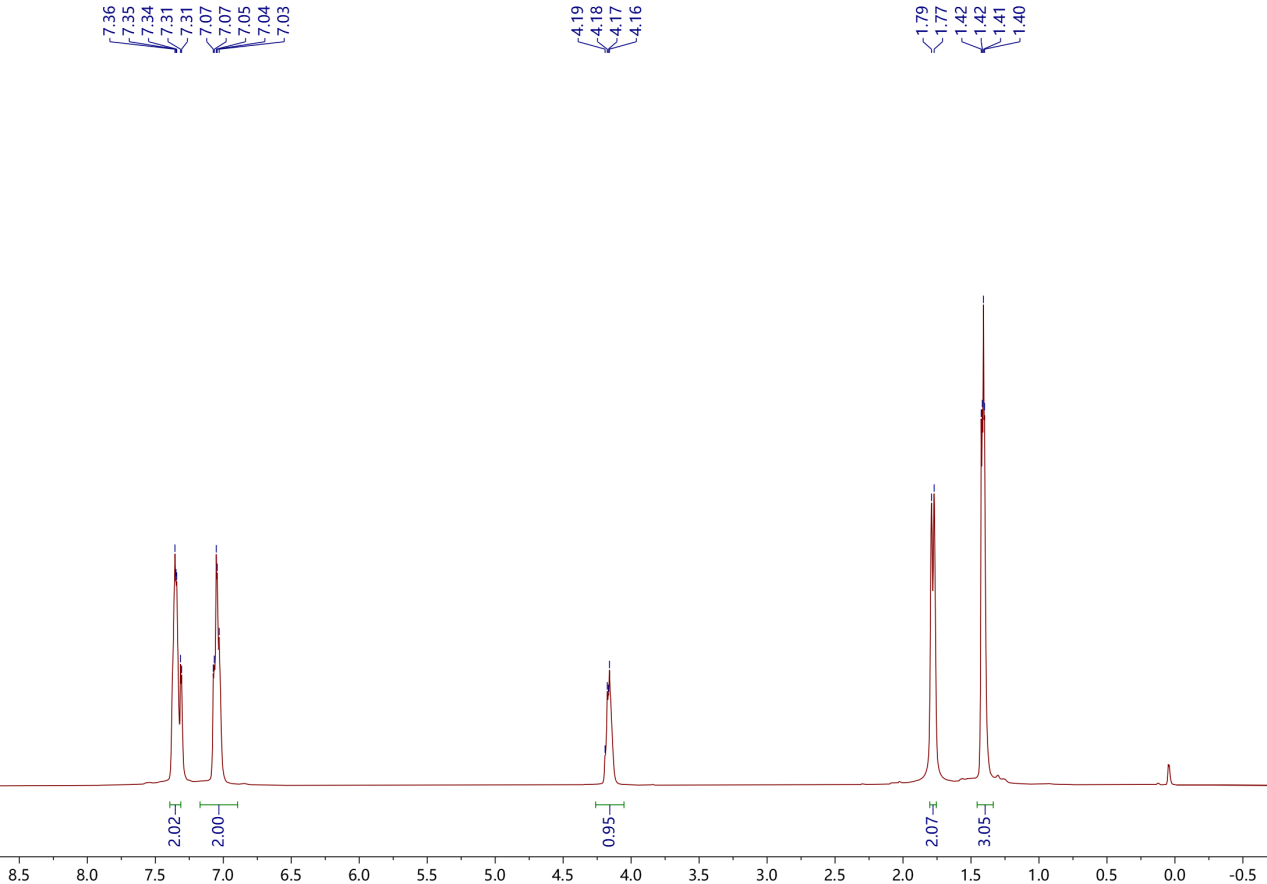

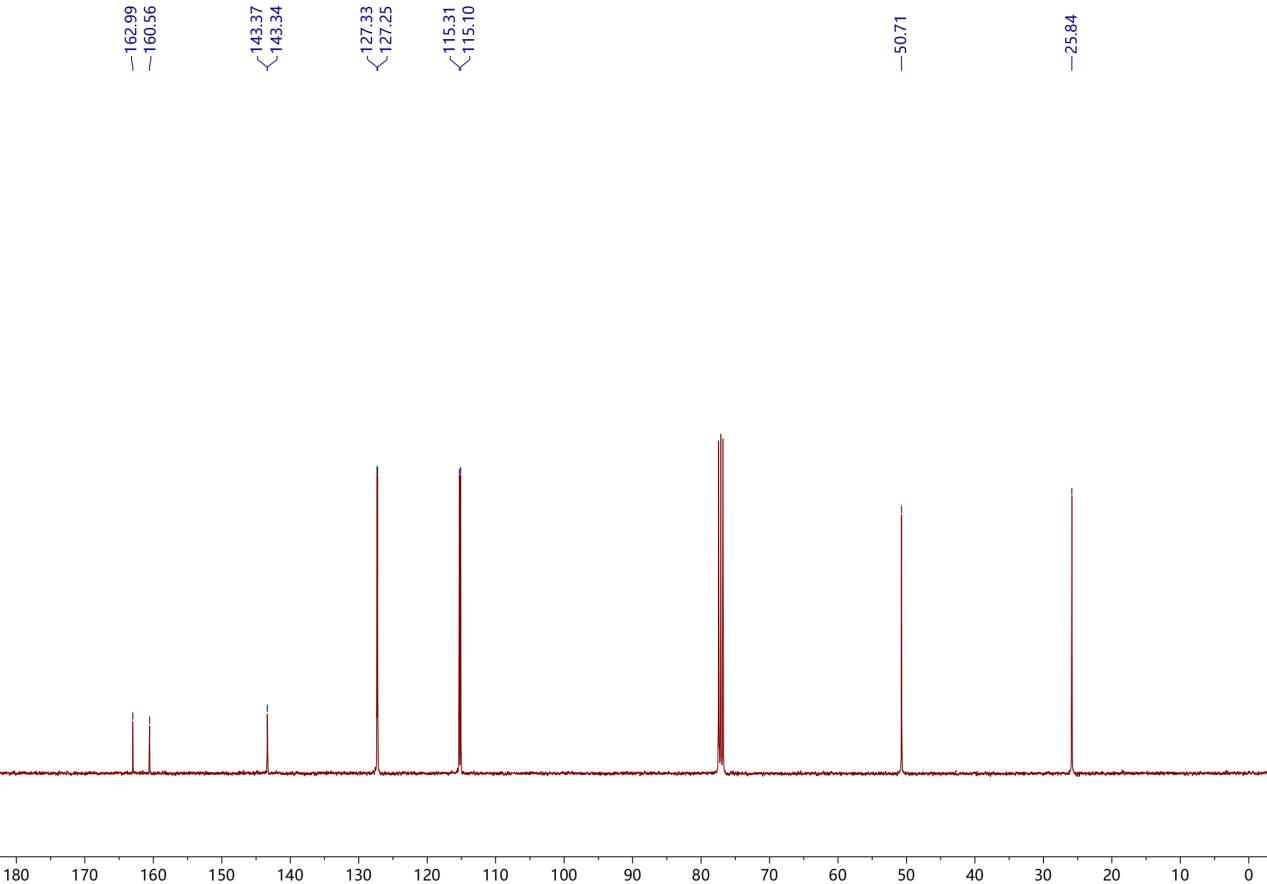

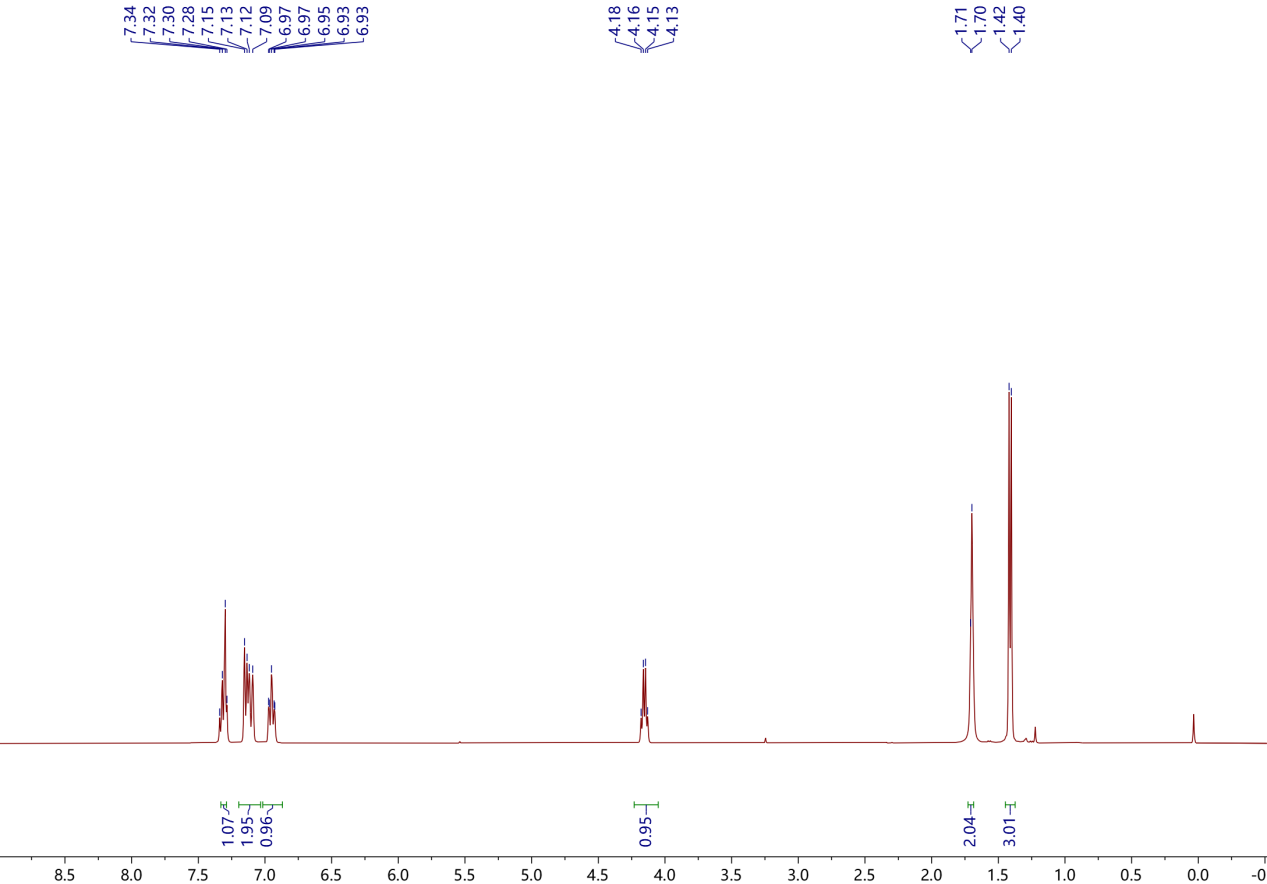

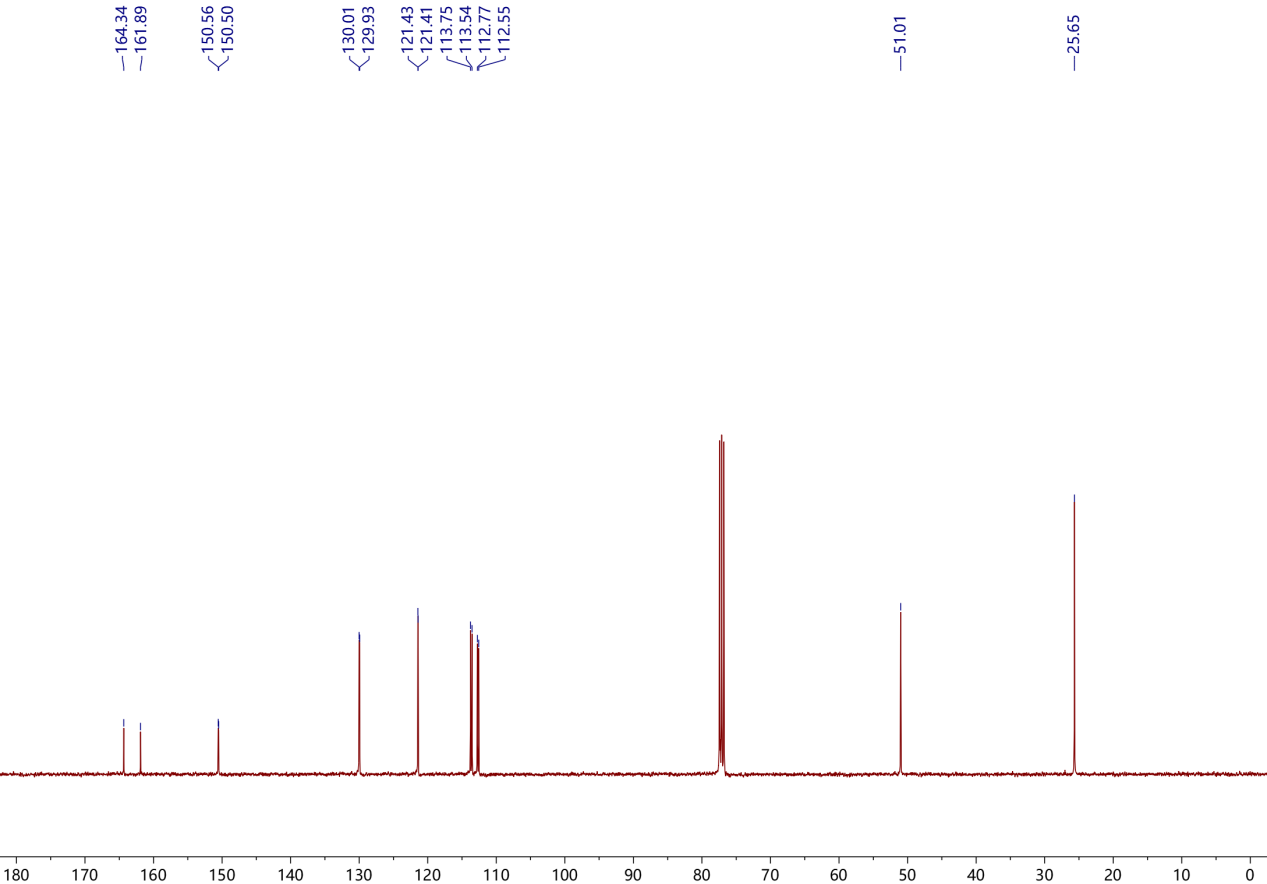

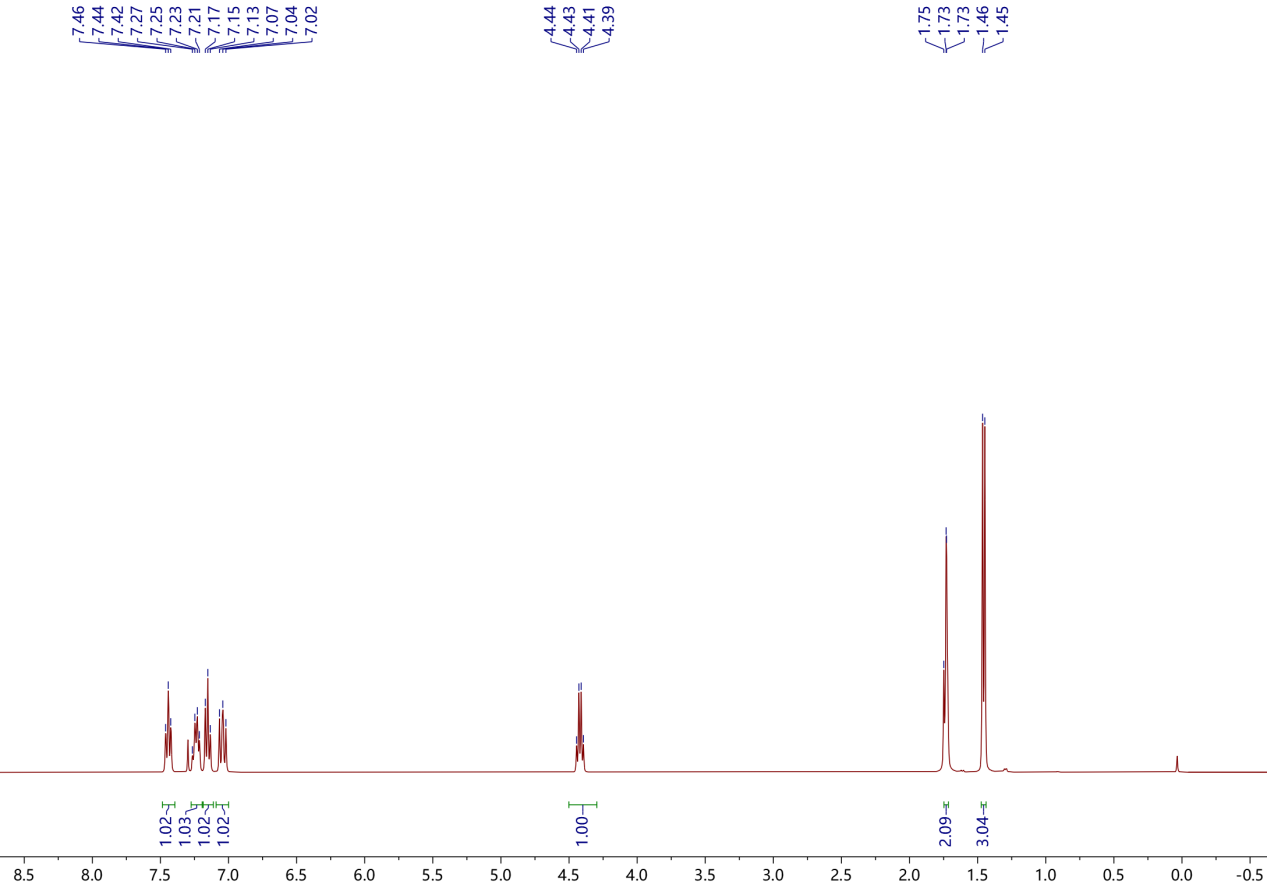

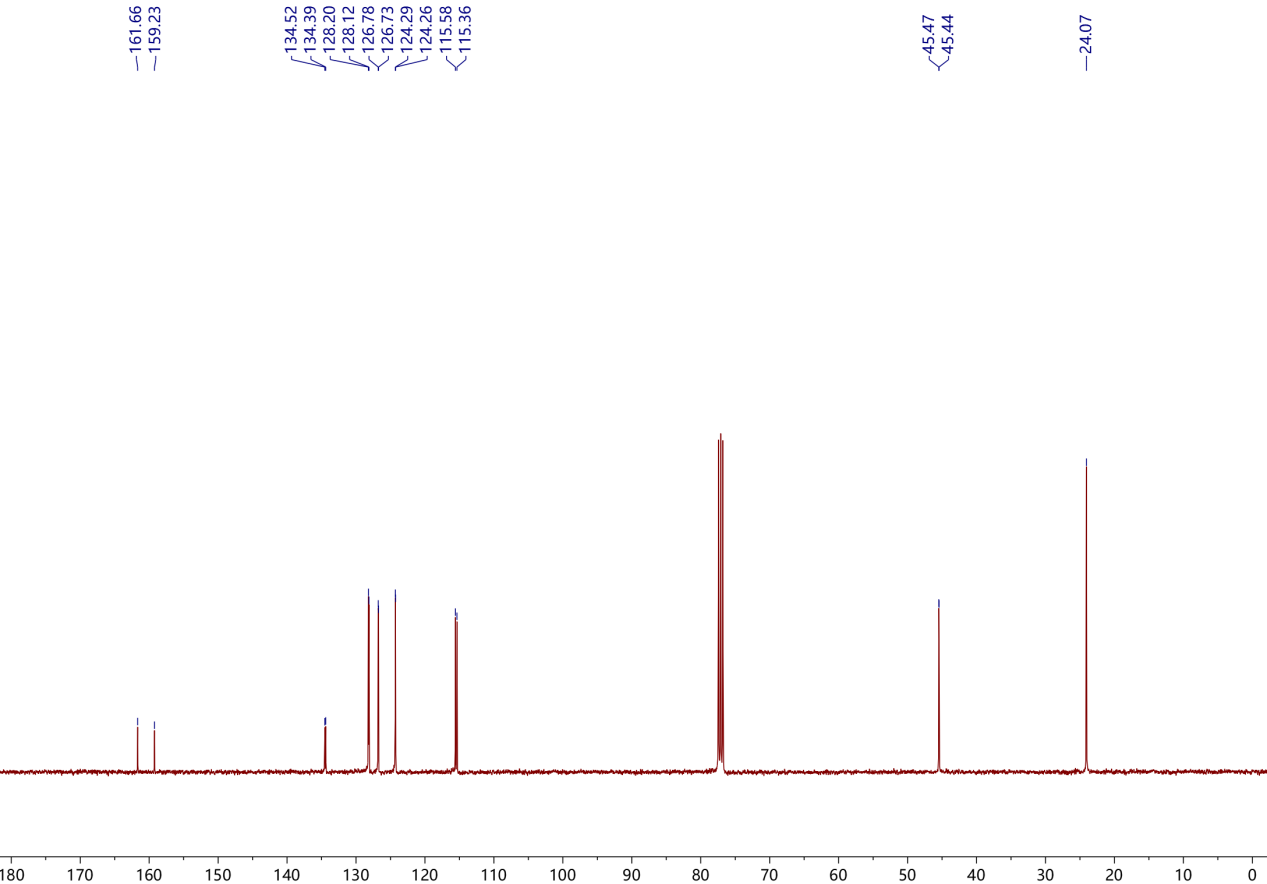

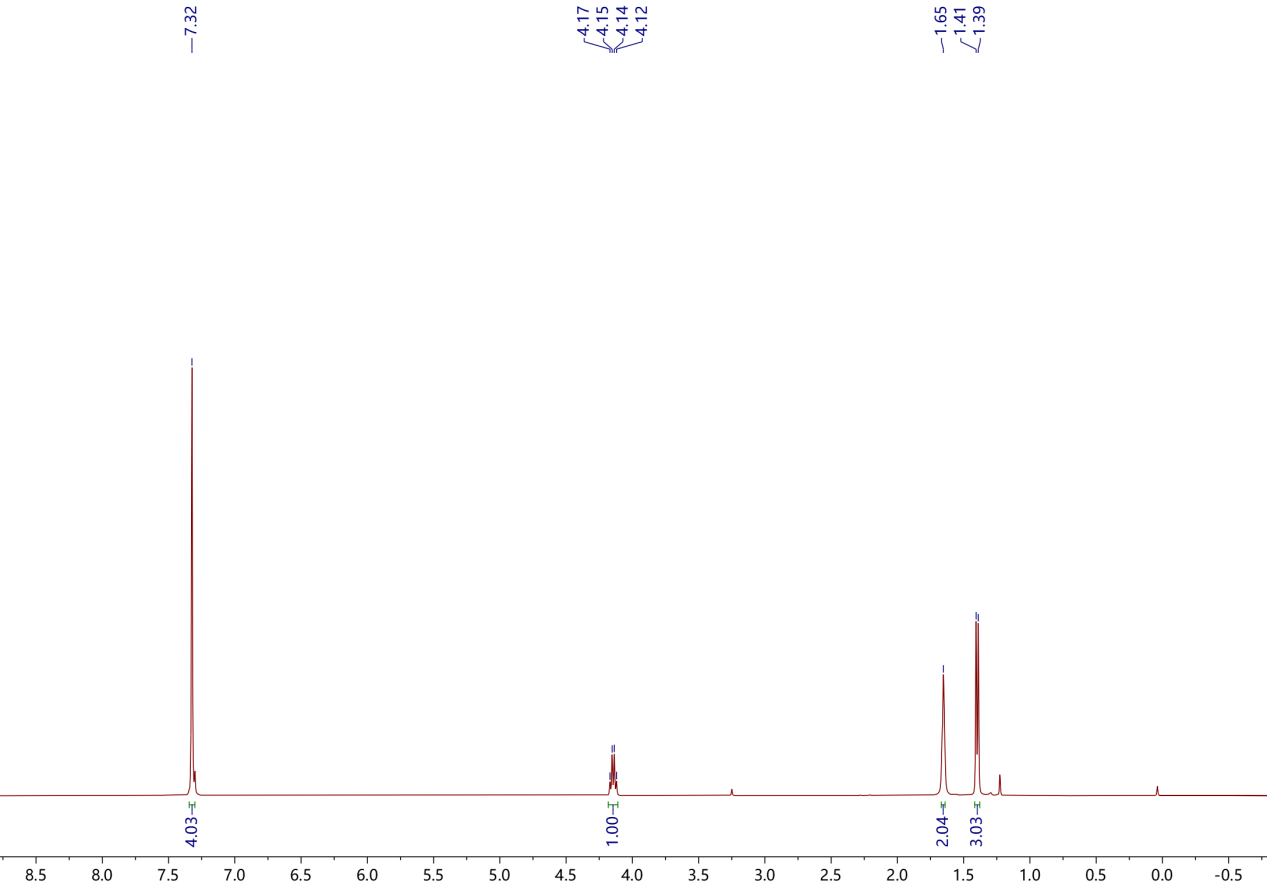

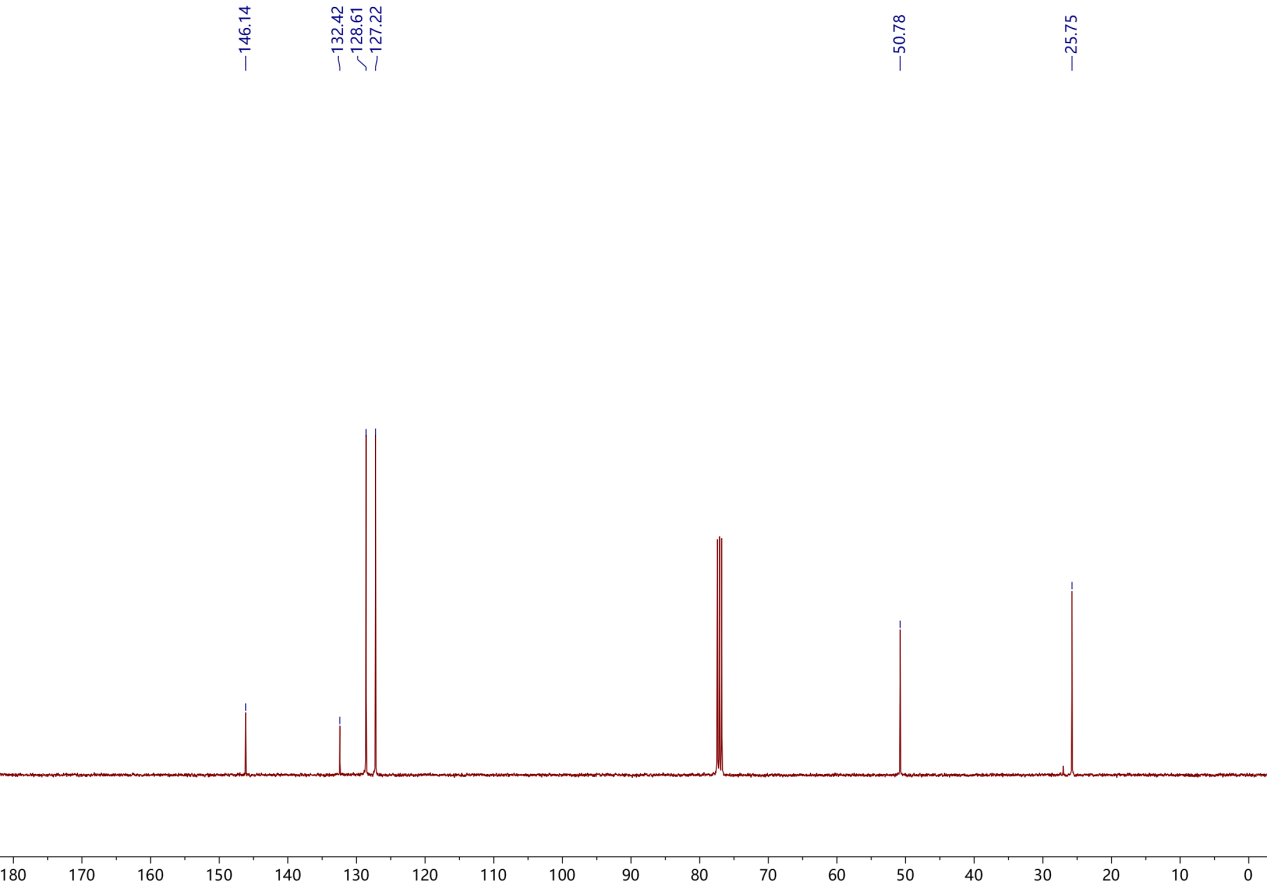

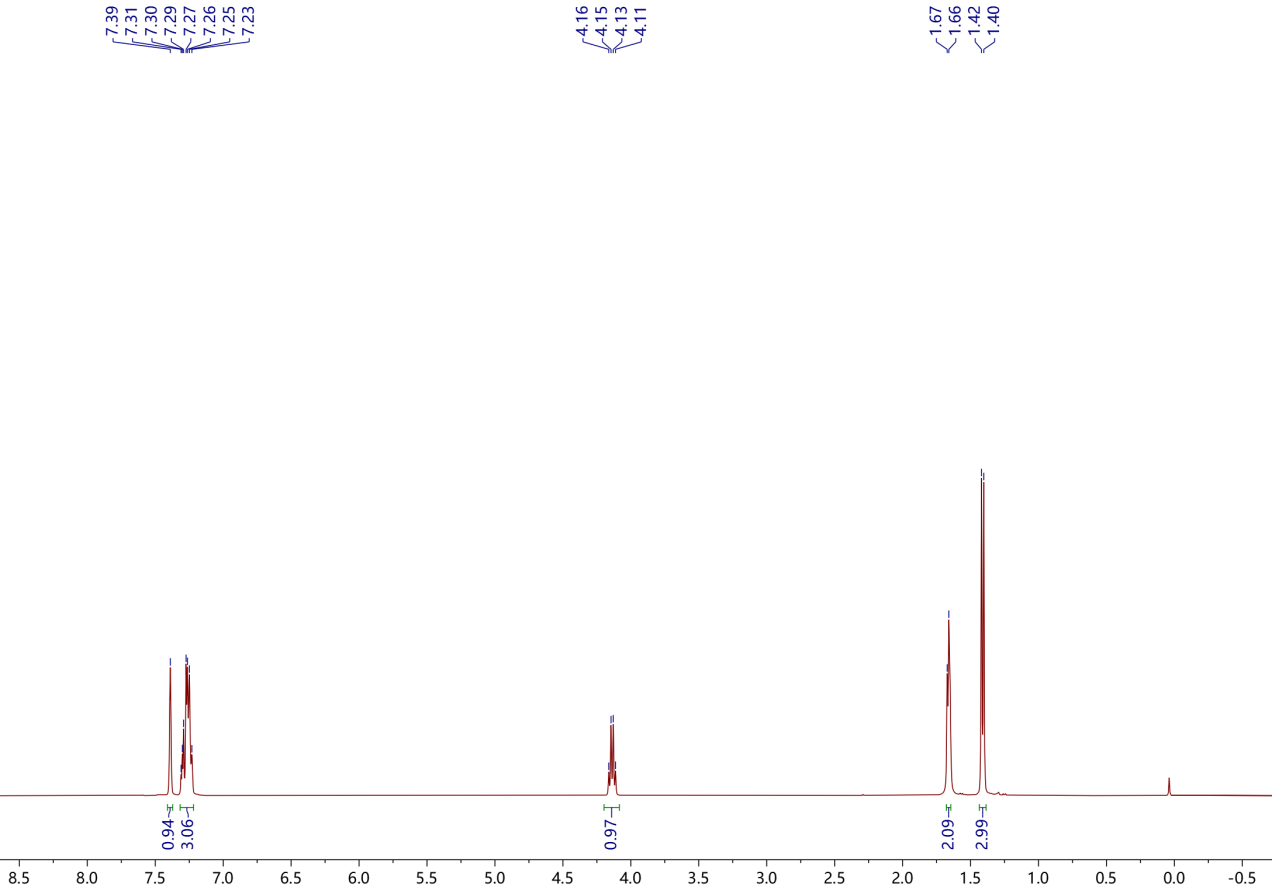

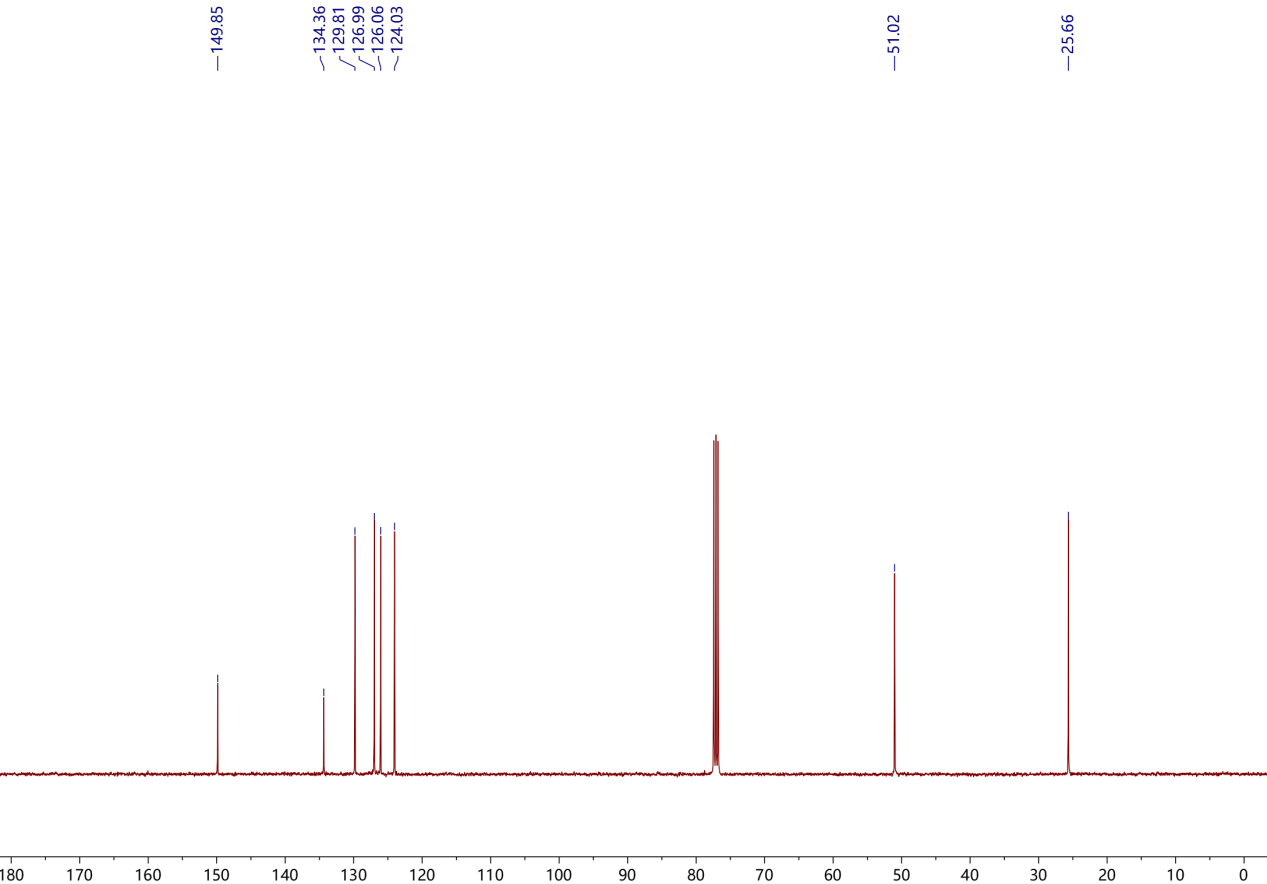

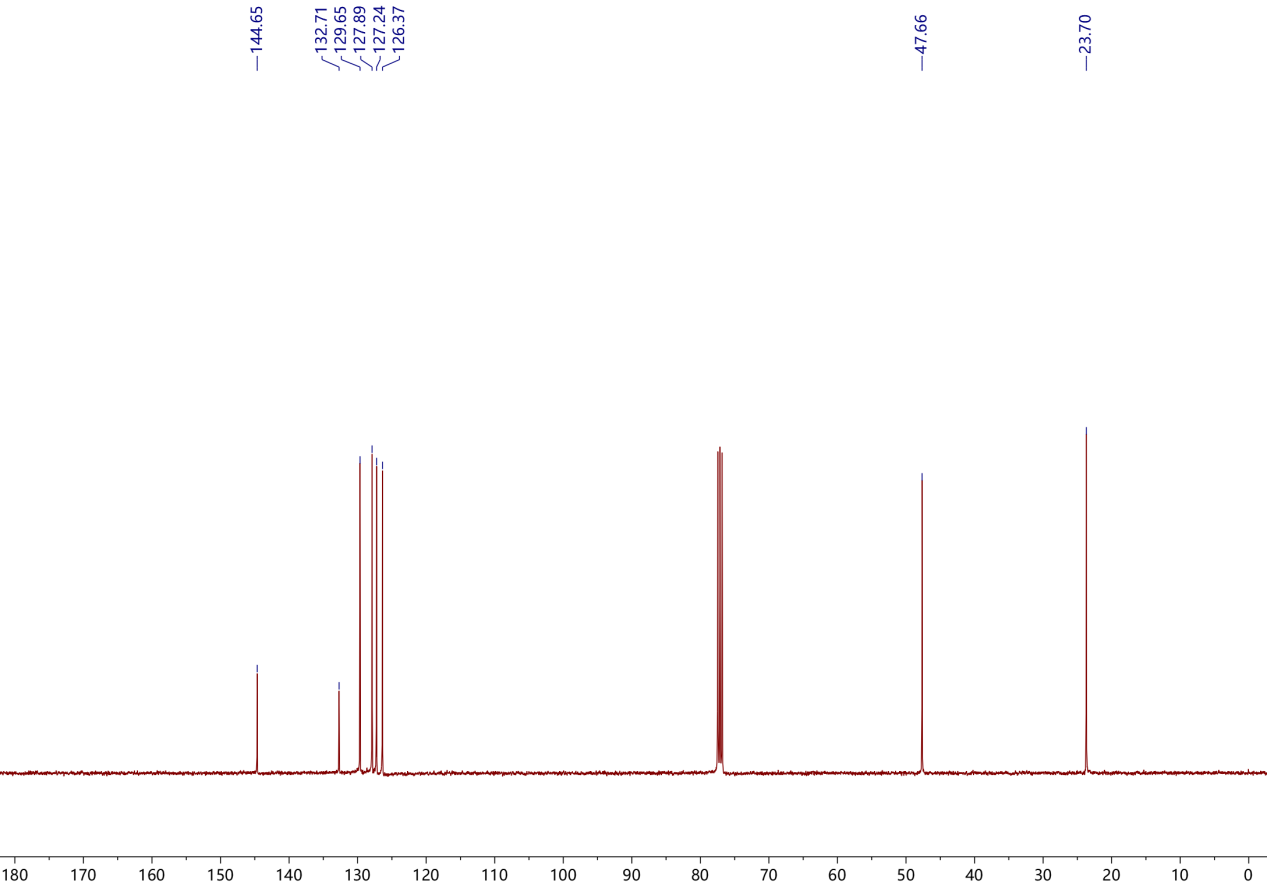

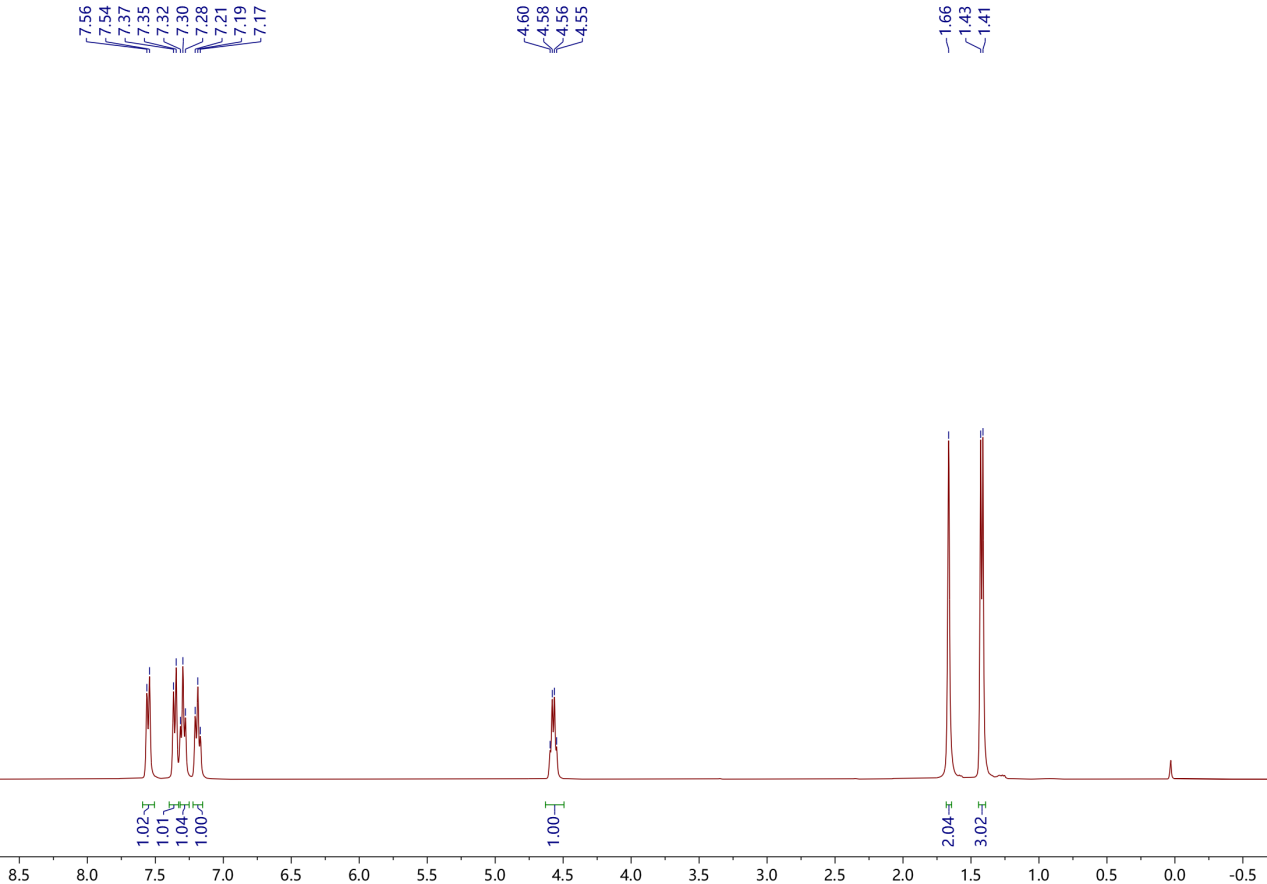

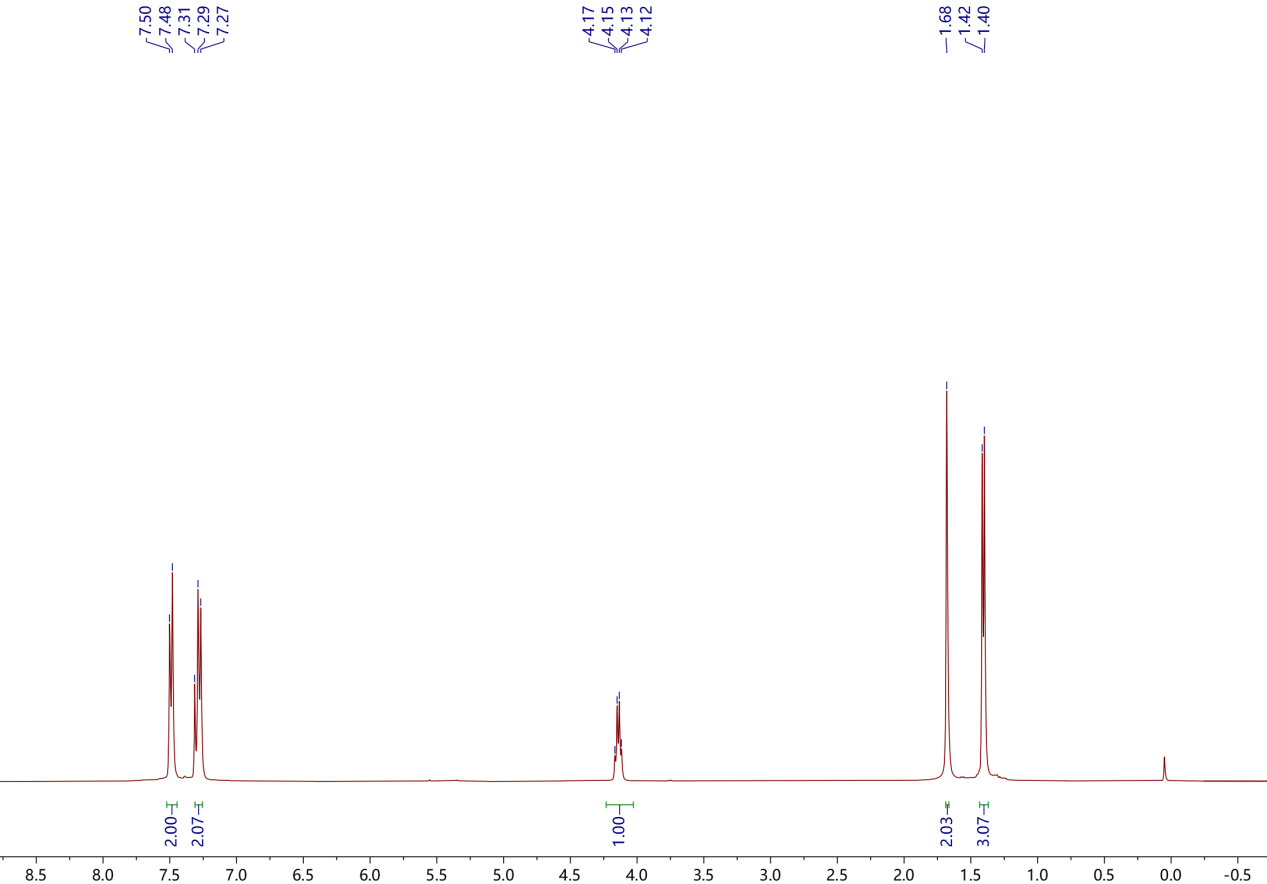

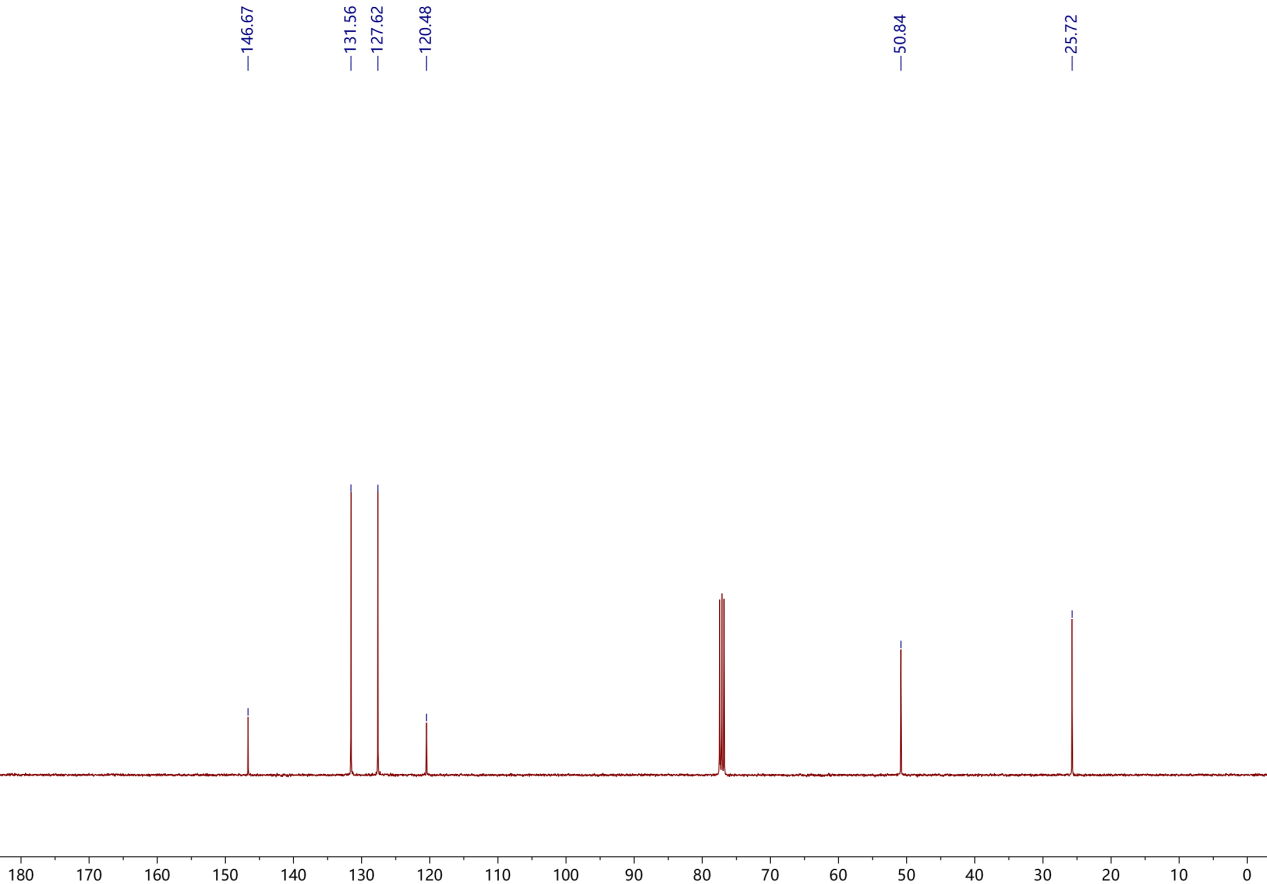

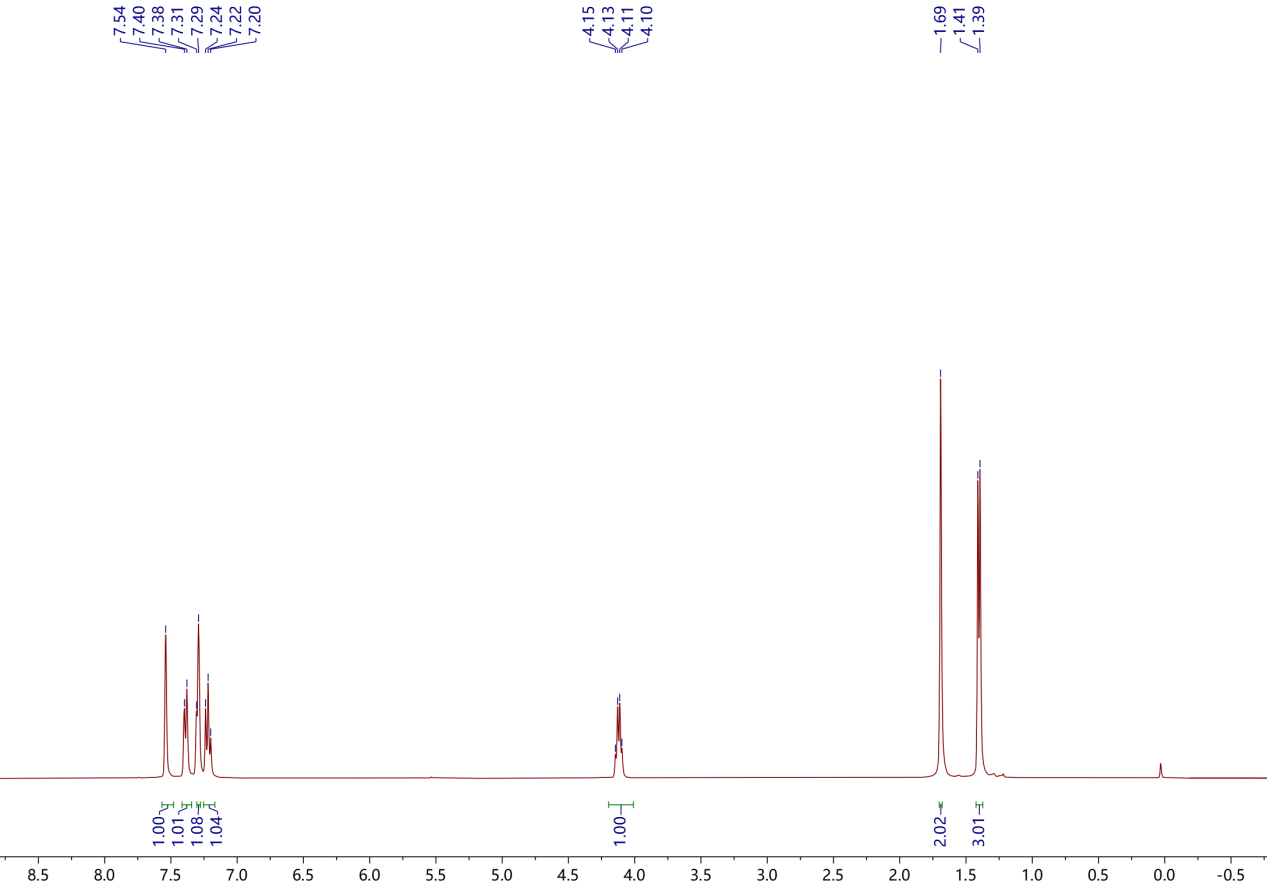

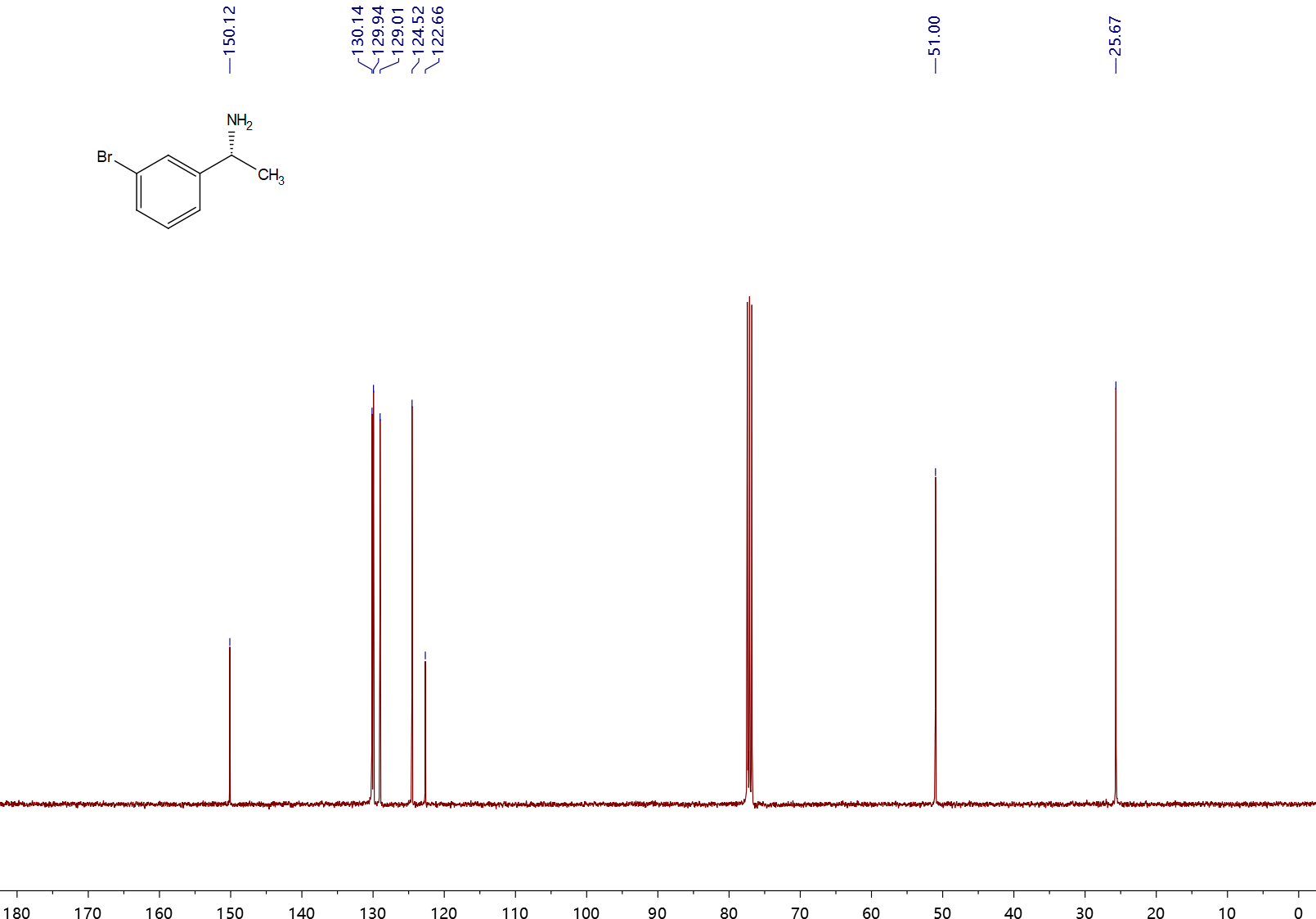

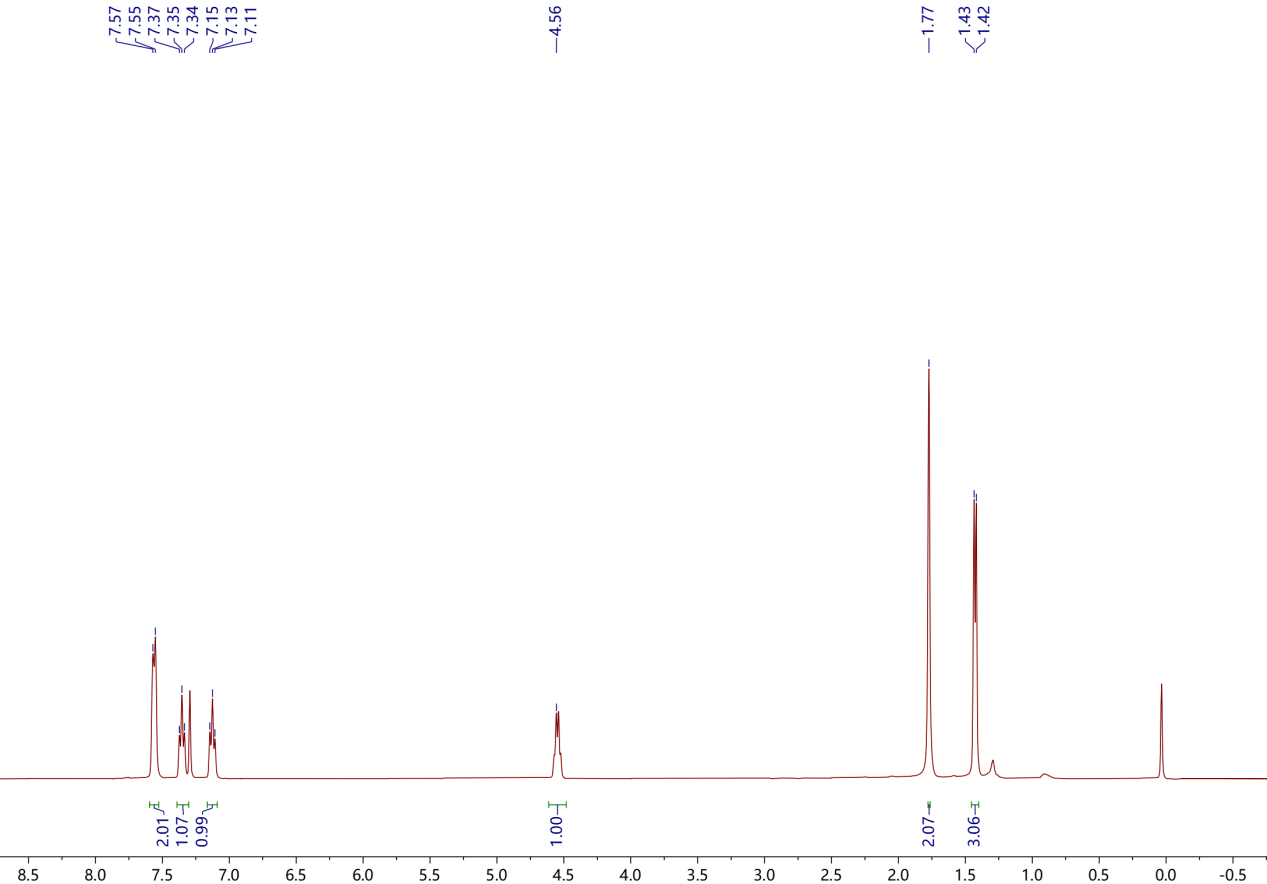

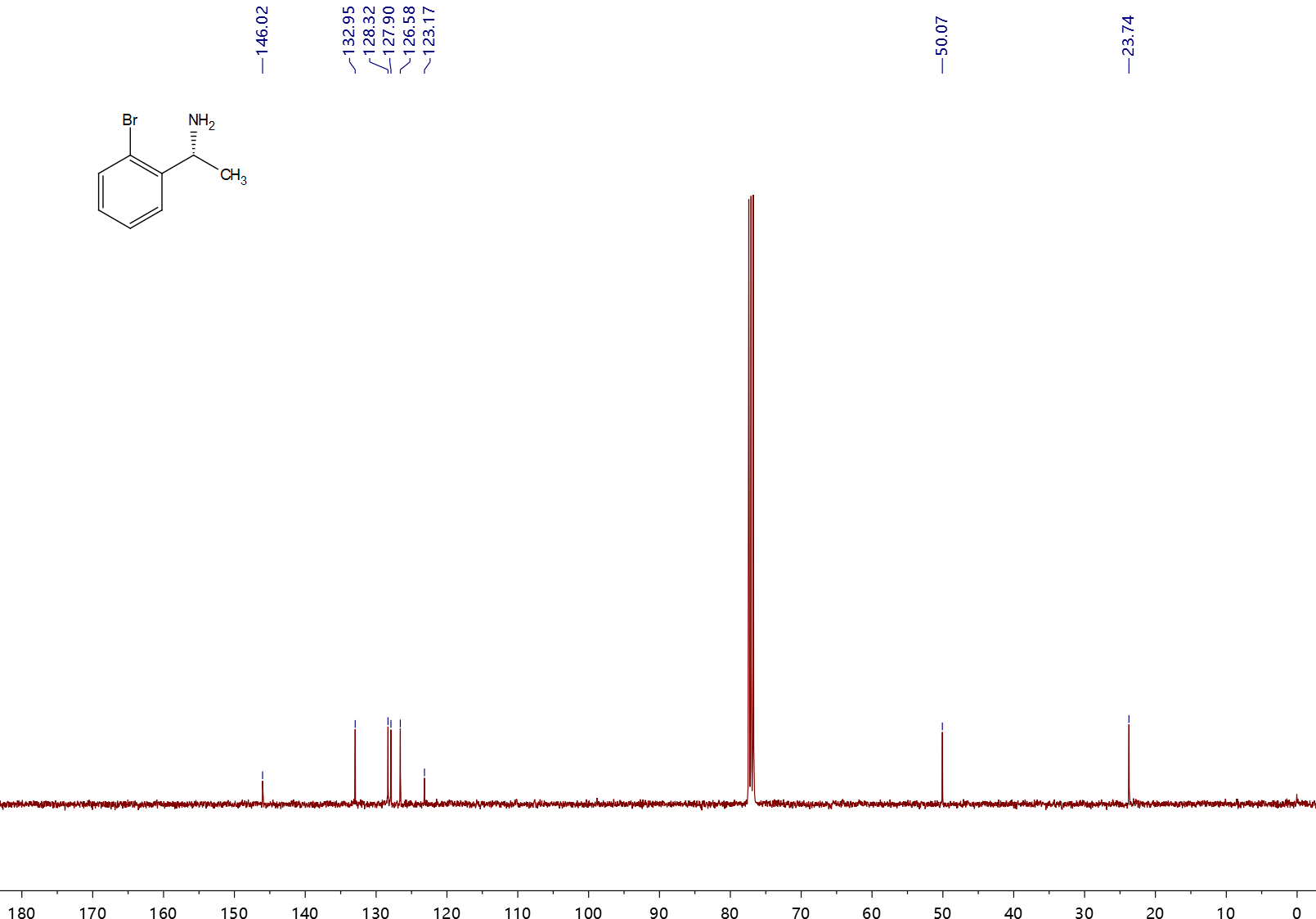


# GC traces


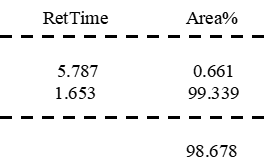

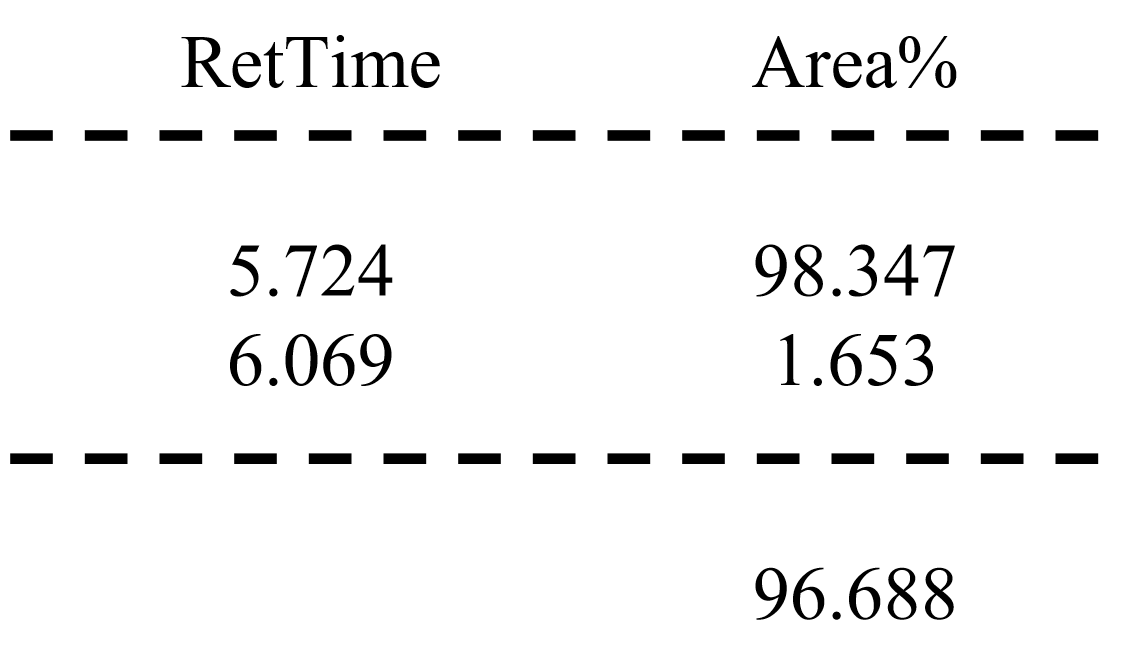

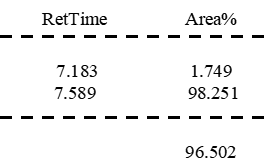

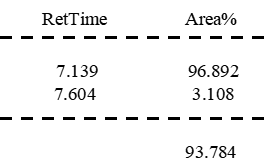

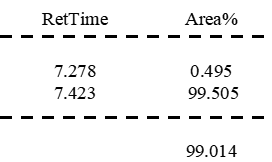

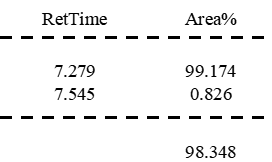

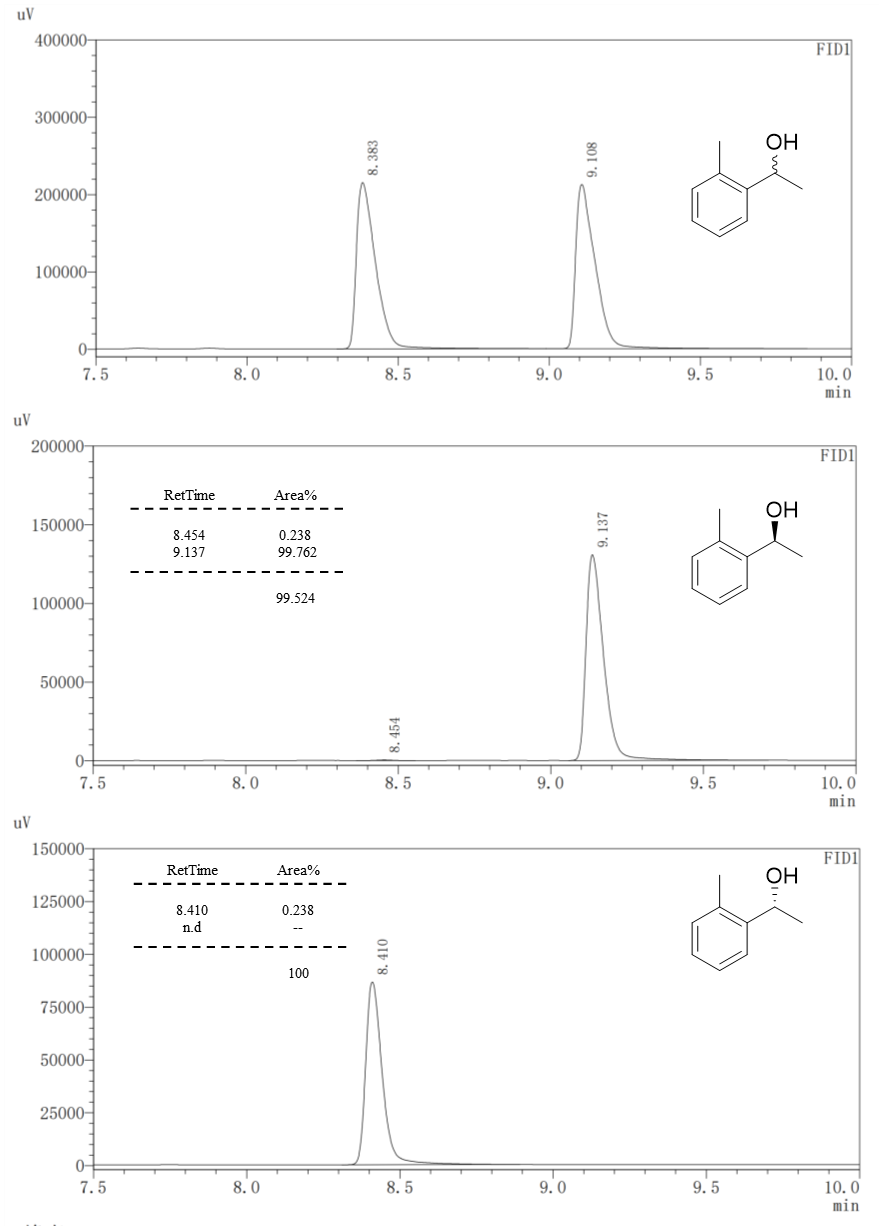

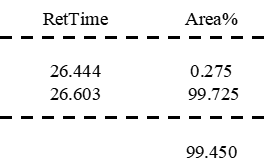

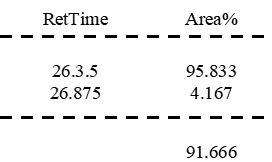

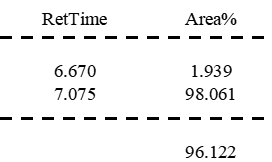

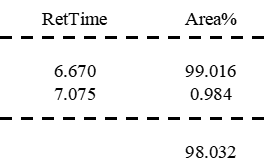

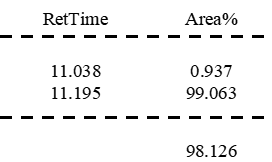

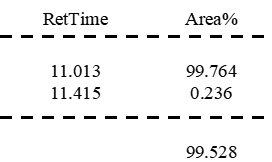

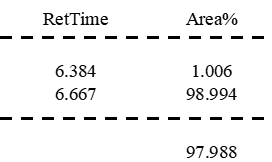

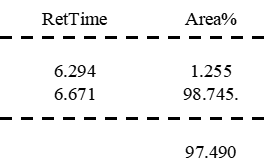

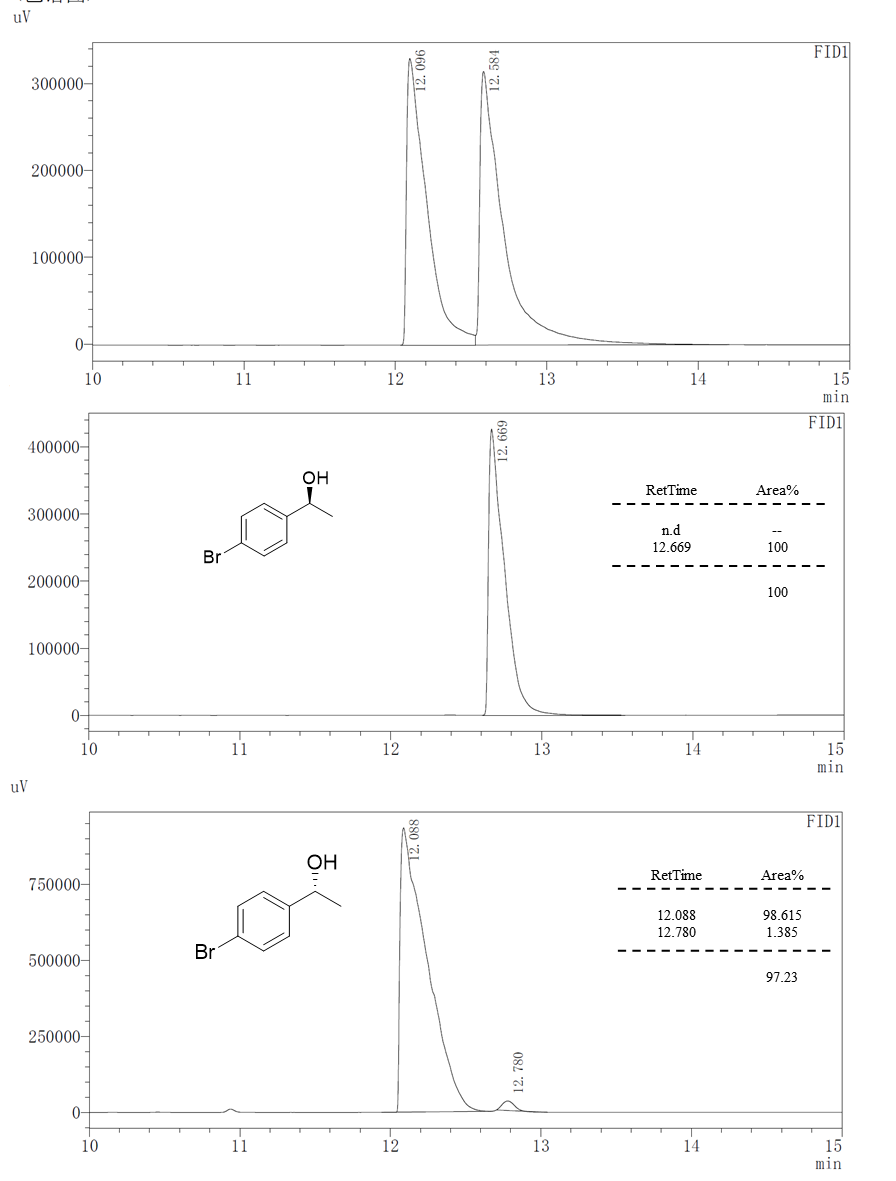

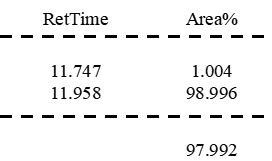

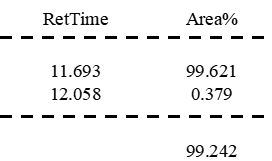

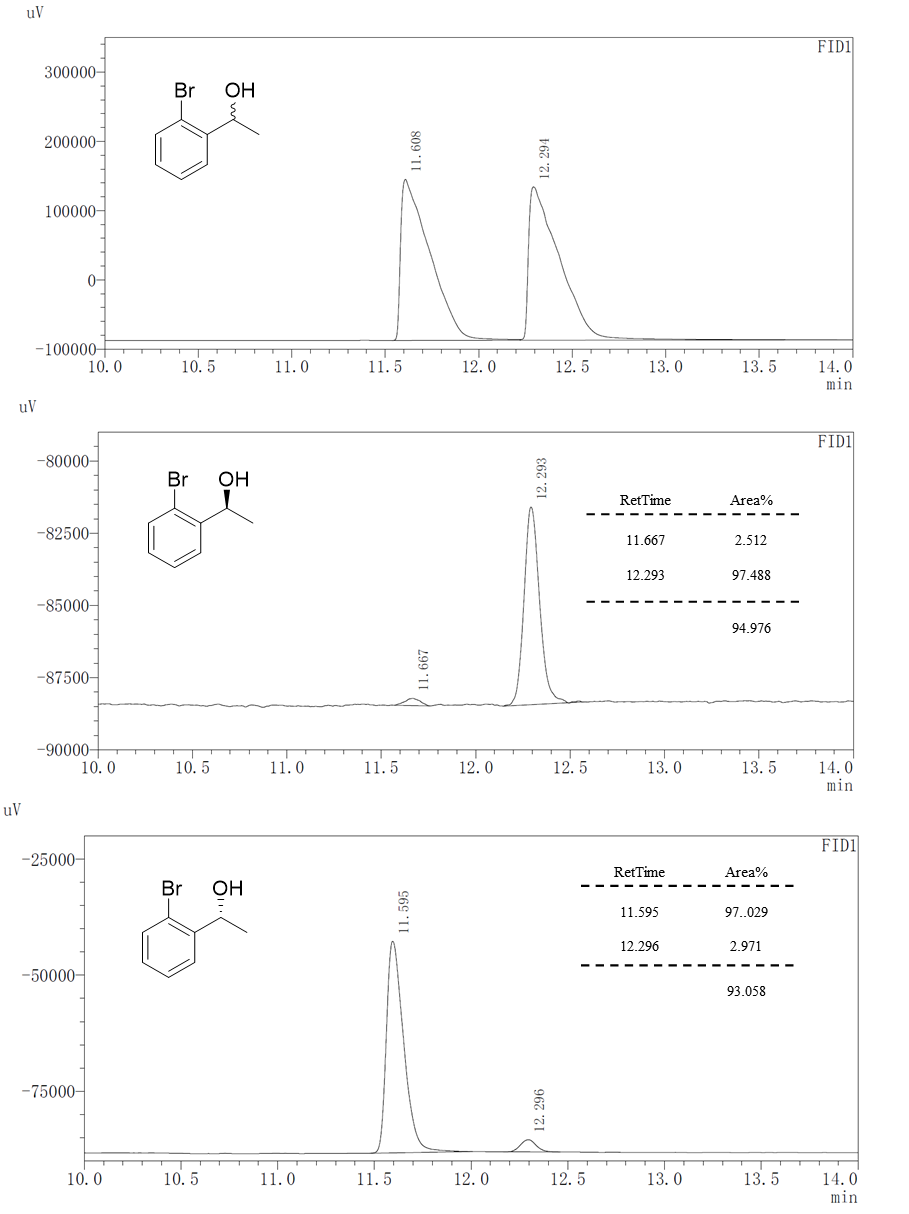

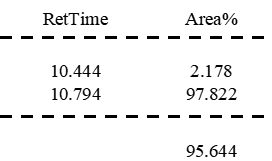

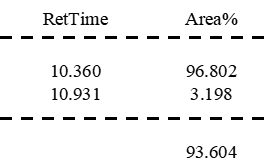

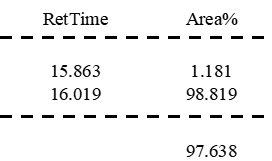

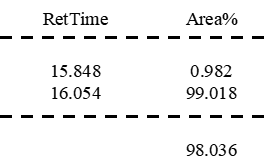

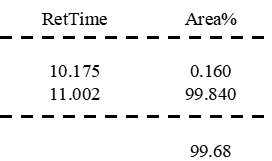

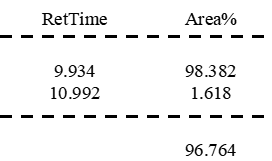

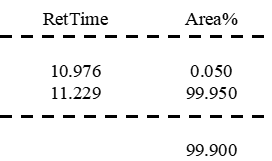

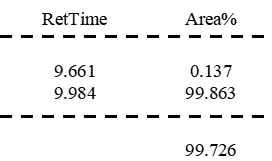

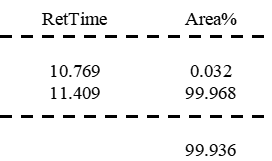

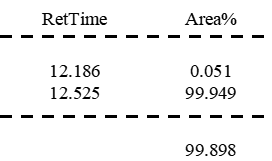

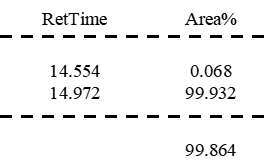

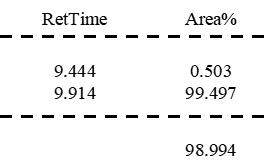

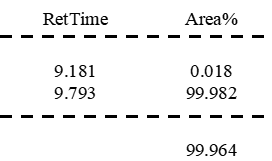

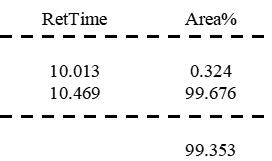

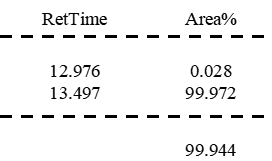

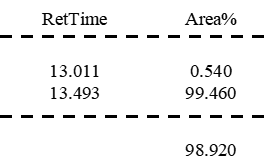

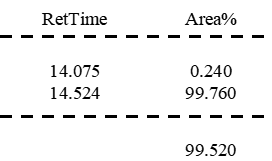


# Reference

[1] W. Kong, Y. Liu, C. Huang, L. Zhou, J. Gao, N. J. Turner, Y. Jiang, *Angew. Chem. Int. Ed.* **2022**, *61*, e202202264.

[2] J. Li, S. Zhao, S. Z. Yang, S. Wang, H. Sun, S. P. Jiang, B. Johannessen, S. Liu, *J. Mater. Chem. A*. **2021**, *9*, 3029.

[3] J. Gao, W. Kong, L. Zhou, Y. He, L. Ma, Y. Wang, L. Yin, Y. Jiang, *Chem. Eng. J.* **2017**, *309*, 70-79.

[4] Y. Matt, I. Wessely, L. Gramespacher, M. Tsotsalas, S. Bräse, *Eur. J. Org. Chem.* **2021**, 239-245.

[5] A. Cuetos, M. García‐Ramos, E. M. Fischereder, A. Díaz‐Rodríguez, G. Grogan, V. Gotor, W. Kroutil, I. Lavandera, *Angew. Chem. Int. Ed.* **2016**, *55*.

[6] B. Ravel, M. Newville, *J. Synchrotron Rad.* **2005**, *12*, 537-541.

[7] E. A. Hudson, J. J. Rehr, J. J. Bucher, *Phys. Rev. B* **1995**, *52*, 13815-13826.

[8] G. Kresse, D. Joubert, *Physical Review B* **1999**, *59*, 1758-1775.

[9] J. P. Perdew, K. Burke, M. Ernzerhof, *Phys. Rev. Lett.* **1996**, *77*, 3865.

[10] P. E. Blochl, *Phys Rev B Condens Matter* **1994**, *50*, 17953-17979.

[11] S. Grimme, J. Antony, S. Ehrlich, H. Krieg, *J. Chem. Phys.* **2010**, *132*, 154104.

[12] B. Jia, L. Bai, Z. Han, R. Li, J. Huangfu, C. Li, J. Zheng, Y. Qu, K. Leng, Y. Wang, J. Bai, *ACS Appl. Mater. Interfaces* **2022**, *14*, 10337-10343.

[13] E. Yuan, M. Zhou, G. Shi, P. Jian, X. Hou, *Nano Res.* **2022**, *15*, 8791–8803.

[14] Y. Xiong, W. Sun, Y. Han, P. Xin, X. Zheng, W. Yan, J. Dong, J. Zhang, D. Wang, Y. Li, *Nano Res.* **2021**, *14, 2418–2423*.

[15] S. Shen, J. Chen, Y. Wang, C. L. Dong, F. Meng, Q. Zhang, Y. Huangfu, Z. Lin, Y. C. Huang, Y. Li, *Sci. Bull.* **2022**, *67*, 520-528.

[16] X. Liang, D. Wang, Z. Zhao, T. Li, Z. Chen, Y. Gao, C. Hu, *Appl. Catal. B* **2021**, *303*, 120877.

[17] C. Chu, J. Yang, X. Zhou, D. Huang, J. H. Kim, *Environ. Sci. Technol.* **2020**, *55*.

[18] J. Shi, Y. Wei, D. Zhou, *ACS Catal.* **2022**, 12.

[19] M. Qian, X. L. Wu, M. Lu, L. Huang, W. Li, H. Lin, J. Chen, S. Wang, X. Duan, *Adv. Funct. Mater.* **2023**, *33*, 2208688.

[20] F. Yu, T.-T. Huo, Q.-H. Deng, G.-A. Wang, Y.-G. Xia, H.-P. Li, W.-G. Hou, *Chemical Science* **2022**, *13*, 754.

[21] P. Zhang, Y. Gong, H. Li, Z. Chen, Y. Wang, *Nat. Commun.* **2013**, *4*, 1593.

[22] X. Liu, Z. Z. Nie, L. Y. Zhang, S. C. Mei, Y. Chen, B. S. Zhang, R. L. Zhu, Z. G. Liu, *Green Chem.* **2017**, *19*, 2164-2173.

[23] R. Xie, G. Fan, L. Yang, F. Li, *Catal. Sci. Technol.* **2015**, *5*, 540-548.

[24] G. Q. Song, Y. X. Lu, Q. Zhang, F. Wang, X. K. Ma, X. F. Huang, Z. H. Zhang, *RSC Adv.* **2014**, *4*, 30221-30224.

[25] K. Nakatsuka, T. Yoshii, Y. Kuwahara, K. Mori, H. Yamashita, *Phys. Chem. Chem. Phys.* **2016**, *19*.

[26] N. Anand, K. H. P. Reddy, G. V. S. Prasad, K. S. R. Rao, D. R. Burri, *Catal. Commun.* **2012**, *23*, 5-9.

[27] R. Xie, G. Fan, L. Yang, F. Li, *ChemCatChem* **2016**, *8,* 363-371.

[28] Y. Chen, S. Jie, C. Yang, Z. Liu, *Appl. Surf. Sci.* **2017**, *419*, 98-106.

[29] L. Wang, Y. Zhu, J. Q. Wang, F. Liu, J. Huang, X. Meng, J. M. Basset, Y. Han, F. S. Xiao, *Nat. Commun.* **2015**, *6*.

[30] W. Liu, L. Zhang, X. Liu, X. Liu, X. Yang, S. Miao, W. Wang, A. Wang, T. Zhang, *J. Am. Chem. Soc.* **2017**, 139, 10790−10798.

[31] Y. Zhu, W. Sun, W. Chen, T. Cao, Y. Xiong, J. Luo, J. Dong, L. Zheng, J. Zhang, X. Wang, C. Chen, Q. Peng, D. Wang, Y. Li, *Adv. Funct. Mater.* **2018**, *28*, 1802167.
